# Supplementary material for: Cost-effectiveness of a dietary and physical activity intervention in adolescents: a prototype modelling study based on the Engaging Adolescents in Changing Behaviour (EACH-B) programme
Source: BMJ Open. 2022 Aug 4;12(8):e052611. doi: 10.1136/bmjopen-2021-052611 (PMC9362792; doi:10.1136/bmjopen-2021-052611)
Supplement: Supplementary data [file bmjopen-2021-052611supp001.pdf]

1 APPENDIX A

Model parameters were estimated by using the following equations. For the control arm, rate was based upon age only. For the intervention arm, rate was based on age and the change in BMI from the control population to the intervention.

Table 1: Parameter equations

Parameter	Equation	Units	Source
BMI - men	-0.0031*age^2+0.4096*age+16.098	Kg/m2	HSE 2017[1]
BMI - women	-0.0032*Age^2 + 0.4235*Age+15.812	Kg/m2	
Type 2 Diabetes Men Control, age <25y	0.025*Age-0.265	Incidence per 1000	Sharma 2015[2]
Type 2 Diabetes Men Control, age >25y	0.0054Age^2-0.191Age+1.7142	Incidence per 1000	
Type 2 Diabetes Women, Control, all ages	0.0031Age^2-0.0942Age+1.2045	Incidence per 1000	
Type 2 Diabetes Men, Intervention, age < 25y	(0.025*Age-0.265)/(0.081BMI_control-0.8451)*(0.081BMI_int-0.8451)	Incidence per 1000	Gray et al 2015[3]
Type 2 Diabetes Men, Intervention, > 25y	(0.0054Age^2-0.191Age+1.7142)/(0.081BMI_control-0.8451)*(0.081BMI_int-0.8451)	Incidence per 1000	
Type 2 Diabetes Women Intervention, all ages	(0.0031Age^2-0.0942Age+1.2045)/(0.0637BMI_control -0.3135)*(0.0637BMI_Int-0.3135)	Incidence per 1000	
Mental health Men, control	-0.00008*Age^2+0.0073*Age-0.0544	% Incidence	APMS 2014[4]
Mental health Women, control	-0.00007*Age^2+0.0062*Age+0.0034	% Incidence	
Mental Health Men, intervention	(-0.00007*Age^2+0.0062*Age+0.0034)*(0.0127*BMI_I+0.727)/(0.0127*BMI_C +0.727)	% Incidence	Sutaria et al 2018[5]
Mental Health Women, intervention	(-0.00007*Age^2+0.0062*Age+0.0034) *(0.0391*BMI_I+0.1087)/(0.0391*BMI_C+0.1087)	% Incidence	
Mental Health, diabetes	Multiply MH risk by 1.33		Chireh et al 2019[6]
Mental Health, Physical exercise	OR 0.83 high activity vs low activity. Applied to intervention arm only.		Schuch et al 2018[7]
Preterm birth <33 weeks, control	-0.0512*Age^2+3.0922*Age-37.891	Incidence per 1000	ONS 2017[8]
Preterm birth <33 weeks, intervention	(-0.0512*Age2+3.0922*Age-37.891)*(0.0514*BMI_I-0.1712)/(0.0514*BMI_C-0.1712)	Incidence per 1000	Mcdonald et al 2010[9]
Loss of earnings, men	12534BMI – 297703	£ over lifetime	

Parameter	Equation	Units	Source
Loss of earning, women	17695BMI - 439796	£ over lifetime	Hamilton et al 2018[10]

Table 2: Parameter equations for z-scores

Parameter	Equation	Units	Source
Mental Health Men, Intervention	(0.0054Age^2-0.191Age+1.7142)/(0.0274BMI_z_score_control+1.0011)*(0.0274BMI_z_scores_int+1.011)	Incidence per 1000	Gray et al 2015[3]
Mental Health Women, Intervention	(0.0031Age^2-0.0942Age+1.2045)/(0.084BMI_z_scores_control+ 0.9486)*(0.084BMI_z_scores_Int+0.9486)	Incidence per 1000	
Type 2 Diabetes Men, intervention	(-0.00007*Age^2+0.0062*Age+0.0034)/(0.1742BMI_z_scores_control+ 0.8964)*(0.1742BMI_z_scores_Int+0.8964)	% Incidence	Sutaria et al 2018[5]
Type 2 Diabetes Women, intervention	(-0.00007*Age^2+0.0062*Age+0.0034) *(0.1369*BMI_z_scores_I+1.0556)/(0.1369*BMI_z_scores_C+1.0556)	% Incidence	
Preterm birth <33 weeks, intervention	(-0.0512*Age2+3.0922*Age-37.891)*(0.1105*BMI_z_scores_I+0.9336)/(0.1105*BMI_z_scores_C+0.9336)	Incidence per 1000	Mcdonald et al 2010[9]

Table 3: Parameter equation for quality of life estimates

Parameter	Equation	Source
Quality of life, by age	0.9508566+0.0212126*Male-0.0002587*Age-0.0000332*Age^2	Maheswaran et al 2012[11]
Disutility for BMI, if BMI < 30	-0.0009BMI + 0.0189	
Disutility for BMI, if BMI > 30	-0.0036BMI+0.094	

Kalita N, *et al. BMJ Open* 2022; 12:e052611. doi: 10.1136/bmjopen-2021-052611

Table 4: Input parameters used in the model

Parameter	Value	SD/SE	95% CI-Low	95% CI-High	Distribution
**Direct trial efficacy**					
BMI effect size: Physical activity	0.13	0.046	0.040	0.220	Beta
BMI effect size: Dietary quality	0.04	0.01423	0.012	0.068	Beta
BMI z-scores effect size: Physical activity	0.04	0.02	0.001	0.079	Beta
BMI z-scores effect size: Dietary quality	0.02	0.01	0.000	0.040	Beta
Prop. meeting PA guidelines control	0.13	0.0013	0.127	0.133	Beta
Prop. meeting PA guidelines intervention	0.16	0.049	0.117	0.257	Beta
Increase in Moderate to Vigorous PA	4.84	2.944	-0.940	10.610	Beta
**Relative effectiveness**					
OR for depression for Low vs High Physical Activity	0.83	0.026	0.790	0.880	Beta
OR of diabetes increasing the risk of depression	1.33	0.133	1.069	1.591	Lognormal
RR of PA on incidence of diabetes	0.74	0.074	0.595	0.885	Lognormal
**Mortality**					
Mental health: risk of death by suicide	0.001	0.000	0.001	0.001	Beta
HR of mortality those with type 2 Diabetes aged <65 years	2.978	0.190	2.605	3.350	Lognormal
HR of mortality those with type 2 Diabetes aged >65 years	1.958	0.050	1.860	2.056	Lognormal
**Relative effectiveness[a]**					
Pre-term birth relative risk for BMI P1	0.051	0.005	0.041	0.061	Beta
Pre-term birth relative risk for BMI P2	-0.171		0.111	-0.322	
MH BMI relative risk Boys P1	0.013	0.004	0.004	0.021	Beta
MH BMI relative risk Boys P2	0.727		0.914	0.540	
MH BMI relative risk girls P1	0.039	0.004	0.031	0.047	Beta
MH BMI relative risk girls P2	0.109		0.324	-0.005	
Diabetes relative risk boys P1	0.081	0.004	0.073	0.089	Beta
Diabetes relative risk boys P2	-0.845		-0.571	-0.912	
Diabetes relative risk girls P1	0.064	0.013	0.039	0.089	Beta

Kalita N, *et al. BMJ Open* 2022; 12:e052611. doi: 10.1136/bmjopen-2021-052611

Parameter		Value	SD/SE	95% CI-Low	95% CI-High	Distribution
	Diabetes relative risk girls P2	-0.314		0.167	-0.906	
	Loss of earnings Men P1	12036	601.8	10856	13215.5	Gamma
	Loss of earnings Men P2	-284478		-256213	-311886.5	
	Loss of earnings Women P1	16689	1668.9	13418	19960.0	Gamma
	Loss of earnings Women P2	-412424		-339474	-504989.1	
**Z scores [a]**						
	Pre-term birth relative risk for BMI z-scores P1	0.1105	0.011	0.089	0.132	Beta
	Pre-term birth relative risk for BMI z-scores P2	0.9336		-0.910	-1.841	
	MH BMI z-scores relative risk Boys P1	0.0274	0.003	0.022	0.033	Beta
	MH BMI z-scores relative risk Boys P2	1.0011		0.526	0.295	
	MH BMI z-scores relative risk girls P1	0.084	0.008	0.068	0.100	Beta
	MH BMI z-scores relative risk girls P2	0.9486		-0.452	-1.160	
	Diabetes relative risk boys P1	0.1742	0.009	0.157	0.191	Beta
	Diabetes relative risk boys P2	0.8964		-2.378	-3.112	
	Diabetes relative risk girls P1	0.1369	0.027	0.083	0.191	Beta
	Diabetes relative risk girls P2	1.0556		-0.790	-3.097	
**Proportions of population with mental health conditions**						
	Chronic	0.170	0.017	0.137	0.203	Dirichlet
	intermittent	0.400	0.040	0.322	0.478	Dirichlet
	New and one-off	0.430	0.043	0.346	0.514	Dirichlet

[a] Risk equation is in the form: Relative risk = P1*BMI + P2

Kalita N, *et al. BMJ Open* 2022; 12:e052611. doi: 10.1136/bmjopen-2021-052611

2 APPENDIX B

A breakdown of the resources used for estimating the costs of the intervention i.e. LifeLab Plus is presented below. The intervention comprises of three components:

i. Life Lab
ii. Digital App
iii. Teachers' Support (Healthy Conversation skills)

The costs are estimated for one-year that is, for the trial duration. The assumptions related to resources-used are based on a discussion with KTW on 5th June 2019.

Table 5: Life Lab

Item	Number	Comment
Number of classes per year	50	
No of pupils in each class	25	Varies between 25-32; assumed 25 for the base case
Total pupils	1250	=50 classes x 25 pupils per class
No of schoolteachers in each class	1	
Total teachers	50	

Table 6: Staff Training for Life Lab and Healthy Conversation Skills

Item	Number	Comment
Lifelab staff / training day (days)	6	Each training day / Lifelab day consists of 6 staff days to run. 4 staff days to run training and preparation and 2 days of administration.
Teacher training		
No. of Training days	5	Each training day is for 10 teachers.
Lifelab Staff days	30	=5 training days x 6 staff days / training day
Lifelab		
Number of days	50	=50x1
Lifelab Staff days	300	=50 life lab days x 6 staff day / lifelab
Scientists		Each lifelab day consists of 3 scientists
Number trained	75	Assumed each scientist does two lifelab days
Training days	3	=75 scientists /25; 25 trained per day
Lifelab Staff days	18	=3 training days x 6 staff days / training day
Training the trainers (Healthy Conversation Skills)		
Trainers	6	Assumption
Lifelab Staff days	1	Assume takes 2 hours
Showcase		All schools
Lifelab Staff days	3.5	1.5 days prep; 1 day delivery for 2 staff
Total lifelab staff days	352.5	Band 4 teacher

Kalita N, *et al. BMJ Open* 2022; 12:e052611. doi: 10.1136/bmjopen-2021-052611

Data collection – Lifelab

Each class has two hours of data collection by two Lifelab staffs, 2 research nurses and 1 research assistant.

Table 7: Costs for data collection

Item	Number	Comment
Lifelab staff days	28.6	=2 hours x2 staff x50 (lifelab days)/7 (hours)
Research nurse	28.6	=2 hours x2 staff x50 (lifelab days)/7 (hours)
Research assistant	14.3	=2 hours x1 staff x50 (lifelab days)/7 (hours)

Table 8: Staff unit costs

Staff costs	Salary	Comment
Band 4 teacher	£34,189	i.e. £163 per day, Assumes 210 working days per year
Research assistant	£27,025	i.e. £129 per day, Assumes 210 working days per year
Research nurse Band 5	£26,231	i.e £125 per day, Assumes 210 working days per year

Table 9: Staff total costs

Staff costs	Total	Comment
Lifelab / training	£71,736	=£34,189/210x352.5*1.25;<br>= salary / working days per year*lifelab staff days*NI and pension adjustment<br>Assumes 210 working days per year, pension / NI contributions of 25%
Follow-up data collection	£12,641	=(£34,189*28.6 + £27,025 *28.6 + 26,231 *14.3)/210*1.25<br>= (salary teacher* data collection days teacher + salary research nurse* data collection days research nurse + salary research assistant*data collection days research assistant)/working days per year*NI / pension adjustment
Total	£84,377	

Table 10: Digital App development

Item	Total	Comment
Development of app	£324,000	Estimate from expert
Duration of development, year	5	As above
Duration of app, years	10	Assumption
Number of centres using app	10	Assumption
Cost per year development	£64,800	£324,000 / 5; total cost / number of years of development
Maintenance cost	£16,200	Assumed to be 25% of development cost
Cost per year of development across app lifetime per centre	£3240	£324,000 / 100; total development cost / (number of years app lasts for x number of centres)
EACH-B development for new centre	£87,445	Assumed to be 1 year of staff costs.

Item	Total	Comment
Number of years centre lasts	10	Assumption
Cost per year of centre	£8745	£87,445 / 10

Table 11: Summary of included costs

Item	Total	Comment
Staff costs	£84,377	
Room hire	£26,857	=400 (room hire cost)*67.14 (number of days); assumes cost of hire is £400 / day
Consumables	£15,839	Consumables include costs associated with printing, paper, classroom
Overheads	£37,970	Direct overheads based on 29% of direct care salary costs and indirect overheads based on 16% direct care salary costs (As used in Curtis et al 2019 for overheads for healthcare professionals)
Maintenance of app	£16,200	See above table
Capital cost, development of app	£3240	See above
Capital cost, development of EACH-B	£8745	See above

Table 12: Total costs

Item	Total	Comment
Total cost	£193,228	Sum of all of the above costs
Cost per pupil	£154.58	=£193,228/1250; (total cost /total number of pupils)

Kalita N, *et al. BMJ Open* 2022; 12:e052611. doi: 10.1136/bmjopen-2021-052611

3 APPENDIX C

Figure 1: Cost-Effectiveness Acceptability Curve (CEAC)

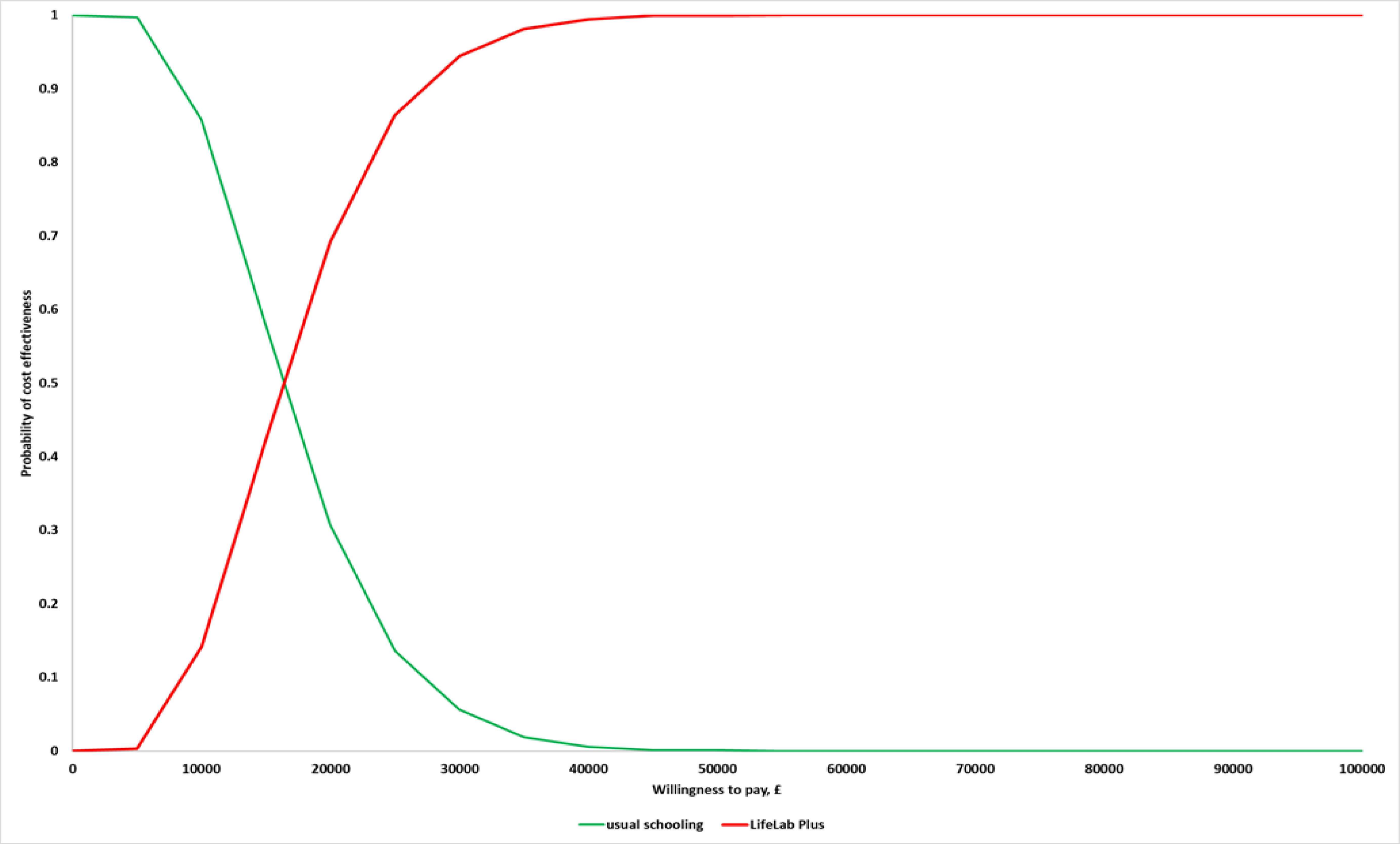

REFERENCES

1. NHS Digital. Health Survey for England 2016. 2017.
2. Sharma M, Nazareth I, Petersen I. Trends in incidence, prevalence and prescribing in type 2 diabetes mellitus between 2000 and 2013 in primary care: a retrospective cohort study. *BMJ Open* 2016;6(1):e010210. doi: 10.1136/bmjopen-2015-010210 [published Online First: 2016/01/16]
3. Gray N, Picone G, Sloan F, et al. Relation between BMI and diabetes mellitus and its complications among US older adults. *South Med J* 2015;108(1):29-36. doi: 10.14423/SMJ.0000000000000214 [published Online First: 2015/01/13]
4. McManus S BP, Jenkins R, Brugha T. Mental health and wellbeing in England: Adult Psychiatric Morbidity Survey 2014. 2016.
5. Sutaria S, Devakumar D, Yasuda SS, et al. Is obesity associated with depression in children? Systematic review and meta-analysis. *Arch Dis Child* 2019;104(1):64-74. doi: 10.1136/archdischild-2017-314608 [published Online First: 2018/07/01]
6. Chireh B, Li M, D'Arcy C. Diabetes increases the risk of depression: A systematic review, meta-analysis and estimates of population attributable fractions based on prospective studies. *Prev Med Rep* 2019;14:100822. doi: 10.1016/j.pmedr.2019.100822 [published Online First: 2019/03/01]
7. Schuch FB, Vancampfort D, Richards J, et al. Exercise as a treatment for depression: A meta-analysis adjusting for publication bias. *J Psychiatr Res* 2016;77:42-51. doi: 10.1016/j.jpsychires.2016.02.023 [published Online First: 2016/03/16]
8. Office of National Statistics. Births in England and Wales: 2017. 2018.
9. McDonald SD, Han Z, Mulla S, et al. Overweight and obesity in mothers and risk of preterm birth and low birth weight infants: systematic review and meta-analyses. *BMJ* 2010;341:c3428. doi: 10.1136/bmj.c3428 [published Online First: 2010/07/22]
10. Hamilton D, Dee A, Perry IJ. The lifetime costs of overweight and obesity in childhood and adolescence: a systematic review. *Obes Rev* 2018;19(4):452-63. doi: 10.1111/obr.12649 [published Online First: 2017/12/23]
11. Maheswaran H, Petrou S, Rees K, et al. Estimating EQ-5D utility values for major health behavioural risk factors in England. *J Epidemiol Community Health* 2013;67(2):172-80. doi: 10.1136/jech-2012-201019 [published Online First: 2012/07/31]
