# Supplementary material for: Cost-effectiveness of a dietary and physical activity intervention in adolescents: a prototype modelling study based on the Engaging Adolescents in Changing Behaviour (EACH-B) programme
Source: BMJ Open. 2022 Aug 4;12(8):e052611. doi: 10.1136/bmjopen-2021-052611 (PMC9362792; doi:10.1136/bmjopen-2021-052611)
Supplement: Author's manuscript [file bmjopen-2021-052611.draft_revisions.pdf]

BMJ Open is committed to open peer review. As part of this commitment we make the peer review history of every article we publish publicly available.

When an article is published we post the peer reviewers' comments and the authors' responses online. We also post the versions of the paper that were used during peer review. These are the versions that the peer review comments apply to.

The versions of the paper that follow are the versions that were submitted during the peer review process. They are not the versions of record or the final published versions. They should not be cited or distributed as the published version of this manuscript.

BMJ Open is an open access journal and the full, final, typeset and author-corrected version of record of the manuscript is available on our site with no access controls, subscription charges or pay-per-view fees (http://bmjopen.bmj.com).

If you have any questions on BMJ Open's open peer review process please email info.bmjopen@bmj.com

BMJ Open

Do dietary and physical activity interventions in adolescents provide a cost-effective use of healthcare resources? Model development and illustration based on the Engaging Adolescents in Changing Behaviour (EACH-B) programme

Journal:	*BMJ Open*
Manuscript ID	bmjopen-2021-052611
Article Type:	Original research
Date Submitted by the Author:	20-Apr-2021

Complete List of Authors:	Kalita, Neelam; University of Southampton, Southampton Health Technology Assessments Centre<br>Cooper, Keith; Univ Southampton, SHTAC<br>Baird, Janis; University of Southampton, MRC Epidemiology Resource Centre; University of Southampton, NIHR Southampton Biomedical Research Centre<br>Woods-Townsend, Kathryn ; University of Southampton, Southampton Education School, Faculty of Social Sciences; University of Southampton , NIHR Southampton Biomedical Research Centre<br>Godfrey, Keith; University of Southampton and Southampton University Hospitals NHS Trust, MRC Lifecourse Epidemiology Unit; NIHR Southampton Biomedical Research Centre<br>Cooper, Cyrus; University of Southampton and Southampton University Hospitals NHS Trust, MRC Lifecourse Epidemiology Unit; University of Oxford, Nuffield Department of Orthopaedics, Rheumatology and Musculoskeletal Sciences<br>Inskip, Hazel; Medical Research Council, Epidemiology Resource Centre; NIHR Southampton Biomedical Research Centre<br>Barker, Mary; University of Southampton, MRC Lifecourse Epidemiology Unit; NIHR Southampton Biomedical Research Centre<br>Lord, Joanne; University of Southampton, Southampton Health Technology Assessments Centre

Keywords:	HEALTH ECONOMICS, MENTAL HEALTH, NUTRITION & DIETETICS, General diabetes < DIABETES & ENDOCRINOLOGY

SCHOLARONE™
Manuscripts

I, the Submitting Author has the right to grant and does grant on behalf of all authors of the Work (as defined in the below author licence), an exclusive licence and/or a non-exclusive licence for contributions from authors who are: i) UK Crown employees; ii) where BMJ has agreed a CC-BY licence shall apply, and/or iii) in accordance with the terms applicable for US Federal Government officers or employees acting as part of their official duties; on a worldwide, perpetual, irrevocable, royalty-free basis to BMJ Publishing Group Ltd ("BMJ") its licensees and where the relevant Journal is co-owned by BMJ to the co-owners of the Journal, to publish the Work in this journal and any other BMJ products and to exploit all rights, as set out in our licence.

The Submitting Author accepts and understands that any supply made under these terms is made by BMJ to the Submitting Author unless you are acting as an employee on behalf of your employer or a postgraduate student of an affiliated institution which is paying any applicable article publishing charge ("APC") for Open Access articles. Where the Submitting Author wishes to make the Work available on an Open Access basis (and intends to pay the relevant APC), the terms of reuse of such Open Access shall be governed by a Creative Commons licence – details of these licences and which Creative Commons licence will apply to this Work are set out in our licence referred to above.

Other than as permitted in any relevant BMJ Author's Self Archiving Policies, I confirm this Work has not been accepted for publication elsewhere, is not being considered for publication elsewhere and does not duplicate material already published. I confirm all authors consent to publication of this Work and authorise the granting of this licence.

For peer review only

Do dietary and physical activity interventions in adolescents provide a cost-effective use of healthcare resources? Model development and illustration based on the Engaging Adolescents in Changing Behaviour (EACH-B) programme

Authors: Neelam Kalita[1], Keith Cooper[1], Janis Baird[2;3], Kathryn Woods-Townsend[3;4], Keith M Godfrey[2;3], Cyrus Cooper[2;6;7], Hazel Inskip[2;3], Mary Barker[2;3;5], Joanne Lord[1] and the EACH-B study group

Affiliations:

[1] Southampton Health Technology Assessments Centre, University of Southampton, UK

[2] MRC Lifecourse Epidemiology Unit, University of Southampton, Southampton, UK

[3] NIHR Southampton Biomedical Research Centre, University of Southampton and University Hospital Southampton NHS Foundation Trust, Southampton, UK

[4] Southampton Education School, Faculty of Social Sciences, University of Southampton, Southampton, UK

[5] School of Health Sciences, University of Southampton, Southampton, UK

[6] Faculty of Medicine, University of Southampton

[7] Nuffield Department of Orthopaedics, Rheumatology and Musculoskeletal Sciences, University of Oxford

Corresponding author: Neelam Kalita

Email: N.Kalita@soton.ac.uk

ABSTRACT

Objective To assess costs, health outcomes and cost-effectiveness of interventions that aim to improve quality of diet and level of physical activity in adolescents.

Design A Markov model was developed to assess four potential benefits of healthy behaviour for adolescents: better mental health, Type 2 diabetes, higher earnings and reduced incidences of adverse pregnancy outcomes. The model parameters were informed by published literature. The analysis took a societal perspective over a 20-year period. One-way and probabilistic sensitivity analyses were conducted.

Setting Secondary schools.

Participants A hypothetical cohort of adolescents aged 12-13 years.

Interventions An exemplar school-based, multi-component intervention that was developed by the Engaging Adolescents for Changing Behaviour programme, compared with usual schooling.

Primary and secondary outcome measures Incremental cost-effectiveness ratio as measured by cost per quality-adjusted life year (QALY) gained.

Results The model suggested that an intervention for improving diet and physical activity has the potential to offer a cost-effective use of healthcare resources for adolescents in the UK at a willingness-to-pay threshold of £20,000 per QALY. The key model drivers are the intervention effect on levels of physical activity, quality of life gain for high levels of physical activity, the duration of the intervention effects and the period over which effects wane.

Conclusions The model focused on short to medium-term benefits of healthy eating and physical activity exploiting the strong evidence base that exists for this age group. Other benefits in later life, such as reduced cardiovascular risk, are more sensitive to assumptions about the persistence of behavioural change and discounting.

For peer review only - http://bmjopen.bmj.com/site/about/guidelines.xhtml

ARTICLE SUMMARY

Strengths and limitations of this study

- The study addresses an important public health question by examining if interventions targeting healthy eating and doing more physical activities provide value for money from a societal perspective.
- A Markov cohort model was developed to assess the costs and benefits, expressed in terms of quality-adjusted life years, of an exemplar school-based, multi-component intervention to improve adolescents' diet and increase their levels of physical activity.
- The study incorporates existing evidence on the effect of improvement in adolescent health behaviours on four high prevalence short-to-medium term benefits relevant to young people: improved mental health, higher earnings, improved pregnancy outcomes and prevention of Type 2 diabetes.
- Extensive sensitivity analyses were conducted to investigate the impact of uncertainty over the model assumptions and parameter inputs, thereby highlighting areas where further research would be most useful.
- A limitation of the study is that it does not estimate the long-term impacts of such interventions due to the lack of longitudinal data on lifetime trajectories of healthy diet and increased levels of physical activity.

Mesh terms: Adolescent, Cost-effectiveness, School-based, Exercise, Diet

For peer review only - http://bmjopen.bmj.com/site/about/guidelines.xhtml

INTRODUCTION

Poor diet and lack of physical activity increase the risk of non-communicable diseases (NCDs), including cardiovascular diseases, Type 2 diabetes and some cancers such as breast, colon and endometrial, in part by contributing to overweight and obesity.[1 2] Adolescence, the life stage between childhood and adulthood, is a critical period for the development of health and disease in later life.[3 4] Compared with other age-groups, adolescents have the unhealthiest diets and most (over 80%) fail to meet national guidelines for physical activity.[5-7] Furthermore, the proportion meeting recommended levels of physical activity has been declining, particularly among girls (from 14% to 8%, 2008-2012).[8]

The disease burden of poor diet and physical inactivity on healthcare services is significant. In the UK, poor diet and physical inactivity cost £7 billion to the National Health Service (NHS) annually.[9] Meeting current dietary recommendations would reduce years-of-life lost to coronary heart disease by 2 million, stroke by 400,000 and Type 2 diabetes by 19,000 over 20 years.[10]

Health behaviours in adolescence track into adulthood.[5 9 11 12] Therefore, sub-optimal diet and body composition in adolescence not only affect immediate physical and mental health but also increase the risk of NCDs in later life. Developmental plasticity in adolescence means that interventions to improve diet and levels of physical activity have the potential to reduce the trajectory of NCD risk over the life course.[13] While many adolescents find it difficult to engage with the long-term consequences of health behaviour, motivated and engaged adolescents can improve their health behaviours.[14 15] Evidence suggests that school-based interventions that offer combinations of peer-modelling, social support and choice, may be effective in improving diet and physical activity amongst adolescents.[16 17 18] Furthermore there is an increasing use of digital platforms by adolescents. According to 2018 estimates, 83% of 12-15 years olds own smartphones with 99% spending an average of 20 hours per week online.[19 20] With an explosion in the use of such platforms to influence health behaviours in young people, there are suggestions that they have potential as a complementary feature in complex interventions that aim to influence health behaviour in adolescents.[20]

Within this framework, a research programme Engaging Adolescents in CHanging Behaviour

For peer review only

(EACH-B) was designed to develop and test an intervention to encourage UK-based school students, aged 12-13 years, to adopt healthy behaviours such as eating better and exercising more (Trial registration: ISRCTN 74109264). EACH-B involves a cluster randomised controlled trial as a test of intervention effectiveness. Further details of the trial design are given elsewhere.[20] The research programme is funded by the National Institute for Health Research (RP-PG-0216-20004). The "LifeLab Plus" intervention developed as part of this programme is a complex three-part programme that comprises: i) an education module that teaches school students the science behind health messages through a 2-week module with a "hands-on" practical one-day visit to a teaching laboratory at University Hospital Southampton or in school while COVID-19 restrictions apply; ii) training for teachers in skills to support behaviour change; and iii) access to a specially-designed, interactive smartphone app with game features.

Despite an emerging interest in identifying and developing interventions for improving diet and physical activity levels in adolescents, there is sparse economic evidence assessing health effects and costs of such interventions. To address this gap, we developed an illustrative decision-analytic model to assess the health benefits, costs and cost-effectiveness of a multi-component intervention such as LifeLab Plus. This prototype model is designed to investigate how changes in diet quality and levels in physical activity could affect future health outcomes and costs. We used costs data from the EACH-B programme and effectiveness from published literature. The model will be updated when results from the EACH-B trial are available.

Although there is good epidemiological evidence of long-term tracking of health behaviour,[5 9 11 12] school-based trials rarely follow up for more than a year.[1 21 22] The persistence of intervention effects is therefore uncertain. We take a conservative approach and focus on potential impacts of improved diet and physical activity likely to manifest in the short to medium term: up to a maximum time horizon of 20 years. We also explore alternative assumptions about the persistence of effects on behaviour after trial follow-up.

METHODS

We developed a prototype *de novo* Markov model to estimate the costs, benefits and cost-effectiveness of school-based interventions that aim to improve diet quality and levels of

For peer review only - http://bmjopen.bmj.com/site/about/guidelines.xhtml

physical activity compared with usual schooling for a cohort of adolescents. The model focussed on four potential short to medium-term benefits of healthy eating and physical activity in this age group: better mental health outcomes, higher earnings and reduced incidences of adverse pregnancy outcomes and Type 2 diabetes.

The model assumed that improved diet quality and increased physical activity would impact these four health outcomes via reduction in BMI. Discussion with key project stakeholders reiterated these four benefits as the most relevant obesity-related effects in this population. The model did not include later life cardiovascular disease or other chronic diseases as outcomes since the likely impact on these of an intervention undertaken as an adolescent was uncertain. The model also investigated independent effects of physical activity on diabetes and depression (i.e. direct impacts not mediated by BMI). Information relating to epidemiology, mortality, effectiveness, health-related quality of life and costs was obtained from a variety of sources and used to inform the model parameters and assumptions. See **Error! Reference source not found.** for model illustration.

Structuring the model

Population

A cohort of adolescents with an equal proportion of boys and girls and a mean age of 13 years was entered into the model. The intervention is based on that developed by the EACH-B programme, i.e. LifeLab Plus.[20] The impact of this is compared with that of usual schooling.

Model states

The Markov model consisted of three health states: *No Type 2 Diabetes*, *Type 2 Diabetes* and *Death*. Outcomes associated with mental health, loss of earnings and adverse pregnancy outcome were incorporated as model events. Mental health encompasses a wide spectrum of conditions. Therefore, a pragmatic approach was adopted to include the two most relevant mental health events for adolescents: clinical depression (henceforth, referred as depression) and General Anxiety Disorder (GAD). These events were categorised as: chronic (history of persistent mental illness), intermittent (experiencing intermittent episodes), and new onset (a one-time episode). Adverse pregnancy outcome was defined by pre-term delivery categorised as: extremely pre-term (delivery <28 weeks); very pre-term (delivery <33 weeks); and

For peer review only - http://bmjopen.bmj.com/site/about/guidelines.xhtml

moderately pre-term (delivery between 34-36 weeks). The pregnancy outcome was applicable for girls only.

Adolescents enter the model in the *No Type 2 diabetes* health state. In each model cycle, a proportion of individuals develop Type 2 diabetes and move to the *Type 2 diabetes* health state. They may also experience mental health events or pre-term births (girls only), which are treated as transient states. Each health state and event are associated with a health-related quality of life (HRQoL) and excess cost estimate. The model included intervention cost associated with LifeLab Plus and healthcare costs associated with- Type 2 diabetes, mental health events, pre-term delivery and loss of earnings due to obesity. In each model cycle, the total costs and QALYs are calculated by multiplying the individual costs and HRQoL by the number of people in the cohort still alive for each of the intervention and control arms. The total lifetime costs and QALYs are calculated by aggregating the costs and QALYs for all cycles.

Persistence of effects and time horizon

For the base case, the duration of the intervention effect was assumed to be sustained for 4 years with the effect waning over a period of 10 years. The model time horizon was 20 years, with yearly cycle length. This was considered appropriate as the mean age of adolescents entering the model was 13 years as it is in the EACH-B trial. The persistence of behaviour change effects from such interventions remains unclear. Therefore, the treatment effect on behaviour was not assumed to last for 20 years. Costs and outcomes were half-cycle corrected.

Populating the model

Targeted literature searches were conducted to identify sources to inform model parameters. These are discussed below. For further details, see Appendix A and Appendix B.

Epidemiological data

Data on the relationship between mean BMI, age and sex was taken from Health Survey for England.[23] For adolescents aged ≤19 years, BMI z-scores are normally used. Therefore, we rescaled the values to relate relative risks to BMI z-scores, where:

For peer review only - http://bmjopen.bmj.com/site/about/guidelines.xhtml

BMI z-score = (observed value – median value of the reference population) / standard deviation value of reference population.

Physical activity levels for children aged 13-15 years were taken from Health Survey England, 2015.[24] The incidence of Type 2 diabetes in UK was based on an analysis of longitudinal electronic health records in the Health Improvement Network (THIN) primary care database.[25] The prevalence of depressive episodes and GAD was taken from the Adult Psychiatric Morbidity Survey 2014[26] and from Mental Health of Children and Young people in England 2017.[27] The proportion in each category was assumed as follows: 17% had a chronic and 40% had a fluctuating (intermittent) course, while 43% remitted (new one-time episode).[28] Those individuals with depression or anxiety are at higher risk of suicide than the general population.[29] The excess death rate for those with depression and anxiety was calculated by using the suicide rate in the UK from Office of National Statistics (ONS) 2017 and applying a relative risk of 10.9 for depression and anxiety.[29] The proportions of pre-term deliveries, obtained from ONS 2017 data, were assumed as follows: 0.5% of total births as extremely pre-term (<28 weeks), 1.2% very pre-term birth (28 to <33 weeks) and 6.3% moderately pre-term birth (33-36 weeks) respectively.[30]

Relationship between BMI and risks of health events

The economic model assumed a positive correlation between increased BMI and the risks of - Type 2 diabetes, depression and GAD, pre-term delivery and loss of earnings. We fitted equations to the BMI relative risks. Hazard ratios, obtained from the Medicare Current Beneficiary Survey 1991-2010, were used to estimate the increased risk of individuals with higher BMI developing Type 2 diabetes.[31] The odds of depression and GAD in obese and overweight adolescents compared with normal-weight adolescents were obtained from Sutaria et al.[32] This systematic review included 22 observational studies published between 2000 and 2017, representing 143,603 children. The relative risk of pre-term birth for mothers with overweight and obesity was obtained from Mcdonald et al.[33] The risk was assumed to be the same for all three type of pre-term births.

Relationship between physical activity and risk of health event

The direct effect of physical activity on developing depression is modelled independent of the effect of physical activity via BMI. The odds ratio of developing depression was assumed to be 0.83 (95% CI 0.79-0.88) in those with high levels of physical activity compared to those

For peer review only - http://bmjopen.bmj.com/site/about/guidelines.xhtml

with lower levels.[34] Furthermore, an increase from being inactive to achieving the recommended physical activity level (150 minutes of moderate-intensity aerobic activity per week) was assumed to lower the risk of Type 2 diabetes incidence by 26%, after adjustment for body weight.[35] The pooled odds ratio between Type 2 diabetes and risk of depression was 1.33 (95% CI, 1.18-1.51).[36]

Intervention effect

The intervention effect was based on three systematic reviews and meta-analyses that estimated the overall effects of school-based obesity prevention interventions.[37 38 39] The results of the meta-analyses were found to be significantly different between groups based on BMI (− 0.17 (95% CI− 0.29, − 0.06) kg/m^2)[38] and BMI z-score (− 0.06 (95% CI -0.10, − 0.03))[39] for multi-component interventions including physical activity, health education and dietary improvement. The effect of school-based intervention was assumed to increase the level of moderate or vigorous physical activity in children and adolescents by 4.84 min/day (95% CI −0.94 to 10.61).[40]

Mortality

General population mortality, adjusted for age and gender, was based on ONS 2020.[41] A hazard ratio of mortality of 2.98 was applied for individuals with Type 2 diabetes and aged <65 years.[42]

Quality of life

EQ-5D estimates adjusted for age, gender and BMI were used to estimate quality of life. See **Error! Reference source not found.**. These estimates were obtained from 14,117 participants aged ≥16 years from the Health Survey for England 2008.[43] Adults with diabetes was assumed to have a disutility of -0.161 where the pooled mean EQ-5D score for individuals with Type 2 diabetes was 0.67 at a mean age of 60 years.[44] We estimated the disutility by comparing the mean general population EQ-5D score with that for diabetes. A disutility of -0.087 is used for adolescents with Type 2 diabetes based on a Swedish cohort of adolescents aged 13-18 years.[45] A utility decrement of 0.188 was assumed for those with intermittent episodes of mental health conditions;[46] a decrement of 0.488 for those with persistent/chronic depression;[47] a decrement of 0.094 (half of the decrement for intermittent depression) was assumed for those with a new episode of depression. For pre-term delivery, a

For peer review only

For peer review only - http://bmjopen.bmj.com/site/about/guidelines.xhtml

mean utility decrement of 0.066 was applied throughout the model time horizon. This was based on a systematic review and meta-analysis for health utility values associated with pre-term birth where all but one study used Health Utilities Index (HUI) Mark 2 (HUI2) or Mark 3 (HUI3) measures as their primary health utility assessment method.[48] We found no evidence for quality of life loss in parents of pre-term babies. Therefore, we assumed that the quality of life decrement would be similar to intermittent mental health condition and lasts for the first two years.

Costs

Intervention costs

The intervention cost for our illustrative analysis was based on LifeLab Plus. Further information on resources used for delivering LifeLab are in Appendix B. The cost of the application used delivered as part of the intervention, was incorporated as a capital cost and was assumed to last 10 years and be used in 10 centres. Similarly, the cost of setting up LifeLab Plus in a different centre was assumed to consist of one year's staff costs and assumed to last for 10 years. Maintenance costs were estimated at 25% of the development cost per year. Overheads were included according to the rates used in Curtis et al. 2019[49], i.e. direct overheads based on 29% of direct care salary costs and indirect overheads based on 16% direct care salary costs.

Health state costs

Literature for costing studies and national datasets relating to individuals with Type 2 diabetes, depression, pre-term birth, and loss of earnings related to obesity were reviewed to inform the cost parameters. Both direct and indirect costs were included. Indirect costs included the effect of depression on income and productivity. Costs were updated to 2019 prices using the hospital and community health services index.[49] See **Error! Reference source not found.**.

The current and future costs for Type 2 diabetes were sourced from Hex et al.[50] Direct health costs and indirect societal and productivity costs were estimated using a top-down approach. We assumed that individuals with diabetes would not incur costs for complications as these are likely to affect individuals who have diabetes for a longer period. Direct and indirect costs for depression were taken from a trial of patients with a history of recurrent depression

For peer review only - http://bmjopen.bmj.com/site/about/guidelines.xhtml

by Kuyken et al.;[51] and the costs associated with pre-term birth were based on the study by Khan et al.[52]

Total societal costs for children born at 32-33 weeks and 34-36 weeks gestation from birth to 24 months were taken from a study that compared these costs to those for children born at full-term. Costs for children born <28 weeks and 28-33 weeks were estimated, assuming that they varied in the same way.[53] The cost used in the model was the mean societal cost difference between these two groups.

The lifetime indirect costs for overweight and obesity in childhood and adolescence is based on the study by Hamilton et al.[54] Mean total lifetime healthcare and productivity costs were estimated in Irish Euros, which were converted to GBP (£) and adjusted according to the average wage in the UK.

Validation

The structure of the prototype model was validated by the study team comprising epidemiologists, statisticians, trialists, public health experts and health economists. Internal validity of the model was established through running several tests and ensuring that the model predictions were consistent with the model specification.

Cost-effectiveness analysis

We followed the modelling guidelines advocated by NICE Reference case,[55] with an exception for the perspective adopted for the analysis. The economic evaluation was conducted from a societal perspective that included both direct and indirect costs. Costs and benefits were discounted at 3.5% per year and expressed in terms of QALYs. These were estimated and averaged across the simulated cohort. The incremental cost-effectiveness ratio (ICER) was estimated as a ratio of the incremental costs of the intervention, i.e. LifeLab Plus relative to the comparator (usual schooling) to the incremental QALYs of the intervention relative to the comparator. The intervention was considered cost-effective if the ICER was below the lower (more conservative) threshold of £20,000 per QALY gained, as recommended for the English NHS by NICE.[55]

Sensitivity analyses

To assess the uncertainty around the model predictions, we conducted deterministic

For peer review only - http://bmjopen.bmj.com/site/about/guidelines.xhtml

sensitivity analysis (DSA), probabilistic sensitivity analyses (PSA) and scenario analyses. For the DSA, input parameters were varied by 95% confidence intervals where available. Monte Carlo simulations of 1000 iterations were run for the PSA to assess the combined effects of input parameter uncertainties where parameters were simultaneously sampled within a specified distribution. See Table 1. Further details in Appendix A. Scenario analyses were conducted to assess structural uncertainties related to model assumptions. Uncertainty about the sustainability of the intervention effect was assessed by varying the duration of intervention effect and its waning period.

Effectiveness parameters were assigned beta and lognormal distributions, utilities were assumed to follow a beta distribution, and costs were assigned a gamma distribution. The model was developed and implemented in Microsoft Excel.

Patient and public involvement

The research questions addressed in the overarching research programme EACH-B were informed by public involvement. Furthermore, representatives from public involved in the EACH-B research programme were presented the conceptual framework, modelling approaches and invited to comment.

RESULTS

Base case analysis

In the base case analysis, the exemplar intervention based on the multi-component LifeLab Plus was associated with higher costs and better health outcomes (more QALYs) compared with usual schooling. LifeLab Plus was associated with a mean QALY gain of 0.0085 at an incremental cost of £123 per person compared with usual schooling, resulting in an ICER of £14,367 per QALY. See **Error! Reference source not found.**.

Sensitivity analyses

As outlined in the methods section, sensitivity analyses were implemented by varying the base case assumptions and parameter inputs. The result of the one-way sensitivity analyses is presented as a Tornado plot. See **Error! Reference source not found.**. Parameters such as the intervention effect on levels of physical activity (expressed in terms of minutes of

For peer review only

For peer review only - http://bmjopen.bmj.com/site/about/guidelines.xhtml

moderate or vigorous physical activity), quality of life gain for high levels of physical activity, duration of intervention effect and duration of treatment waning period had the highest impact on the cost-effectiveness results. Other parameters such as effect of physical activity on BMI, time horizon and intervention costs also influenced the base case results, but to a lesser extent. The results of the PSA presented as the cost-effectiveness scatter plot showed that the simulations lie in the North-East quadrant of the cost-effectiveness plane. See **Error! Reference source not found.**. This implies that the intervention- LifeLab Plus is likely to produce health benefits at an additional cost. At a willingness-to-pay threshold of £20,000 per QALY gained, the probability of LifeLab Plus being cost-effective was 69% compared with usual schooling at 31% respectively.

DISCUSSION

Main findings

Based on economic modelling, we estimate that a multi-component intervention to improve dietary quality and physical activity, such as LifeLab Plus, would be considered cost-effective under conventional willingness-to-pay thresholds of £20,000–£30,000 per QALY gained in the UK. For our base case, the duration of the treatment effect was assumed to be sustained for 4 years with the effect waning over a further period of 10 years. Our sensitivity analyses showed that if the duration of the treatment effect was not sustained to this extent, the intervention would be less cost-effective. See **Error! Reference source not found.**.

Comparison with previous models

Previous cost-effectiveness studies evaluating dietary and physical activity interventions for adolescents have largely consisted of within trial analyses that have not considered the benefits beyond the trial period.[56-60] The cost-effectiveness of these intervention vary between cost saving of NZ$835 per child for the low intensity program[56] to £120,630 per QALY for the HELP intervention.[59] However, comparison between studies is difficult because of the differences in the study designs, the interventions considered and the outcomes reported.

Gc et al[1] developed a model to assess the long-term costs and health outcomes of two physical activity interventions targeting adolescents in UK. The cost-effectiveness of these interventions varied between £11,426 per QALY for an after-school intervention and £68,056 per QALY for a multicomponent intervention. The costs of these interventions varied

For peer review only - http://bmjopen.bmj.com/site/about/guidelines.xhtml

between £51 per participant for the after-school intervention to AUS$394 for the multicomponent intervention. Their model included different health states to our model for diseases which typically affect people in later life such as chronic heart disease, stroke, heart failure, breast failure and colorectal cancer. They ran the model for a lifetime horizon of 65 years. We have not included these health states as our cost-effectiveness results are based on a conservative assumption that treatment benefits for adolescents from such multicomponent intervention as LifeLab Plus do not persist beyond 20 years.

Strengths and limitations

The study addresses an important public health question by examining if interventions targeting healthy eating and doing more physical activities provide value for money from a societal perspective. The study incorporates existing evidence on the effect of improvement in adolescent health behaviours on four high prevalence short-to-medium term benefits relevant to young people: improved mental health, higher earnings, improved pregnancy outcomes and prevention of Type 2 diabetes. Sources for data used within our model were identified from a targeted literature review. Where data were not available for adolescents, we have used data from the adult population. Model structure and assumptions were informed by this review and discussions with public health experts. UK-specific incidence rates were used to ensure that patients entering the model matched the likely distribution of events in the UK.

The model does not estimate the long-term impacts of such interventions due to the lack of longitudinal data on lifetime trajectories of healthy diet and increased levels of physical activity. If the effects are more lasting, then additional benefits such as prevention of cardiovascular disease would enhance the cost-effectiveness of the intervention. Per se our cost-effectiveness results are conservative.

Our model has several shortcomings. Firstly, there is uncertainty around key assumptions related to the duration of benefits from the intervention. In our analysis, we have assumed that the benefit observed in the clinical trials will last for 4 years and then will gradually reduce over the next 10 years. We only include costs and effects over a 20-year time horizon and have assumed that there would be no further benefits of the intervention for chronic diseases such as cardiovascular diseases and diabetes. Secondly, there are limitations in our approach to estimating income lost due to obesity. We have adopted a simple approach;

For peer review only - http://bmjopen.bmj.com/site/about/guidelines.xhtml

For peer review only

however, this is a complex interacting bi-directional system. We have not fully explored whether income loss is due to obesity or whether obesity is caused by income loss through, for example, unemployment. Finally, where data from adolescent age groups were unavailable, those from adult populations were used to inform model parameters.

CONCLUSION

Complex behavioural interventions that aim to improve diet and increase levels of physical activity amongst school-aged children have the potential to provide cost-effective use of UK healthcare resources. Such interventions have the potential to reduce burden of NCDs, although benefits in later life are more sensitive to assumptions about the persistence of behavioural change and discounting. Lastly, there is a need to establish long-term (preferably, lifetime) effectiveness of such interventions.

Word count: 3987

Author Contributions NK designed the model, performed the analysis, interpreted the analysis, and wrote the first draft of the manuscript. KC designed the model and performed the analysis. JL supervised the process, provided comments on model structure and data analysis. KWT provided inputs for model parameters. JB, KMG, HI, MB and CC provided critical comments on the model structure. All authors contributed to the critical revision of the manuscript and approved the final version of the manuscript.

Funding The research is funded by the National Institute for Health Research (RP-PG-0216-20004). HMI and KMG are funded by the UK Medical Research Council (MC_UU_12011/4). KMG is supported by the National Institute for Health Research (NIHR Senior Investigator (NF-SI-0515-10042), NIHR Southampton 1000DaysPlus Global Nutrition Research Group (17/63/154) and NIHR Southampton Biomedical Research Centre (IS-BRC-1215-20004)), the European Union (Erasmus+ Programme ImpENSA 598488-EPP-1-2018-1-DE-EPPKA2-CBHE-JP) and the British Heart Foundation (RG/15/17/3174).

Competing Interests KMG has received reimbursement for speaking at conferences sponsored by companies selling nutritional products, and is part of an academic consortium that has received research funding from Abbott Nutrition, Nestec, BenevolentAI Bio Ltd. and Danone. The other authors have no potentially competing interests to declare.

Patient consent for publication Not required

Data availability statement Data from publicly available sources were used for the model. All model inputs are described in the paper.

For peer review only

Table 1 Input parameters used in the model

Parameter	Value	SD/SE	95% CI-Low	95% CI-High	Distribution
**Utility inputs**					
Disutility for Diabetes for 13-18 years old	0.087	0.036	0.016	0.158	Beta
Disutility for Diabetes	0.161	0.040	0.240	0.080	Beta
Disutility for pre-term babies < 33 weeks	-0.066	0.016	0.098	0.035	Beta
Disutility for parents of pre-term babies < 33 weeks	-0.094	0.019	0.057	0.131	Beta
Disutility for depression	-0.188	0.038	0.114	0.262	Beta
Disutility for Chronic mental health condition	-0.448	0.036	0.377	0.519	Beta
Disutility for Intermittent mental health condition	-0.188	0.038	0.114	0.262	Beta
Disutility for New cases mental health condition	-0.094	0.019	0.057	0.131	Beta
Quality of life gain for high activity	0.020	0.002	0.016	0.024	Beta
**Direct costs**					
Type 2 Diabetes	£622	£62	£500	£744	Gamma
Mental health condition	£1,334	£383	£585	£2,307	Gamma
Mental health condition- chronic	£4,233	£629	£3,001	£7,238	Gamma
Mental health condition- intermittent	£1,334	£383	£585	£2,307	Gamma
Mental health condition-new onset	£667	£191	£292	£1,042	Gamma
Pre-term birth <28 weeks	£25,452	£2,169	£21,201	£29,704	Gamma
Pre-term birth 28-33 weeks	£13,073	£1,114	£10,889	£15,256	Gamma
Pre-term birth, 34-36 weeks	£4,157	£1,020	£2,157	£6,157	Gamma
**Indirect costs**					
Diabetes	£4,116	£412	£3,309	£4,923	Gamma
Mental health condition	£223	£64	£98	£348	Gamma
Mental health condition- chronic	£707	£203	£310	£1,105	Gamma
Mental health condition- intermittent	£223	£64	£98	£348	Gamma
Mental health condition-new onset	£112	£32	£49	£174	Gamma

Table 1 Input parameters used in the model

Parameter	Value	SD/SE	95% CI-Low	95% CI-High	Distribution
Pre-term birth <28 weeks	£397	£114	£174	£619	Gamma
Pre-term birth 28-33 weeks	£119	£34	£52	£185	Gamma
Pre-term birth, 34-36 weeks	£103	£29	£45	£161	Gamma
**Cost of the intervention**	£155	£15	£124	£185	Gamma

SD: Standard Deviation; SE: Standard Error; CI: Confidence Interval

For peer review only

For peer review only - http://bmjopen.bmj.com/site/about/guidelines.xhtml

Table 2 Incremental cost-effectiveness ratios in the base case and scenario analyses					
	**Total cost (£)**	**Total QALYs**	**Incremental cost (£)**	**Incremental QALY**	**ICER**
**Base case analysis**					
Usual schooling	£4,416	13.07			
LifeLab Plus	£4,539	13.08	£123	0.0085	£14,367
**Perspective: NHS**					
Usual schooling	£2,001	13.07			
LifeLab Plus	£2,141	13.08	£140	0.0085	£16,339
**Costs discounted at 3.5%; QALYs at 1.5% per annum**					
Usual schooling	£4,416	15.76			
LifeLab Plus	£4,539	15.77	£123	0.0097	£12,640
**Costs and QALYs discounted at 1.5%**					
Usual schooling	£5,813	15.76			
LifeLab Plus	£5,930	15.77	£117	0.0097	£12,044
**Time Horizon: 5 years**					
Usual schooling	£488	4.25			
LifeLab Plus	£633	4.25	£145	0.0043	£33,423
**Time Horizon: 10 years**					
Usual schooling	£1,132	7.76			
LifeLab Plus	£1,265	7.77	£134	0.0071	£18,851
**Time Horizon: 30 years**					
Usual schooling	£11,137	16.67			
LifeLab Plus	£11,258	16.67	£121	0.0088	£13,719
**Time Horizon: 40 years**					
Usual schooling	£18,417	19.05			
LifeLab Plus	£18,537	19.06	£120	0.0089	£13,455
**Duration of treatment effect: 1 year & Treatment waning: 2 years**					
Usual schooling	£4,416	13.07			
LifeLab Plus	£4,566	13.08	£150	0.0030	£49,758
**Duration of treatment effect: 1 years & Treatment waning: 5 years**					
Usual schooling	£4,416	13.07			
LifeLab Plus	£4,563	13.08	£147	0.0043	£34,089
**Duration of treatment effect: 2 years & Treatment waning: 5 years**					
Usual schooling	£4,416	13.07			
LifeLab Plus	£4,560	13.08	£144	0.0051	£28,034
**Duration of treatment effect: 5 years & Treatment waning: 5 years**					
Usual schooling	£4,416	13.07			
LifeLab Plus	£4,553	13.08	£137	0.0075	£18,218
**Duration of treatment effect: 5 years & Treatment waning: 10 years**					
Usual schooling	£4,416	13.07			
LifeLab Plus	£4,529	13.08	£113	0.0092	£12,184
**Duration of treatment effect: 10 years & Treatment waning: 10 years**					
Usual schooling	£4,416	13.07			
LifeLab Plus	£4,432	13.09	£16	0.0125	£1,257
**Duration of treatment effect: 15 years & Treatment waning: 5 years**					
Usual schooling	£4,416	13.07			

For peer review only

Table 2 Incremental cost-effectiveness ratios in the base case and scenario analyses

	Total cost (£)	Total QALYs	Incremental cost (£)	Incremental QALY	ICER
LifeLab Plus	£4,308	13.09	-£108	0.0147	Dominates

ICER, incremental cost-effectiveness ratio (incremental cost/incremental QALY); QALY, quality-adjusted life year.

For peer review only

For peer review only - http://bmjopen.bmj.com/site/about/guidelines.xhtml

REFERENCES

1. Gc VS, Suhrcke M, Atkin AJ, et al. Cost-effectiveness of physical activity interventions in adolescents: model development and illustration using two exemplar interventions. *BMJ Open* 2019;9(8):e027566. doi: 10.1136/bmjopen-2018-027566 [published Online First: 2019/08/21]
2. Organisation WH. Unhealthy Diets & Physical Inactivity 2009 [Available from: https://www.who.int/nmh/publications/fact_sheet_diet_en.pdf accessed 19/11/2020.
3. Organisation WH. Adolescent Health 2020 [Available from: https://www.who.int/health-topics/adolescent-health/#tab=tab_1 accessed 13/11/20.
4. Viner RM, Ross D, Hardy R, et al. Life course epidemiology: recognising the importance of adolescence. *Journal of Epidemiology and Community Health* 2015;69(8):719-20. doi: 10.1136/jech-2014-205300
5. Rose T, Barker M, Maria Jacob C, et al. A Systematic Review of Digital Interventions for Improving the Diet and Physical Activity Behaviors of Adolescents. *J Adolesc Health* 2017;61(6):669-77. doi: 10.1016/j.jadohealth.2017.05.024 [published Online First: 2017/08/16]
6. Bates B, Lennox A, Prentice A, et al. National Diet and Nutrition Survey: Results from Years 1, 2, 3 and 4 (combined) of the Rolling Programme (2008/2009-2011/2012): a Survey Carried Out on Behalf of Public Health England and the Food Standards Agency: Public Health England, 2014.
7. Woods-Townsend K, Bagust L, Barker M, et al. Engaging teenagers in improving their health behaviours and increasing their interest in science (Evaluation of LifeLab Southampton): study protocol for a cluster randomized controlled trial. *Trials* 2015;16:372-72. doi: 10.1186/s13063-015-0890-z
8. NHS. Health Survey for England 2015 Physical activiy in children 2016 [Available from: http://healthsurvey.hscic.gov.uk/media/37752/hse2015-child-phy-act.pdf accessed 17/11/20.
9. term TNL. 2019 [Available from: https://www.longtermplan.nhs.uk/publication/nhs-long-term-plan accessed 15/09/20.
10. Milner J, Green R, Dangour AD, et al. Health effects of adopting low greenhouse gas emission diets in the UK. *BMJ Open* 2015;5(4):e007364. doi: 10.1136/bmjopen-2014-007364
11. Kelder SH, Perry CL, Klepp KI, et al. Longitudinal tracking of adolescent smoking, physical activity, and food choice behaviors. *Am J Public Health* 1994;84(7):1121-26. doi: 10.2105/ajph.84.7.1121
12. Craigie AM, Lake AA, Kelly SA, et al. Tracking of obesity-related behaviours from childhood to adulthood: A systematic review. *Maturitas* 2011;70(3):266-84. doi: 10.1016/j.maturitas.2011.08.005 [published Online First: 2011/09/17]
13. Hanson M, Gluckman P. Developmental origins of noncommunicable disease: population and public health implications. *Am J Clin Nutr* 2011;94(6 Suppl):1754s-58s. doi: 10.3945/ajcn.110.001206 [published Online First: 2011/04/29]
14. Gates M. Advancing the adolescent health agenda. *The Lancet* 2016;387(10036):2358-59. doi: 10.1016/S0140-6736(16)30298-7
15. Organisation WH. Global Accelerated Action for the Health of Adolescents (AAHA!): guidance to support country implementation. Geneva, 2017.
16. Kelishadi R, Azizi-Soleiman F. Controlling childhood obesity: A systematic review on strategies and challenges. *J Res Med Sci* 2014;19(10):993-1008.
17. Calvert S, Dempsey RC, Povey R. Delivering in-school interventions to improve dietary behaviours amongst 11- to 16-year-olds: A systematic review. *Obesity Reviews* 2019;20(4):543-53. doi: https://doi.org/10.1111/obr.12797

For peer review only - http://bmjopen.bmj.com/site/about/guidelines.xhtml

18. Woods-Townsend K, Hardy-Johnson P, Bagust L, et al. A cluster-randomised controlled trial of the LifeLab education intervention to improve health literacy in adolescents. *PLoS One* in press
19. Ofcom. Children and parents: Media use and attitudes report 2019 [Available from: https://www.ofcom.org.uk/__data/assets/pdf_file/0023/190616/children-media-use-attitudes-2019-report.pdf accessed 10/09/20.
20. Strömmer S, Barrett M, Woods-Townsend K, et al. Engaging adolescents in changing behaviour (EACH-B): a study protocol for a cluster randomised controlled trial to improve dietary quality and physical activity. *Trials* 2020;21(1):859. doi: 10.1186/s13063-020-04761-w
21. Borde R, Smith JJ, Sutherland R, et al. Methodological considerations and impact of school-based interventions on objectively measured physical activity in adolescents: a systematic review and meta-analysis. *Obesity Reviews* 2017;18(4):476-90. doi: https://doi.org/10.1111/obr.12517
22. Buxton MJ, Drummond MF, Van Hout BA, et al. Modelling in Ecomomic Evaluation: An Unavoidable Fact of Life. *Health Economics* 1997;6(3):217-27. doi: https://doi.org/10.1002/(SICI)1099-1050(199705)6:3<217::AID-HEC267>3.0.CO;2-W
23. NHS Digital. Health Survey for England 2016. 2017.
24. Digital N. Health Survey for England 2015 [Available from: https://digital.nhs.uk/data-and-information/publications/statistical/health-survey-for-england/health-survey-for-england-20152020.
25. Sharma M, Nazareth I, Petersen I. Trends in incidence, prevalence and prescribing in type 2 diabetes mellitus between 2000 and 2013 in primary care: a retrospective cohort study. *BMJ Open* 2016;6(1):e010210. doi: 10.1136/bmjopen-2015-010210 [published Online First: 2016/01/16]
26. McManus S BP, Jenkins R, Brugha T. Mental health and wellbeing in England: Adult Psychiatric Morbidity Survey 2014. 2016.
27. NHS Digital. Mental health of children and young people in England, 2017. 2017.
28. Stegenga BT, Kamphuis MH, King M, et al. The natural course and outcome of major depressive disorder in primary care: the PREDICT-NL study. *Soc Psychiatry Psychiatr Epidemiol* 2012;47(1):87-95. doi: 10.1007/s00127-010-0317-9 [published Online First: 2010/11/09]
29. Singhal A, Ross J, Seminog O, et al. Risk of self-harm and suicide in people with specific psychiatric and physical disorders: comparisons between disorders using English national record linkage. *J R Soc Med* 2014;107(5):194-204. doi: 10.1177/0141076814522033 [published Online First: 2014/02/15]
30. Office of National Statistics. Births in England and Wales: 2017. 2018.
31. Gray N, Picone G, Sloan F, et al. Relation between BMI and diabetes mellitus and its complications among US older adults. *South Med J* 2015;108(1):29-36. doi: 10.14423/SMJ.0000000000000214 [published Online First: 2015/01/13]
32. Sutaria S, Devakumar D, Yasuda SS, et al. Is obesity associated with depression in children? Systematic review and meta-analysis. *Arch Dis Child* 2019;104(1):64-74. doi: 10.1136/archdischild-2017-314608 [published Online First: 2018/07/01]
33. McDonald SD, Han Z, Mulla S, et al. Overweight and obesity in mothers and risk of preterm birth and low birth weight infants: systematic review and meta-analyses. *BMJ* 2010;341:c3428. doi: 10.1136/bmj.c3428 [published Online First: 2010/07/22]
34. Schuch FB, Vancampfort D, Firth J, et al. Physical Activity and Incident Depression: A Meta-Analysis of Prospective Cohort Studies. *Am J Psychiatry* 2018;175(7):631-48. doi: 10.1176/appi.ajp.2018.17111194 [published Online First: 2018/04/25]

For peer review only

35. Wahid A, Manek N, Nichols M, et al. Quantifying the Association Between Physical Activity and Cardiovascular Disease and Diabetes: A Systematic Review and Meta‐Analysis. *Journal of the American Heart Association* 2016;5(9) doi: 10.1161/jaha.115.002495
36. Chireh B, Li M, D'Arcy C. Diabetes increases the risk of depression: A systematic review, meta-analysis and estimates of population attributable fractions based on prospective studies. *Prev Med Rep* 2019;14:100822. doi: 10.1016/j.pmedr.2019.100822 [published Online First: 2019/03/01]
37. Liu Z, Xu HM, Wen LM, et al. A systematic review and meta-analysis of the overall effects of school-based obesity prevention interventions and effect differences by intervention components. *Int J Behav Nutr Phys Act* 2019;16(1):95. doi: 10.1186/s12966-019-0848-8 [published Online First: 2019/10/31]
38. Lavelle HV, Mackay DF, Pell JP. Systematic review and meta-analysis of school-based interventions to reduce body mass index. *J Public Health (Oxf)* 2012;34(3):360-9. doi: 10.1093/pubmed/fdr116 [published Online First: 2012/01/24]
39. Jacob CM, Hardy-Johnson PL, Inskip HM, et al. A systematic review and meta-analysis of school-based interventions with health education to reduce body mass index in adolescents aged 10 to 19 years. *Int J Behav Nutr Phys Act* 2021;18(1):1. doi: 10.1186/s12966-020-01065-9 [published Online First: 2021/01/06]
40. Mears R, Jago R. Effectiveness of after-school interventions at increasing moderate-to-vigorous physical activity levels in 5- to 18-year olds: a systematic review and meta-analysis. *Br J Sports Med* 2016;50(21):1315-24. doi: 10.1136/bjsports-2015-094976 [published Online First: 2016/05/26]
41. Statistics OfN. National life tables: England. 2020. https://www.ons.gov.uk/peoplepopulationandcommunity/birthsdeathsandmarriages/lifeexpectancies/datasets/nationallifetablesenglandreferencetables (accessed 22/03/2021).
42. Mulnier HES, H.E.;Raleigh,V.S.;Soedamah-Muthu,S.S.;Colhoun,H.M.;Lawrenson,R.A.;. Mortality in people with Type 2 diabetes in the UK. *Diabetic Medicine* 2006(23):516-21.
43. Maheswaran H, Petrou S, Rees K, et al. Estimating EQ-5D utility values for major health behavioural risk factors in England. *J Epidemiol Community Health* 2013;67(2):172-80. doi: 10.1136/jech-2012-201019 [published Online First: 2012/07/31]
44. Janssen MF, Lubetkin EI, Sekhobo JP, et al. The use of the EQ-5D preference-based health status measure in adults with Type 2 diabetes mellitus. *Diabet Med* 2011;28(4):395-413. doi: 10.1111/j.1464-5491.2010.03136.x [published Online First: 2011/03/12]
45. Astrom M, Persson C, Linden-Bostrom M, et al. Population health status based on the EQ-5D-Y-3L among adolescents in Sweden: Results by sociodemographic factors and self-reported comorbidity. *Qual Life Res* 2018;27(11):2859-71. doi: 10.1007/s11136-018-1985-2 [published Online First: 2018/09/10]
46. Lynch FD, JF.; Feeny, DH.; Clarke, GN.; MacMillan, AL.;. Measuring Health-related Quality of Life in Teens With and Without Depression. *Medical Care* 2016;54(12):8.
47. Morriss R, Garland A, Nixon N, et al. Efficacy and cost-effectiveness of a specialist depression service versus usual specialist mental health care to manage persistent depression: a randomised controlled trial. *The Lancet Psychiatry* 2016;3(9):821-31. doi: 10.1016/s2215-0366(16)30143-2
48. Petrou S, Krabuanrat N, Khan K. Preference-Based Health-Related Quality of Life Outcomes Associated with Preterm Birth: A Systematic Review and Meta-analysis.

For peer review only - http://bmjopen.bmj.com/site/about/guidelines.xhtml

Pharmacoeconomics 2019 doi: 10.1007/s40273-019-00865-7 [published Online First: 2019/12/10]

49. Curtis LB, A.;. Unit costs of Health and Social Care 2018. 2018.
50. Hex N, Bartlett C, Wright D, et al. Estimating the current and future costs of Type 1 and Type 2 diabetes in the UK, including direct health costs and indirect societal and productivity costs. *Diabet Med* 2012;29(7):855-62. doi: 10.1111/j.1464-5491.2012.03698.x [published Online First: 2012/04/28]
51. Kuyken W, Hayes R, Barrett B, et al. Effectiveness and cost-effectiveness of mindfulness-based cognitive therapy compared with maintenance antidepressant treatment in the prevention of depressive relapse or recurrence (PREVENT): a randomised controlled trial. *The Lancet* 2015;386(9988):63-73. doi: 10.1016/s0140-6736(14)62222-4
52. Khan KA, Petrou S, Dritsaki M, et al. Economic costs associated with moderate and late preterm birth: a prospective population-based study. *BJOG* 2015;122(11):1495-505. doi: 10.1111/1471-0528.13515 [published Online First: 2015/07/30]
53. Mangham LJ, Petrou S, Doyle LW, et al. The cost of preterm birth throughout childhood in England and Wales. *Pediatrics* 2009;123(2):e312-27. doi: 10.1542/peds.2008-1827 [published Online First: 2009/01/28]
54. Hamilton D, Dee A, Perry IJ. The lifetime costs of overweight and obesity in childhood and adolescence: a systematic review. *Obes Rev* 2018;19(4):452-63. doi: 10.1111/obr.12649 [published Online First: 2017/12/23]
55. Excellence NIfHaC. Guide to the methods of technology appraisal, 2013.
56. Anderson YC, Leung W, Grant CC, et al. Economic evaluation of a multi-disciplinary community-based intervention programme for New Zealand children and adolescents with obesity. *Obesity Research & Clinical Practice* 2018;12(3):293-98. doi: https://dx.doi.org/10.1016/j.orcp.2018.04.001
57. Ladapo JA, Bogart LM, Klein DJ, et al. Cost and Cost-Effectiveness of Students for Nutrition and eXercise (SNaX). *Academic pediatrics* 2016;16(3):247-53. doi: https://dx.doi.org/10.1016/j.acap.2015.07.009
58. Lee BY, Adam A, Zenkov E, et al. Modeling The Economic And Health Impact Of Increasing Children's Physical Activity In The United States. *Health Affairs* 2017;36(5):902-08. doi: https://dx.doi.org/10.1377/hlthaff.2016.1315
59. Panca M, Christie D, Cole TJ, et al. Cost-effectiveness of a community-delivered multicomponent intervention compared with enhanced standard care of obese adolescents: cost-utility analysis alongside a randomised controlled trial (the HELP trial). *BMJ Open* 2018;8(2):e018640. doi: https://dx.doi.org/10.1136/bmjopen-2017-018640
60. Sutherland R, Reeves P, Campbell E, et al. Cost-effectiveness of a multi-component school-based physical activity intervention targeting adolescents: the 'Physical Activity 4 Everyone' cluster randomized trial. *International Journal of Behavioral Nutrition & Physical Activity* 2016;13:94. doi: https://dx.doi.org/10.1186/s12966-016-0418-2

For peer review only - http://bmjopen.bmj.com/site/about/guidelines.xhtml

Figure 1: Illustration of the conceptual framework and model structure

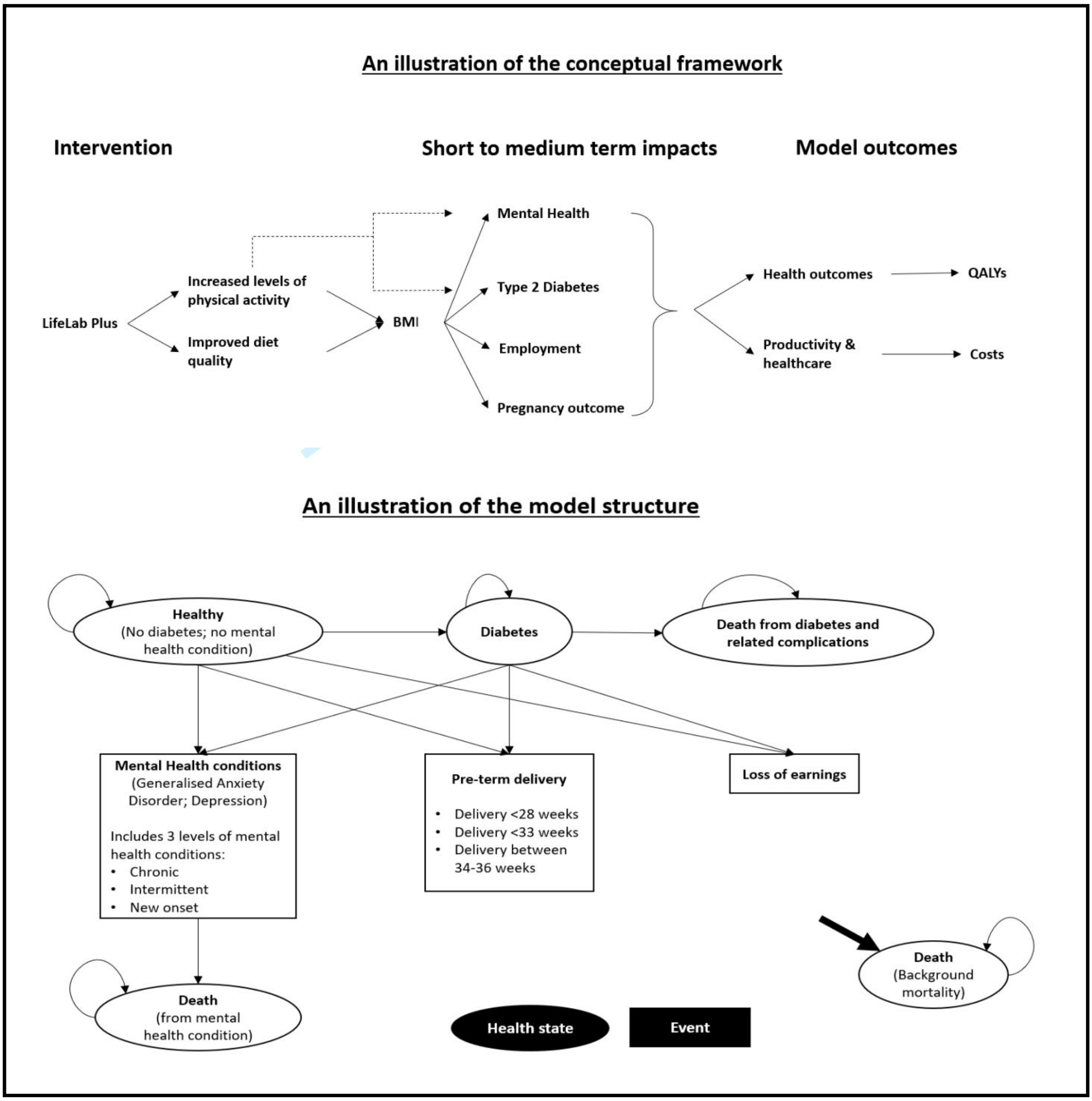

Figure 2: Tornado diagram to show the impact of varying parameter input values on ICERs

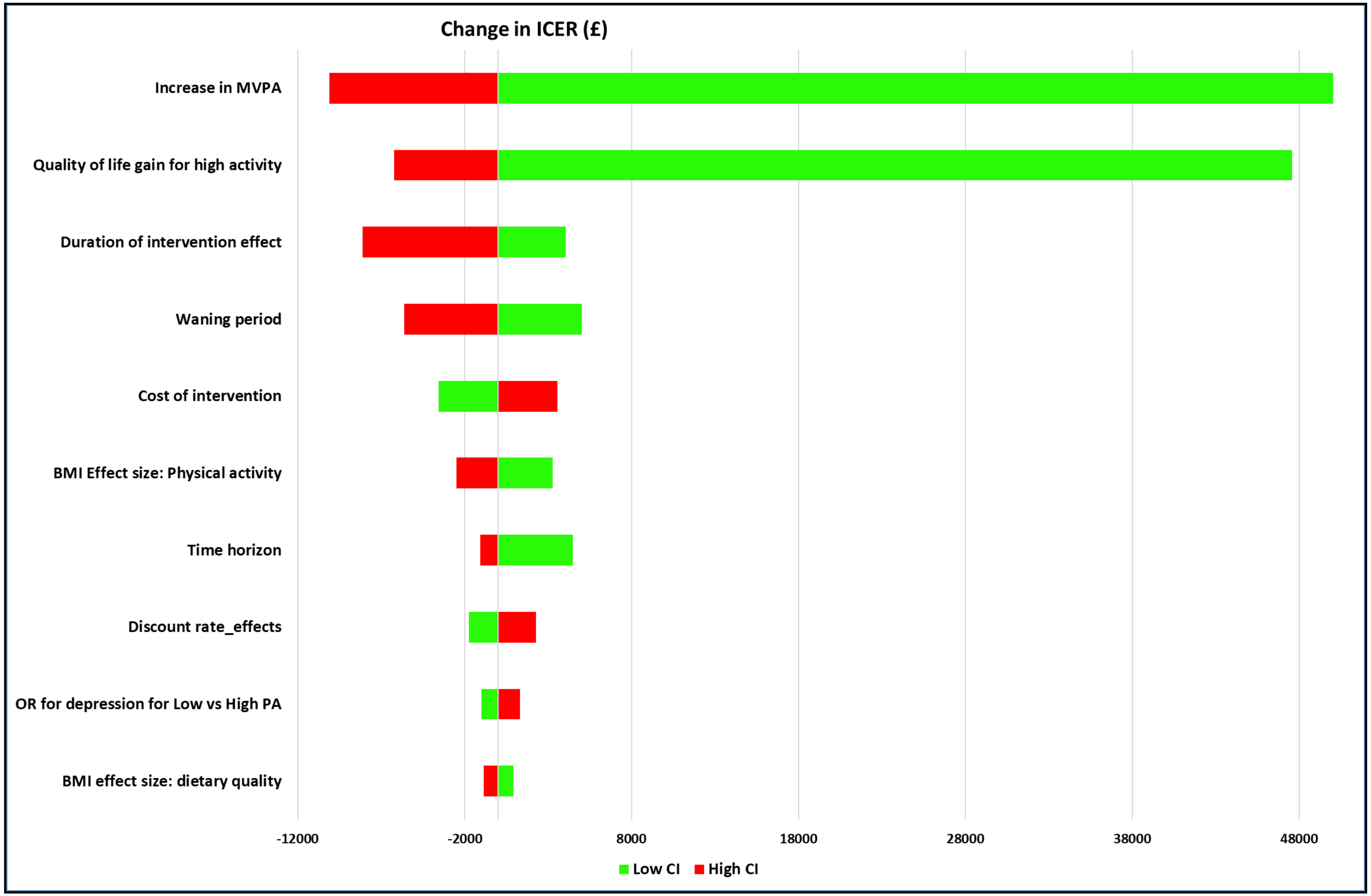

MVPA: Moderate or Vigorous Physical Activity; BMI: Body Mass Index; CI: Confidence Interval; ICER: Incremental Cost Effectiveness Ratio

Figure 3: Scatter plot of PSA cost-effectiveness results

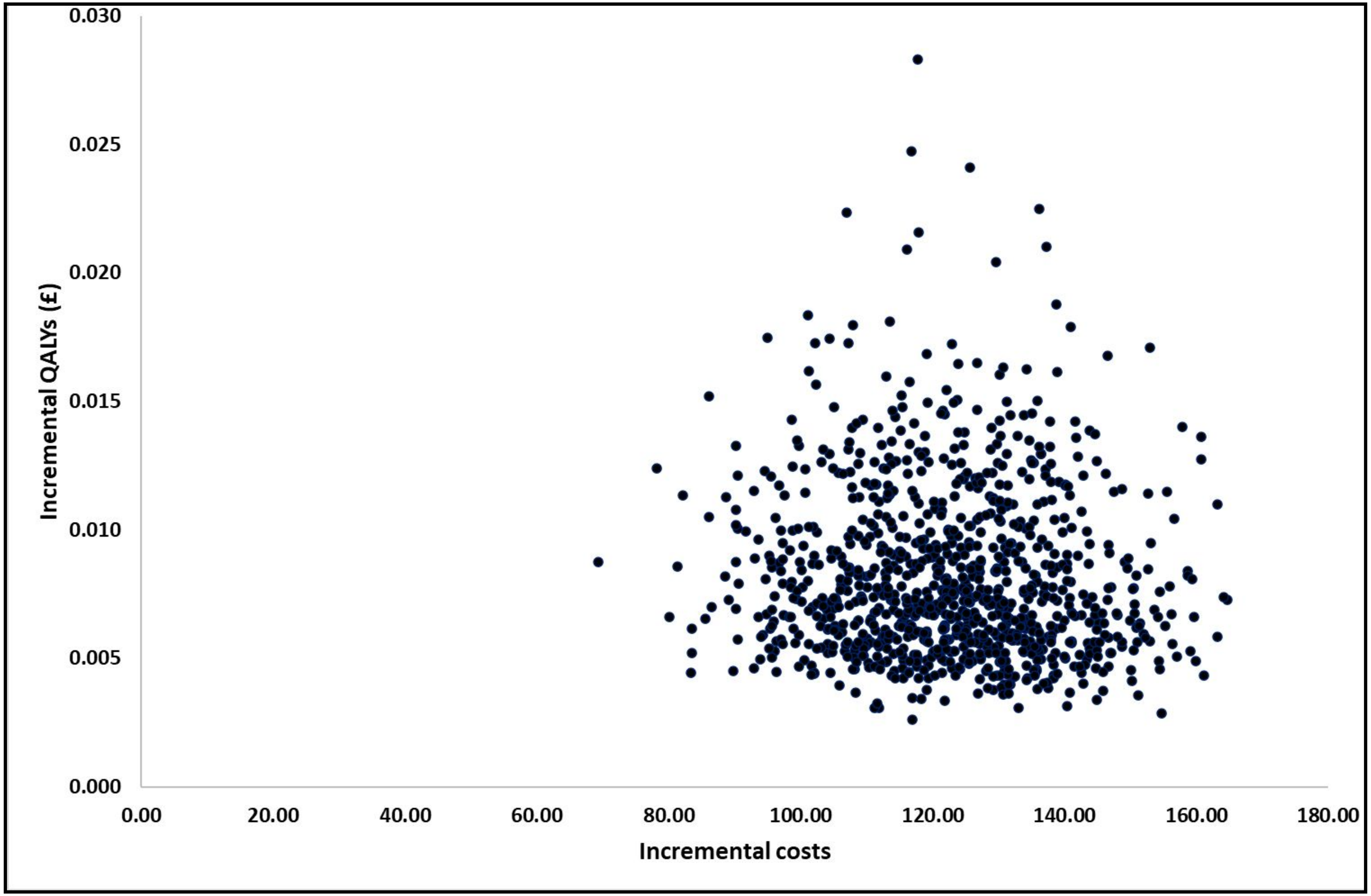

1 APPENDIX A

Model parameters were estimated by using the following equations. For the control arm, rate was based upon age only. For the intervention arm, rate was based on age and the change in BMI from the control population to the intervention.

Table 1: Parameter equations

Parameter	Equation	Units	Source
BMI - men	$-0.0031*age^2+0.4096*age+16.098$	Kg/m2	HSE 2017[1]
BMI - women	$-0.0032*Age^2 + 0.4235*Age+15.812$	Kg/m2	
Type 2 Diabetes Men Control, age <25y	0.025*Age-0.265	Incidence per 1000	Sharma 2015[2]
Type 2 Diabetes Men Control, age >25y	0.0054Age^2-0.191Age+1.7142	Incidence per 1000	
Type 2 Diabetes Women, Control, all ages	0.0031Age^2-0.0942Age+1.2045	Incidence per 1000	
Type 2 Diabetes Men, Intervention, age < 25y	(0.025*Age-0.265)/(0.081BMI_control-0.8451)*(0.081BMI_int-0.8451)	Incidence per 1000	Gray et al 2015[3]
Type 2 Diabetes Men, Intervention, > 25y	(0.0054Age^2-0.191Age+1.7142)/(0.081BMI_control-0.8451)*(0.081BMI_int-0.8451)	Incidence per 1000	
Type 2 Diabetes Women Intervention, all ages	(0.0031Age^2-0.0942Age+1.2045)/(0.0637BMI_control -0.3135)*(0.0637BMI_Int-0.3135)	Incidence per 1000	
Mental health Men, control	-0.00008*Age^2+0.0073*Age-0.0544	% Incidence	APMS 2014[4]
Mental health Women, control	-0.00007*Age^2+0.0062*Age+0.0034	% Incidence	
Mental Health Men, intervention	(-0.00007*Age^2+0.0062*Age+0.0034)*(0.0127*BMI_I+0.727)/(0.0127*BMI_C +0.727)	% Incidence	Sutaria et al 2018[5]
Mental Health Women, intervention	(-0.00007*Age^2+0.0062*Age+0.0034) *(0.0391*BMI_I+0.1087)/(0.0391*BMI_C+0.1087)	% Incidence	
Mental Health, diabetes	Multiply MH risk by 1.33		Chireh et al 2019[6]
Mental Health, Physical exercise	OR 0.83 high activity vs low activity. Applied to intervention arm only.		Schuch et al 2018[7]
Preterm birth <33 weeks, control	$-0.0512*Age^2+3.0922*Age-37.891$	Incidence per 1000	ONS 2017[8]
Preterm birth <33 weeks, intervention	(-0.0512*Age2+3.0922*Age-37.891)*(0.0514*BMI_I-0.1712)/(0.0514*BMI_C-0.1712)	Incidence per 1000	Mcdonald et al 2010[9]
Loss of earnings, men	12534BMI – 297703	£ over lifetime	Hamilton et al

For peer review only - http://bmjopen.bmj.com/site/about/guidelines.xhtml

Parameter	Equation	Units	Source
Loss of earning, women	17695BMI - 439796	£ over lifetime	2018[10]

Table 2: Parameter equations for z-scores

Parameter	Equation	Units	Source
Mental Health Men, Intervention	(0.0054Age^2-0.191Age+1.7142)/(0.0274BMI_z_score_control+1.0011)*(0.0274BMI_z_scores_int+1.011)	Incidence per 1000	Gray et al 2015[3]
Mental Health Women, Intervention	(0.0031Age^2-0.0942Age+1.2045)/(0.084BMI_z_scores_control+ 0.9486)*(0.084BMI_z_scores_Int+0.9486)	Incidence per 1000	
Type 2 Diabetes Men, intervention	(-0.00007*Age^2+0.0062*Age+0.0034)/(0.1742BMI_z_scores_control+ 0.8964)*(0.1742BMI_z_scores_Int+0.8964)	% Incidence	Sutaria et al 2018[5]
Type 2 Diabetes Women, intervention	(-0.00007*Age^2+0.0062*Age+0.0034)*(0.1369*BMI_z_scores_I+1.0556)/(0.1369*BMI_z_scores_C+1.0556)	% Incidence	
Preterm birth <33 weeks, intervention	(-0.0512*Age2+3.0922*Age-37.891)*(0.1105*BMI_z_scores_I+0.9336)/(0.1105*BMI_z_scores_C+0.9336)	Incidence per 1000	Mcdonald et al 2010[9]

Table 3: Parameter equation for quality of life estimates

Parameter	Equation	Source
Quality of life, by age	0.9508566+0.0212126*Male-0.0002587*Age-0.0000332*Age^2	Maheswaran et al 2012[11]
Disutility for BMI, if BMI < 30	-0.0009BMI + 0.0189	
Disutility for BMI, if BMI > 30	-0.0036BMI+0.094	

For peer review only

For peer review only - http://bmjopen.bmj.com/site/about/guidelines.xhtml

Table 4: Input parameters used in the model

Parameter	Value	SD/SE	95% CI-Low	95% CI-High	Distribution
**Direct trial efficacy**					
BMI effect size: Physical activity	0.13	0.046	0.040	0.220	Beta
BMI effect size: Dietary quality	0.04	0.01423	0.012	0.068	Beta
BMI z-scores effect size: Physical activity	0.04	0.02	0.001	0.079	Beta
BMI z-scores effect size: Dietary quality	0.02	0.01	0.000	0.040	Beta
Prop. meeting PA guidelines control	0.13	0.0013	0.127	0.133	Beta
Prop. meeting PA guidelines intervention	0.16	0.049	0.117	0.257	Beta
Increase in Moderate to Vigorous PA	4.84	2.944	-0.940	10.610	Beta
**Relative effectiveness**					
OR for depression for Low vs High Physical Activity	0.83	0.026	0.790	0.880	Beta
OR of diabetes increasing the risk of depression	1.33	0.133	1.069	1.591	Lognormal
RR of PA on incidence of diabetes	0.74	0.074	0.595	0.885	Lognormal
**Mortality**					
Mental health: risk of death by suicide	0.001	0.000	0.001	0.001	Beta
HR of mortality those with type 2 Diabetes aged <65 years	2.978	0.190	2.605	3.350	Lognormal
HR of mortality those with type 2 Diabetes aged >65 years	1.958	0.050	1.860	2.056	Lognormal
**Relative effectiveness[a]**					
Pre-term birth relative risk for BMI P1	0.051	0.005	0.041	0.061	Beta
Pre-term birth relative risk for BMI P2	-0.171		0.111	-0.322	
MH BMI relative risk Boys P1	0.013	0.004	0.004	0.021	Beta
MH BMI relative risk Boys P2	0.727		0.914	0.540	
MH BMI relative risk girls P1	0.039	0.004	0.031	0.047	Beta
MH BMI relative risk girls P2	0.109		0.324	-0.005	
Diabetes relative risk boys P1	0.081	0.004	0.073	0.089	Beta
Diabetes relative risk boys P2	-0.845		-0.571	-0.912	
Diabetes relative risk girls P1	0.064	0.013	0.039	0.089	Beta

For peer review only - http://bmjopen.bmj.com/site/about/guidelines.xhtml

Parameter	Value	SD/SE	95% CI-Low	95% CI-High	Distribution
Diabetes relative risk girls P2	-0.314		0.167	-0.906	
Loss of earnings Men P1	12036	601.8	10856	13215.5	Gamma
Loss of earnings Men P2	-284478		-256213	-311886.5	
Loss of earnings Women P1	16689	1668.9	13418	19960.0	Gamma
Loss of earnings Women P2	-412424		-339474	-504989.1	
**Z scores [a]**					
Pre-term birth relative risk for BMI z-scores P1	0.1105	0.011	0.089	0.132	Beta
Pre-term birth relative risk for BMI z-scores P2	0.9336		-0.910	-1.841	
MH BMI z-scores relative risk Boys P1	0.0274	0.003	0.022	0.033	Beta
MH BMI z-scores relative risk Boys P2	1.0011		0.526	0.295	
MH BMI z-scores relative risk girls P1	0.084	0.008	0.068	0.100	Beta
MH BMI z-scores relative risk girls P2	0.9486		-0.452	-1.160	
Diabetes relative risk boys P1	0.1742	0.009	0.157	0.191	Beta
Diabetes relative risk boys P2	0.8964		-2.378	-3.112	
Diabetes relative risk girls P1	0.1369	0.027	0.083	0.191	Beta
Diabetes relative risk girls P2	1.0556		-0.790	-3.097	
**Proportions of population with mental health conditions**					
Chronic	0.170	0.017	0.137	0.203	Dirichlet
intermittent	0.400	0.040	0.322	0.478	Dirichlet
New and one-off	0.430	0.043	0.346	0.514	Dirichlet

[a] Risk equation is in the form: Relative risk = P1*BMI + P2

For peer review only - http://bmjopen.bmj.com/site/about/guidelines.xhtml

2 APPENDIX B

A breakdown of the resources used for estimating the costs of the intervention i.e. LifeLab Plus is presented below. The intervention comprises of three components:

i. Life Lab
ii. Digital App
iii. Teachers' Support (Healthy Conversation skills)

The costs are estimated for one-year that is, for the trial duration. The assumptions related to resources-used are based on a discussion with KTW on 5th June 2019.

Table 5: Life Lab

Item	Number	Comment
Number of classes per year	50	
No of pupils in each class	25	Varies between 25-32; assumed 25 for the base case
Total pupils	1250	=50 classes x 25 pupils per class
No of schoolteachers in each class	1	
Total teachers	50	

Table 6: Staff Training for Life Lab and Healthy Conversation Skills

Item	Number	Comment
Lifelab staff / training day (days)	6	Each training day / Lifelab day consists of 6 staff days to run. 4 staff days to run training and preparation and 2 days of administration.
Teacher training		
No. of Training days	5	Each training day is for 10 teachers.
Lifelab Staff days	30	=5 training days x 6 staff days / training day
Lifelab		
Number of days	50	=50x1
Lifelab Staff days	300	=50 life lab days x 6 staff day / lifelab
Scientists		Each lifelab day consists of 3 scientists
Number trained	75	Assumed each scientist does two lifelab days
Training days	3	=75 scientists /25; 25 trained per day
Lifelab Staff days	18	=3 training days x 6 staff days / training day
Training the trainers (Healthy Conversation Skills)		
Trainers	6	Assumption
Lifelab Staff days	1	Assume takes 2 hours
Showcase		All schools
Lifelab Staff days	3.5	1.5 days prep; 1 day delivery for 2 staff
Total lifelab staff days	352.5	Band 4 teacher

For peer review only - http://bmjopen.bmj.com/site/about/guidelines.xhtml

Data collection – Lifelab
Each class has two hours of data collection by two Lifelab staffs, 2 research nurses and 1 research assistant.

Table 7: Costs for data collection

Item	Number	Comment
Lifelab staff days	28.6	=2 hours x2 staff x50 (lifelab days)/7 (hours)
Research nurse	28.6	=2 hours x2 staff x50 (lifelab days)/7 (hours)
Research assistant	14.3	=2 hours x1 staff x50 (lifelab days)/7 (hours)

Table 8: Staff unit costs

Staff costs	Salary	Comment
Band 4 teacher	£34,189	i.e. £163 per day, Assumes 210 working days per year
Research assistant	£27,025	i.e. £129 per day, Assumes 210 working days per year
Research nurse Band 5	£26,231	i.e £125 per day, Assumes 210 working days per year

Table 9: Staff total costs

Staff costs	Total	Comment
Lifelab / training	£71,736	=£34,189/210x352.5*1.25;<br>= salary / working days per year*lifelab staff days*NI and pension adjustment<br>Assumes 210 working days per year, pension / NI contributions of 25%
Follow-up data collection	£12,641	=(£34,189*28.6 + £27,025 *28.6 + 26,231 *14.3)/210*1.25<br>= (salary teacher* data collection days teacher + salary research nurse* data collection days research nurse + salary research assistant*data collection days research assistant)/working days per year*NI / pension adjustment
Total	£84,377	

Table 10: Digital App development

Item	Total	Comment
Development of app	£324,000	Estimate from expert
Duration of development, year	5	As above
Duration of app, years	10	Assumption
Number of centres using app	10	Assumption
Cost per year development	£64,800	£324,000 / 5; total cost / number of years of development
Maintenance cost	£16,200	Assumed to be 25% of development cost
Cost per year of development across app lifetime per centre	£3240	£324,000 / 100; total development cost / (number of years app lasts for x number of centres)
EACH-B development for new centre	£87,445	Assumed to be 1 year of staff costs.

For peer review only - http://bmjopen.bmj.com/site/about/guidelines.xhtml

Item	Total	Comment
Number of years centre lasts	10	Assumption
Cost per year of centre	£8745	£87,445 / 10

Table 11: Summary of included costs

Item	Total	Comment
Staff costs	£84,377	
Room hire	£26,857	=400 (room hire cost)*67.14 (number of days); assumes cost of hire is £400 / day
Consumables	£15,839	Consumables include costs associated with printing, paper, classroom
Overheads	£37,970	Direct overheads based on 29% of direct care salary costs and indirect overheads based on 16% direct care salary costs (As used in Curtis et al 2019 for overheads for healthcare professionals)
Maintenance of app	£16,200	See above table
Capital cost, development of app	£3240	See above
Capital cost, development of EACH-B	£8745	See above

Table 12: Total costs

Item	Total	Comment
Total cost	£193,228	Sum of all of the above costs
Cost per pupil	£154.58	=£193,228/1250; (total cost /total number of pupils)

For peer review only

For peer review only - http://bmjopen.bmj.com/site/about/guidelines.xhtml

3 APPENDIX C

Figure 1: Cost-Effectiveness Acceptability Curve (CEAC)

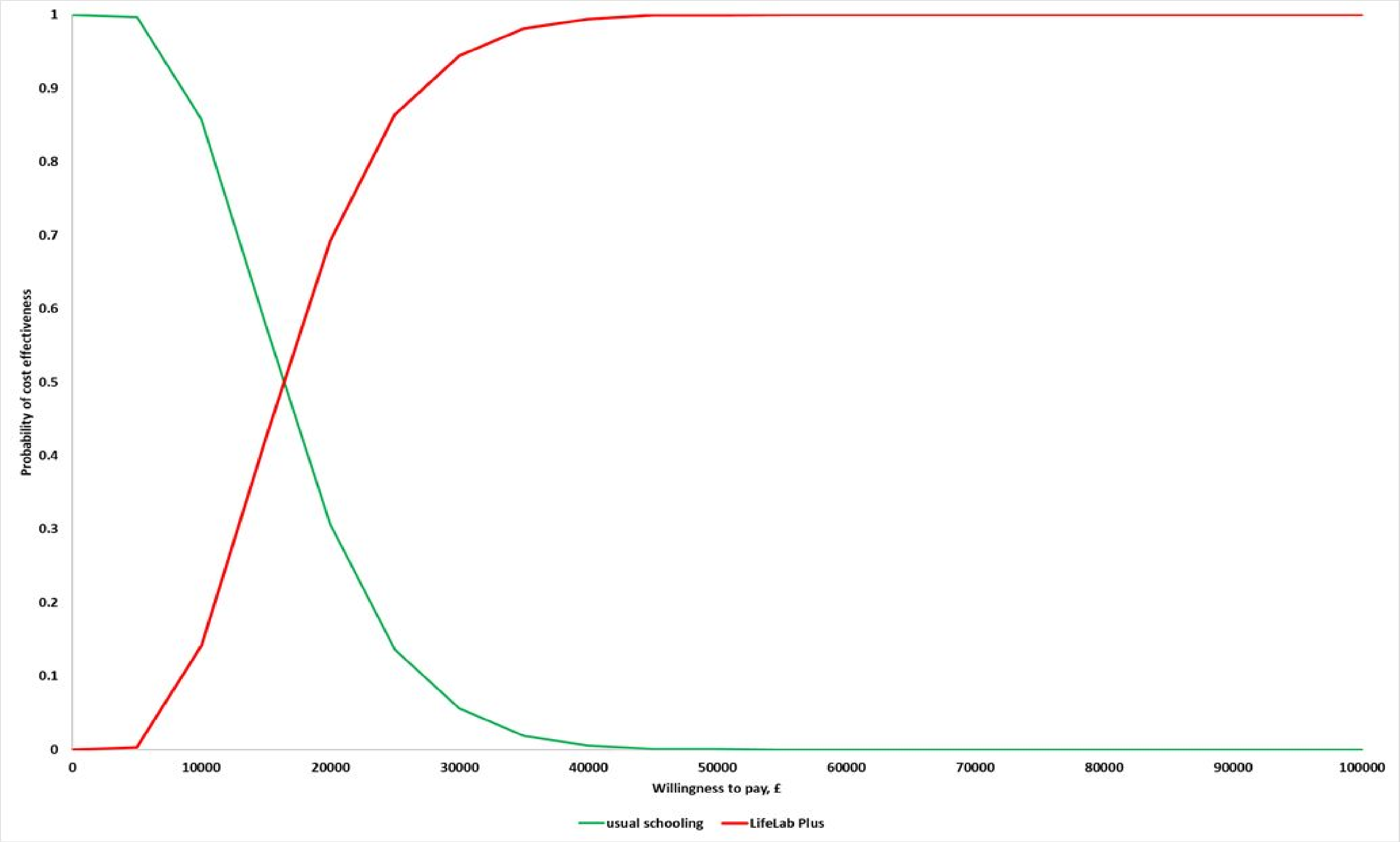

For peer review only - http://bmjopen.bmj.com/site/about/guidelines.xhtml

REFERENCES

1. NHS Digital. Health Survey for England 2016. 2017.
2. Sharma M, Nazareth I, Petersen I. Trends in incidence, prevalence and prescribing in type 2 diabetes mellitus between 2000 and 2013 in primary care: a retrospective cohort study. *BMJ Open* 2016;6(1):e010210. doi: 10.1136/bmjopen-2015-010210 [published Online First: 2016/01/16]
3. Gray N, Picone G, Sloan F, et al. Relation between BMI and diabetes mellitus and its complications among US older adults. *South Med J* 2015;108(1):29-36. doi: 10.14423/SMJ.0000000000000214 [published Online First: 2015/01/13]
4. McManus S BP, Jenkins R, Brugha T. Mental health and wellbeing in England: Adult Psychiatric Morbidity Survey 2014. 2016.
5. Sutaria S, Devakumar D, Yasuda SS, et al. Is obesity associated with depression in children? Systematic review and meta-analysis. *Arch Dis Child* 2019;104(1):64-74. doi: 10.1136/archdischild-2017-314608 [published Online First: 2018/07/01]
6. Chireh B, Li M, D'Arcy C. Diabetes increases the risk of depression: A systematic review, meta-analysis and estimates of population attributable fractions based on prospective studies. *Prev Med Rep* 2019;14:100822. doi: 10.1016/j.pmedr.2019.100822 [published Online First: 2019/03/01]
7. Schuch FB, Vancampfort D, Richards J, et al. Exercise as a treatment for depression: A meta-analysis adjusting for publication bias. *J Psychiatr Res* 2016;77:42-51. doi: 10.1016/j.jpsychires.2016.02.023 [published Online First: 2016/03/16]
8. Office of National Statistics. Births in England and Wales: 2017. 2018.
9. McDonald SD, Han Z, Mulla S, et al. Overweight and obesity in mothers and risk of preterm birth and low birth weight infants: systematic review and meta-analyses. *BMJ* 2010;341:c3428. doi: 10.1136/bmj.c3428 [published Online First: 2010/07/22]
10. Hamilton D, Dee A, Perry IJ. The lifetime costs of overweight and obesity in childhood and adolescence: a systematic review. *Obes Rev* 2018;19(4):452-63. doi: 10.1111/obr.12649 [published Online First: 2017/12/23]
11. Maheswaran H, Petrou S, Rees K, et al. Estimating EQ-5D utility values for major health behavioural risk factors in England. *J Epidemiol Community Health* 2013;67(2):172-80. doi: 10.1136/jech-2012-201019 [published Online First: 2012/07/31]

For peer review only - http://bmjopen.bmj.com/site/about/guidelines.xhtml

CHEERS checklist—Items to include when reporting economic evaluations of health interventions

Section/item	Item No	Recommendation	Reported on page No/ line No
**Title and abstract**			
Title	1	Identify the study as an economic evaluation or use more specific terms such as "cost-effectiveness analysis", and describe the interventions compared.	page 1, line 2
Abstract	2	Provide a structured summary of objectives, perspective, setting, methods (including study design and inputs), results (including base case and uncertainty analyses), and conclusions.	page 2, line 2 to 25
**Introduction**			
Background and objectives	3	Provide an explicit statement of the broader context for the study.	page 4, line 17 to page 5, line 12
		Present the study question and its relevance for health policy or practice decisions.	page 5, line 14 to 29
**Methods**			
Target population and subgroups	4	Describe characteristics of the base case population and subgroups analysed, including why they were chosen.	page 6, line 17 to 21
Setting and location	5	State relevant aspects of the system(s) in which the decision(s) need(s) to be made.	page 4, line 30 to page 5, line 3; page 6, line 19 to 20
Study perspective	6	Describe the perspective of the study and relate this to the costs being evaluated.	page 11, line 19 to 21; page 14, line 7;
Comparators	7	Describe the interventions or strategies being compared and state why they were chosen.	page 5, line 16 to 22; page 6, line 19 to 21
Time horizon	8	State the time horizon(s) over which costs and consequences are being evaluated and say why appropriate.	page 5, line 26 to 28; page 7, line 18 to 22
Discount rate	9	Report the choice of discount rate(s) used for costs and outcomes and say why appropriate.	page 11, line 19 to 22
Choice of health outcomes	10	Describe what outcomes were used as the measure(s) of benefit in the evaluation and their relevance for the type of analysis performed.	page 9, line 22 to page 10, line 5; page 16, Table 1
Measurement of effectiveness	11a	*Single study-based estimates:* Describe fully the design features of the single effectiveness study and why the single study was a sufficient source of clinical effectiveness data.	not applicable
	11b	*Synthesis-based estimates*: Describe fully the methods used for identification of included studies and synthesis of clinical effectiveness data.	page 7, line 24 to page 9, line 20; supplement appendix A
Measurement and valuation of preference based outcomes	12	If applicable, describe the population and methods used to elicit preferences for outcomes.	not applicable
Estimating resources and costs	13a	*Single study-based economic evaluation:*Describe approaches used to estimate resource use associated with the alternative interventions. Describe primary or secondary research methods for valuing each resource item in terms of its unit cost. Describe any adjustments made to approximate to opportunity costs.	not applicable
	13b	*Model-based economic evaluation:* Describe	page 10, line 7 to

For peer review only

Section/item	Item No	Recommendation	Reported on page No/ line No
		approaches and data sources used to estimate resource use associated with model health states. Describe primary or secondary research methods for valuing each resource item in terms of its unit cost. Describe any adjustments made to approximate to opportunity costs.	page 11, line 5; page 16-17, table 1; supplement Appendix B
Currency, price date, and conversion	14	Report the dates of the estimated resource quantities and unit costs. Describe methods for adjusting estimated unit costs to the year of reported costs if necessary. Describe methods for converting costs into a common currency base and the exchange rate.	page 10, line 23 to 24; page 11, line 8 to 9; page 16, table 1
Choice of model	15	Describe and give reasons for the specific type of decision-analytical model used. Providing a figure to show model structure is strongly recommended.	page 5, line 32 to page 6, line 4; figure 1.
Assumptions	16	Describe all structural or other assumptions underpinning the decision-analytical model.	Page 5, line 26 to 28; page 6, line 6 to 14; page 7, line 16 to 23; page 8, line 9 to 17; page 8, line 20 to 21; page 8, line 28 to 29; page 8, line 33 to page 9, line 5; page 9, line 13 to 15; page 9, line 25 to 27; page 9, line 29 to 34; page 10, line 4 to 5; page 10, line 10 to 14; page 10, line 28 to 29; page 11, line 3 to 5; page 12, line 2 to 3; page 13, line 12 to 15; page 14, line 23 to 28
Analytical methods	17	Describe all analytical methods supporting the evaluation. This could include methods for dealing with skewed, missing, or censored data; extrapolation methods; methods for pooling data; approaches to validate or make adjustments (such as half cycle corrections) to a model; and methods for handling population heterogeneity and uncertainty.	Page 11, line 12 to 16; page 11, line 31 to page12, line 9; page 16 to 17, table 1; Supplement Appendix A, Table 4
**Results**			
Study parameters	18	Report the values, ranges, references, and, if used, probability distributions for all parameters. Report reasons or sources for distributions used to represent uncertainty where appropriate. Providing a table to show the input values is strongly recommended.	Page 7, line16 to page 9, line 20; Page 12, line 7 to 8; Page 16 to 17, table 1; supplement Appendix A, Table 1 to 4.
Incremental costs and outcomes	19	For each intervention, report mean values for the main categories of estimated costs and outcomes of interest, as well as mean differences between the comparator groups. If applicable, report incremental cost-effectiveness ratios.	page 12, line 18 to 23; page 18 to 19, table 2
Characterising uncertainty	20a	*Single study-based economic evaluation:*Describe the effects of sampling uncertainty for the estimated incremental cost and incremental effectiveness parameters, together with the impact of	not applicable

For peer review only

Section/item	Item No	Recommendation	Reported on page No/ line No
		methodological assumptions (such as discount rate, study perspective).	
	20b	*Model-based economic evaluation:* Describe the effects on the results of uncertainty for all input parameters, and uncertainty related to the structure of the model and assumptions.	page 12, line 26 to page 13, line 5; figures 2 and 3; page 8 to 9, table 2; supplement Appendix C figure 1
Characterising heterogeneity	21	If applicable, report differences in costs, outcomes, or cost-effectiveness that can be explained by variations between subgroups of patients with different baseline characteristics or other observed variability in effects that are not reducible by more information.	not applicable
**Discussion**			
Study findings, limitations, generalisability, and current knowledge	22	Summarise key study findings and describe how they support the conclusions reached. Discuss limitations and the generalisability of the findings and how the findings fit with current knowledge.	page 13, line 8 to page 14, line 33
**Other**			
Source of funding	23	Describe how the study was funded and the role of the funder in the identification, design, conduct, and reporting of the analysis. Describe other non-monetary sources of support.	page 15, line 18 to 24
Conflicts of interest	24	Describe any potential for conflict of interest of study contributors in accordance with journal policy. In the absence of a journal policy, we recommend authors comply with International Committee of Medical Journal Editors recommendations.	page 15, line 26 to 29

For consistency, the CHEERS statement checklist format is based on the format of the CONSORT statement checklist

For peer review only

BMJ Open

Cost-effectiveness of a dietary and physical activity intervention in adolescents: a modelling study based on the Engaging Adolescents in Changing Behaviour (EACH-B) programme

Journal:	*BMJ Open*
Manuscript ID	bmjopen-2021-052611.R1
Article Type:	Original research
Date Submitted by the Author:	01-Mar-2022
Complete List of Authors:	Kalita, Neelam; University of Southampton, Southampton Health Technology Assessments Centre<br>Cooper, Keith; Univ Southampton, SHTAC<br>Baird, Janis; University of Southampton, MRC Epidemiology Resource Centre; University of Southampton, NIHR Southampton Biomedical Research Centre<br>Woods-Townsend, Kathryn ; University of Southampton, Southampton Education School, Faculty of Social Sciences; University of Southampton , NIHR Southampton Biomedical Research Centre<br>Godfrey, Keith; University of Southampton and Southampton University Hospitals NHS Trust, MRC Lifecourse Epidemiology Unit; NIHR Southampton Biomedical Research Centre<br>Cooper, Cyrus; University of Southampton and Southampton University Hospitals NHS Trust, MRC Lifecourse Epidemiology Unit; University of Oxford, Nuffield Department of Orthopaedics, Rheumatology and Musculoskeletal Sciences<br>Inskip, Hazel; Medical Research Council, Epidemiology Resource Centre; NIHR Southampton Biomedical Research Centre<br>Barker, Mary; University of Southampton, MRC Lifecourse Epidemiology Unit; NIHR Southampton Biomedical Research Centre<br>Lord, Joanne; University of Southampton, Southampton Health Technology Assessments Centre
**Primary Subject Heading**:	Health economics
Secondary Subject Heading:	Diabetes and endocrinology, Health economics, Mental health, Nutrition and metabolism, Paediatrics
Keywords:	HEALTH ECONOMICS, MENTAL HEALTH, NUTRITION & DIETETICS, General diabetes < DIABETES & ENDOCRINOLOGY, Public health < INFECTIOUS DISEASES

For peer review only - http://bmjopen.bmj.com/site/about/guidelines.xhtml

I, the Submitting Author has the right to grant and does grant on behalf of all authors of the Work (as defined in the below author licence), an exclusive licence and/or a non-exclusive licence for contributions from authors who are: i) UK Crown employees; ii) where BMJ has agreed a CC-BY licence shall apply, and/or iii) in accordance with the terms applicable for US Federal Government officers or employees acting as part of their official duties; on a worldwide, perpetual, irrevocable, royalty-free basis to BMJ Publishing Group Ltd ("BMJ") its licensees and where the relevant Journal is co-owned by BMJ to the co-owners of the Journal, to publish the Work in this journal and any other BMJ products and to exploit all rights, as set out in our licence.

The Submitting Author accepts and understands that any supply made under these terms is made by BMJ to the Submitting Author unless you are acting as an employee on behalf of your employer or a postgraduate student of an affiliated institution which is paying any applicable article publishing charge ("APC") for Open Access articles. Where the Submitting Author wishes to make the Work available on an Open Access basis (and intends to pay the relevant APC), the terms of reuse of such Open Access shall be governed by a Creative Commons licence – details of these licences and which Creative Commons licence will apply to this Work are set out in our licence referred to above.

Other than as permitted in any relevant BMJ Author's Self Archiving Policies, I confirm this Work has not been accepted for publication elsewhere, is not being considered for publication elsewhere and does not duplicate material already published. I confirm all authors consent to publication of this Work and authorise the granting of this licence.

For peer review only

Cost-effectiveness of a dietary and physical activity intervention in adolescents: a modelling study based on the Engaging Adolescents in Changing Behaviour (EACH-B) programme

Authors: Neelam Kalita[1], Keith Cooper[1], Janis Baird[2;3], Kathryn Woods-Townsend[3;4], Keith M Godfrey[2;3], Cyrus Cooper[2;6;7], Hazel Inskip[2;3], Mary Barker[2;3;5], Joanne Lord[1] and the EACH-B study group

Affiliations:

[1] Southampton Health Technology Assessments Centre, University of Southampton, UK

[2] MRC Lifecourse Epidemiology Unit, University of Southampton, Southampton, UK

[3] NIHR Southampton Biomedical Research Centre, University of Southampton and University Hospital Southampton NHS Foundation Trust, Southampton, UK

[4] Southampton Education School, Faculty of Social Sciences, University of Southampton, Southampton, UK

[5] School of Health Sciences, University of Southampton, Southampton, UK

[6] Faculty of Medicine, University of Southampton

[7] Nuffield Department of Orthopaedics, Rheumatology and Musculoskeletal Sciences, University of Oxford

Corresponding author: Neelam Kalita

Email: N.Kalita@soton.ac.uk

For peer review only

ABSTRACT

Objective To assess costs, health outcomes and cost-effectiveness of interventions that aim to improve quality of diet and level of physical activity in adolescents.

Design A Markov model was developed to assess four potential benefits of healthy behaviour for adolescents: better mental health (episodes of depression and generalised anxiety disorder), higher earnings and reduced incidence of Type 2 diabetes and adverse pregnancy outcomes (in terms of pre-term delivery). The model parameters were informed by published literature. The analysis took a societal perspective over a 20-year period. One-way and probabilistic sensitivity analyses for 10,000 simulations were conducted.

Participants A hypothetical cohort of 100 adolescents with a mean age of 13 years.

Interventions An exemplar school-based, multi-component intervention that was developed by the Engaging Adolescents for Changing Behaviour programme, compared with usual schooling.

Outcome measure Incremental cost-effectiveness ratio as measured by cost per quality-adjusted life year (QALY) gained.

Results The exemplar dietary and physical activity intervention was associated with an incremental cost of £123 per adolescent and better health outcomes with a mean QALY gain of 0.0085 compared with usual schooling, resulting in an ICER of £14,367 per QALY. The results suggested that such an intervention has the potential to offer a cost-effective use of healthcare-resources for adolescents in the UK at a willingness-to-pay threshold of £20,000 per QALY. The key model drivers are the intervention effect on levels of physical activity, quality-of-life gain for high levels of physical activity, the duration of the intervention effects and the period over which effects wane.

Conclusions The model focused on short to medium-term benefits of healthy eating and physical activity exploiting the strong evidence base that exists for this age group. Other benefits in later life, such as reduced cardiovascular risk, are more sensitive to assumptions about the persistence of behavioural change and discounting.

For peer review only - http://bmjopen.bmj.com/site/about/guidelines.xhtml

ARTICLE SUMMARY

Strengths and limitations of this study

- The model was developed, based on currently available evidence, to assess the costs and health benefits of an exemplar school-based, multi-component intervention to improve adolescents' diet and increase their levels of physical activity.
- The economic evaluation followed published best-practice guidance.
- In the absence of effectiveness results from the on-going EACH-B trial, the treatment effect of the intervention is obtained from a published systematic review.
- The model does not include long-term health outcomes, such as for cardiovascular diseases or different types of cancer.
- There remains uncertainty around key model assumptions related to the duration of benefits of the intervention.

Mesh terms: Adolescent, Cost-effectiveness, School-based, Exercise, Diet

For peer review only - http://bmjopen.bmj.com/site/about/guidelines.xhtml

INTRODUCTION

Poor diet and lack of physical activity increase the risk of non-communicable diseases (NCDs), including cardiovascular diseases, Type 2 diabetes and some cancers such as breast, colon and endometrial, in part by contributing to overweight and obesity.[1 2] Adolescence, the life stage between childhood and adulthood, is a critical period for the development of health and disease in later life.[3 4] Compared with other age-groups, adolescents have the unhealthiest diets and most (over 80%) fail to meet national guidelines for physical activity.[5-7] Furthermore, the proportion meeting recommended levels of physical activity has been declining, particularly among girls (from 14% to 8%, 2008-2012).[8]

The disease burden of poor diet and physical inactivity on healthcare services is significant. In the UK, poor diet, and physical inactivity cost £7 billion to the National Health Service (NHS) annually.[9] Meeting current dietary recommendations would reduce years-of-life lost to coronary heart disease by 2 million, stroke by 400,000 and Type 2 diabetes by 19,000 over 20 years.[10]

Health behaviours in adolescence track into adulthood.[5 9 11 12] Therefore, sub-optimal diet and body composition in adolescence not only affect immediate physical and mental health but also increase the risk of NCDs in later life. Developmental plasticity in adolescence means that interventions to improve diet and levels of physical activity have the potential to reduce the trajectory of NCD risk over the life course.[13] While many adolescents find it difficult to engage with the long-term consequences of health behaviour, motivated and engaged adolescents can improve their health behaviours.[14 15] Evidence suggests that school-based interventions that offer combinations of peer-modelling, social support and choice, may be effective in improving diet and physical activity amongst adolescents.[16 17 18] Furthermore there is an increasing use of digital platforms by adolescents. According to 2018 estimates, 83% of 12-15 years olds in the UK own smartphones, with 99% spending an average of 20 hours per week online.[19 20] With an explosion in the use of such platforms to influence health behaviours in young people, they have potential as a complementary feature in complex interventions that aim to influence health behaviour in adolescents.[20]

Within this framework, a research programme Engaging Adolescents in CHanging Behaviour

For peer review only - http://bmjopen.bmj.com/site/about/guidelines.xhtml

(EACH-B) was designed to develop and test an intervention to encourage UK-based school students, aged 12-13 years, to adopt healthy behaviours such as eating better and exercising more (Trial registration: ISRCTN 74109264). EACH-B involves a cluster randomised controlled trial as a test of intervention effectiveness. Further details of the trial design are given elsewhere.[20] The research programme is funded by the National Institute for Health Research (RP-PG-0216-20004). The "LifeLab Plus" intervention developed as part of this programme is a complex three-part programme that comprises: i) an education module that teaches school students the science behind health messages through a 2-week module with a "hands-on" practical one-day visit to a teaching laboratory at University Hospital Southampton or in school while COVID-19 restrictions apply; ii) training for teachers in skills to support behaviour change; and iii) access to a specially-designed, interactive smartphone app with game features.

There is an emerging interest in identifying and developing interventions for improving diet and physical activity levels in adolescents. Taking this into consideration, we developed an illustrative decision-analytic model to assess the health benefits, costs, and cost-effectiveness of a multi-component intervention such as LifeLab Plus. This prototype model is designed to investigate how changes in diet quality and physical activity could affect future health outcomes and costs. We used cost data from the EACH-B programme and effectiveness estimates for similar interventions from published literature. The aims of this paper are to describe the structure, assumptions and parameters for our prototype model, to present preliminary (prior) estimates of cost-effectiveness based on currently available information and explore the sensitivity of results to key uncertainties. The model will be updated when results from the EACH-B trial are available.

METHODS

We developed a prototype *de novo* Markov model to estimate the costs, benefits and cost-effectiveness of school-based interventions that aim to improve diet quality and levels of physical activity compared with usual schooling for a cohort of adolescents. The model focussed on four potential short to medium-term benefits of healthy eating and physical activity: better mental health (episodes of depression and generalised anxiety disorder), higher earnings and reduced incidence of Type 2 diabetes and adverse pregnancy outcomes

For peer review only - http://bmjopen.bmj.com/site/about/guidelines.xhtml

(in terms of pre-term delivery). The model assumed that improved diet quality and increased physical activity would impact these four aspects of health via a reduction in BMI. Discussion with key project stakeholders reiterated these four benefits as the most relevant obesity-related effects in this population.

The model did not include later life impacts on cardiovascular disease or other chronic diseases as the impact on these outcomes of an intervention undertaken as an adolescent is uncertain. The model also investigated independent effects of physical activity on diabetes and depression (i.e. direct impacts not mediated by BMI). Information relating to epidemiology, mortality, effectiveness, health-related quality-of-life and costs was obtained from a variety of sources to inform the model parameters and assumptions.

Structuring the model

Population

A cohort of 100 adolescents with an equal proportion of boys and girls and a mean age of 13 years entered the model. The exemplar intervention is based on LifeLab Plus[20] and compared with usual schooling.

Model states

The Markov model consisted of three health states as described below (Figure 1). Outcomes associated with mental health, loss of earnings and adverse pregnancy outcome were incorporated as model events.

- *Healthy*: Adolescents enter the model in this state where they are assumed to have no other comorbidities including Type 2 diabetes or mental health problems. In each model cycle, a proportion will develop Type 2 diabetes and move to the *'Diabetes'* state or they may experience- mental health events, -pre-term births (girls only) or -loss of earnings. The latter three are treated as transient states.
- *Diabetes:* A proportion of adolescents with Type 2 diabetes may enter the transient states, as stated above.
- *Death:* This is an absorbing state. Adolescents may transition to this state either through general population mortality or death from- mental health conditions or -Type 2 diabetes and related complications.

For peer review only - http://bmjopen.bmj.com/site/about/guidelines.xhtml

Mental health encompasses a wide spectrum of conditions. Therefore, a pragmatic approach was adopted to include the two most prevalent mental health conditions for adolescents: clinical depression (henceforth, referred as depression) and General Anxiety Disorder (GAD). These conditions were categorised as: chronic (history of persistent mental illness), intermittent (experiencing intermittent episodes), and new onset (a one-time episode). Adverse pregnancy outcome was defined by pre-term delivery categorised as: extremely pre-term (delivery <28 weeks); very pre-term (delivery <33 weeks); and moderately pre-term (delivery between 34-36 weeks). The pregnancy outcome was applicable for girls only.

Each health state and event are associated with an impact on health-related quality-of-life (HRQoL) and excess cost. The model included intervention cost associated with LifeLab Plus and healthcare costs associated with- Type 2 diabetes, mental health events, pre-term delivery and loss of earnings due to obesity. In each model cycle, the total costs and QALYs are calculated by multiplying the individual costs and HRQoL by the number of people in the cohort still alive for each of the intervention and control arms. The total lifetime costs and QALYs are calculated by aggregating the costs and QALYs for all cycles.

Persistence of effects and time horizon

Adolescents were assumed to receive the intervention at the model entry, the duration of which lasted for a year. Although there is good epidemiological evidence of long-term tracking of health behaviour,[5,9,11,12] school-based trials rarely follow up for more than a year.[1,21,22] The persistence of intervention effect in terms of behaviour change is therefore uncertain. Hence, we take a conservative approach and focus on potential impacts of improved diet and physical activity from such an intervention likely to manifest in the short to medium term: up to a maximum time horizon of 20 years. However, due to the uncertainty, the treatment effect on behaviour was not assumed to last for 20 years. Based on our experts' judgement, for the base case the effect was assumed to sustain for 4 years, waning to no effect over a period of 10 years. We explored alternative assumptions about the persistence of effects on behaviour after trial follow-up.

The model time horizon was 20 years with a yearly cycle length. The time horizon was considered appropriate as the mean age of adolescents entering the model was 13 years as in the EACH-B trial. Costs and outcomes were half-cycle corrected.

For peer review only

For peer review only - http://bmjopen.bmj.com/site/about/guidelines.xhtml

Populating the model

Targeted literature searches were conducted to identify sources to inform model parameters. These are discussed below and in Appendices A and B.

Epidemiological data

Data on the relationship between mean BMI, age and sex was taken from Health Survey for England.[23] For adolescents aged ≤19 years, BMI z-scores are normally used. Therefore, we rescaled the values to relate relative risks to BMI z-scores, where:

BMI z-score = (observed value – median value of the reference population) / standard deviation value of reference population.

Physical activity levels for children aged 13-15 years were taken from Health Survey England, 2015.[24] The incidence of Type 2 diabetes in the UK was based on an analysis of longitudinal electronic health records in the Health Improvement Network (THIN) primary care database.[25] The prevalence of depressive episodes and GAD was taken from the Adult Psychiatric Morbidity Survey 2014[26] and from Mental Health of Children and Young people in England 2017.[27] The proportion in each category was assumed as follows: 17% had a chronic and 40% had a fluctuating (intermittent) course, while 43% remitted (new one-time episode).[28] Those individuals with depression or anxiety are at higher risk of suicide than the general population.[29] The excess death rate for those with depression and anxiety was calculated by using the suicide rate in the UK from Office of National Statistics (ONS) 2017 and applying a relative risk of 10.9 for depression and anxiety.[29] The proportions of pre-term deliveries, obtained from ONS 2017 data, were assumed as follows: 0.5% of total births as extremely pre-term (<28 weeks), 1.2% very pre-term birth (28 to <33 weeks) and 6.3% moderately pre-term birth (33-36 weeks) respectively.[30]

Relationship between BMI and risks of health events

The economic model assumed a positive correlation between increased BMI and the risks of - Type 2 diabetes, depression and GAD, pre-term delivery and loss of earnings. We fitted equations to the BMI relative risks. Hazard ratios, obtained from the Medicare Current Beneficiary Survey 1991-2010, were used to estimate the increased risk of individuals with higher BMI developing Type 2 diabetes.[31] The odds of depression and GAD in obese and

For peer review only - http://bmjopen.bmj.com/site/about/guidelines.xhtml

overweight adolescents compared with normal-weight adolescents were obtained from Sutaria et al.[32] This systematic review included 22 observational studies published between 2000 and 2017, representing 143,603 children. The relative risk of pre-term birth for mothers with overweight and obesity was obtained from Mcdonald et al.[33] The relative risk was assumed to be the same for all three categories of pre-term births.

Relationship between physical activity and risk of health event

The direct effect of physical activity on developing depression is modelled independent of the effect of physical activity via BMI. The odds ratio of developing depression was assumed to be 0.83 (95% CI 0.79-0.88) in those with high levels of physical activity compared to those with lower levels.[34] Furthermore, an increase from being inactive to achieving 150 minutes of moderate-intensity physical activity per week was assumed to lower the risk of Type 2 diabetes incidence by 26%, after adjustment for body weight.[35] The pooled odds ratio between Type 2 diabetes and risk of depression was 1.33 (95% CI, 1.18-1.51).[36]

Intervention effect

The intervention effect was based on three systematic reviews and meta-analyses that estimated the overall effects of school-based obesity prevention interventions.[37 38 39] The results of the meta-analyses were found to be significantly different between groups based on BMI (− 0.17 (95% CI− 0.29, − 0.06) kg/m^2)[38] and BMI z-score (− 0.06 (95% CI -0.10, − 0.03))[39] for multi-component interventions including physical activity, health education and dietary improvement. The effect of school-based intervention was assumed to increase the level of moderate or vigorous physical activity in children and adolescents by 4.84 min/day (95% CI −0.94 to 10.61).[40]

Mortality

General population mortality, adjusted for age and gender, was based on ONS 2020.[41] A hazard ratio of mortality of 2.98 was applied for individuals with Type 2 diabetes and aged between 35 and <65 years.[42]

Quality-of-life

EQ-5D-3L estimates adjusted for age, gender and BMI were used to estimate quality-of-life (Table 1). These estimates were obtained from 14,117 participants aged ≥16 years from the Health Survey for England 2008.[43] Adults with diabetes were assumed to have a disutility of -

For peer review only - http://bmjopen.bmj.com/site/about/guidelines.xhtml

0.161.[44] We estimated the disutility by comparing the mean general population EQ-5D score with that for people with diabetes. A disutility of -0.087 is used for adolescents with Type 2 diabetes based on a Swedish cohort of adolescents aged 13-18 years.[45] A utility decrement of 0.188 was assumed for those with intermittent episodes of mental health conditions;[46] a decrement of 0.488 for those with persistent/chronic depression;[47] a decrement of 0.094 (half of the decrement for intermittent depression) was assumed for those with a new episode of depression. For pre-term delivery, a mean utility decrement of 0.066 was applied throughout the model time horizon. This was based on a systematic review and meta-analysis for health utility values associated with pre-term birth where all but one study used Health Utilities Index (HUI) Mark 2 (HUI2) or Mark 3 (HUI3) measures as their primary health utility assessment method.[48] We found no evidence for quality-of-life loss in parents of pre-term babies. Therefore, we assumed that the quality-of-life decrement would be like intermittent mental health condition and lasts for the first two years.

Costs

We describe the costs below and summarise in Table 1.

Intervention costs

The intervention cost for our illustrative analysis was based on LifeLab Plus. Further information on resources used for delivering LifeLab are in Appendix B. The cost of the app delivered as part of the intervention, was incorporated as a capital cost and was assumed to last 10 years and be used in 10 centres. Similarly, the cost of setting up LifeLab Plus in a different centre was assumed to- consist of one year's staff costs and last for 10 years. Maintenance costs were estimated at 25% of the development cost per year. Overheads were included according to the rates used in Curtis et al. 2019[49]: direct overheads based on 29% of direct care salary costs and indirect overheads based on 16% direct care salary costs.

Health state costs

Both direct and indirect costs relating to individuals with Type 2 diabetes,[50] depression,[51] pre-term birth,[52] and loss of earnings were included. Indirect costs included the effect of depression on income and productivity. Costs were updated to 2019 prices using the hospital and community health services index.[49]

For peer review only - http://bmjopen.bmj.com/site/about/guidelines.xhtml

Direct health costs and indirect societal and productivity costs were estimated using a top-down approach. We assumed that individuals with Type 2 diabetes would not incur costs for complications as these are likely to affect individuals who have diabetes for a longer period. For costs associated with pre-term delivery, total societal costs for children born at 32-33 weeks and 34-36 weeks gestation from birth to 24 months were based on a study that compared these costs to those for children born at full-term.[53] The difference in costs for children born <28 weeks and 28-33 weeks were assumed to be same when compared to those born at full-term.

The lifetime indirect costs for overweight and obesity in childhood and adolescence is based on a study by Hamilton et al.[54] Mean total lifetime healthcare and productivity costs were estimated in Irish Euros, which were converted to GBP (£) and adjusted according to the average wage in the UK.

Validation

The structure of the prototype model was validated by the study team comprising epidemiologists, statisticians, trialists, public health experts and health economists. Internal validity of the model was established ensuring that the model predictions were consistent with the model specification.

Cost-effectiveness analysis

Costs and effects were estimated in accordance with the NICE Reference case,[55] except for the perspective adopted; we used a societal perspective that included both direct and indirect costs. Costs and health effects (QALYs) were discounted at 3.5% per year. The incremental cost-effectiveness ratio (ICER) was estimated as a ratio of the incremental costs of the intervention (LifeLab Plus) relative to the comparator (usual schooling) to the incremental QALYs of the intervention relative to the comparator. The intervention was considered cost-effective if the ICER was below the threshold of £20,000 per QALY gained, the lower threshold usually considered for the English NHS by NICE.[55]

Sensitivity analyses

To assess the uncertainty around the model predictions, we conducted deterministic sensitivity analysis (DSA), probabilistic sensitivity analyses (PSA) and scenario analyses. For the DSA, input parameters were varied by 95% confidence intervals (where available) or

For peer review only - http://bmjopen.bmj.com/site/about/guidelines.xhtml

varied by 10% of the mean. Monte Carlo simulations of 10,000 iterations were run for the PSA to assess the combined effects of input parameter uncertainties where parameters were simultaneously sampled within a specified distribution. We used standard distributions for the PSA, as recommended by Briggs et al.[56]: effectiveness parameters were assigned beta and lognormal distributions; utilities were assumed to follow a beta distribution; and costs were assigned a gamma distribution (Table 1 and further details in Appendix A). Scenario analyses were conducted to assess structural uncertainties related to model assumptions. Uncertainty about the sustainability of the intervention effect was assessed by varying the duration of intervention effect and its waning period.

The model was developed and implemented in Microsoft Excel.

Patient and public involvement

The research questions addressed in the overarching research programme EACH-B were informed by public involvement. Furthermore, representatives from public involved in the EACH-B research programme were presented the conceptual framework, modelling approaches, and invited to comment.

RESULTS

Base case analysis

For the base case, the exemplar intervention based on the multi-component LifeLab Plus was associated with higher costs and better health outcomes (more QALYs) compared with usual schooling. LifeLab Plus was associated with a mean QALY gain of 0.0085 at an incremental cost of £123 per person compared with usual schooling, resulting in an ICER of £14,367 per QALY (Table 2).

Sensitivity analyses

The results of the one-way sensitivity analyses are presented in the Tornado plot (Figure 2). Parameters including the intervention effect on levels of physical activity (expressed in terms of minutes of moderate or vigorous physical activity), quality-of-life gain for high levels of physical activity, duration of the intervention effect and duration of the treatment waning period had the highest impact on the cost-effectiveness results. Other parameters such as

For peer review only - http://bmjopen.bmj.com/site/about/guidelines.xhtml

effect of physical activity on BMI, time horizon and intervention costs also influenced the base case results, but to a lesser extent. The results of the PSA are presented as a cost-effectiveness scatter plot in Figure 3. All the iterations lie in the North-East quadrant of the cost-effectiveness plane, thereby indicating that the intervention is likely to produce health benefits at an additional cost. At a willingness-to-pay threshold of £20,000 per QALY gained, the probability of LifeLab Plus being cost-effective was 69% compared with usual schooling (Appendix C).

DISCUSSION

Main findings

Based on our prototype economic model, we estimate that a multi-component intervention to improve dietary quality and physical activity, such as LifeLab Plus, is likely to be considered cost-effective under conventional willingness-to-pay thresholds of £20,000–£30,000 per QALY gained in the UK. For our base case, the duration of the treatment effect was assumed to sustain for 4 years with the effect waning over a further period of 10 years. Our sensitivity analyses showed that if the duration of the treatment effect was not sustained to this extent, the intervention would be less cost-effective (Table 2). Changes to physical activity had a greater effect on the model results than changes in BMI. This is largely because physical activity is associated with an additional improvement in quality-of-life, independent of any health benefits associated with the health states in our model.

Comparison with previous models

Previous cost-effectiveness studies evaluating dietary and physical activity interventions for adolescents have largely consisted of within-trial analyses that have not considered the benefits beyond the trial period.[57-61] Cost-effectiveness estimates vary between a cost saving of £408.22 (NZ$835) per child for a low intensity program[57] to an additional cost of £120,630 per QALY gained for a more intensive intervention.[60] However, comparison between studies is difficult because of the differences in- the study designs, the interventions considered and the outcomes reported.

Gc et al[1] developed a model to assess the long-term costs and health outcomes of two physical activity interventions targeting adolescents in the UK. The cost-effectiveness estimates varied from £11,426 per QALY for an after-school intervention to £68,056 per

For peer review only - http://bmjopen.bmj.com/site/about/guidelines.xhtml

For peer review only

QALY for a multicomponent intervention. The costs of these interventions were estimated at £51 per participant for the after-school intervention and £207 (AUS$394) for the multicomponent intervention. Their model included different health states to our model for diseases which typically affect people in later life including chronic heart disease, stroke, heart failure, breast failure and colorectal cancer. They ran the model for a lifetime horizon of 65 years. We have not included these health states as we adopted a conservative assumption that treatment benefits for adolescents from such multicomponent intervention as LifeLab Plus do not persist beyond 20 years.

Strengths and limitations

The study addresses an important public health question by examining whether interventions targeting healthy eating and increased physical activity for adolescents provide value for money from a societal perspective. We incorporate existing evidence on the effect of improvement in adolescent health behaviours on four high prevalence short-to-medium term benefits relevant to adolescents: improved mental health, higher earnings, improved pregnancy outcomes and prevention of Type 2 diabetes. Sources for data used within our model were identified from a targeted literature review. Where data were not available for adolescents, we used data from adult population. Model structure and assumptions were informed by this review and discussions with public health experts. UK-specific incidence rates were used to ensure that patients entering the model matched the likely distribution of events in the UK. Our analysis focuses on a pragmatic subset of specific outcomes, while noting these could be expanded on with emerging data on effectiveness, notably in relation to mental health and other long-term conditions.

We do not attempt to estimate the long-term impacts of such interventions due to the lack of longitudinal data on lifetime trajectories of healthy diet and increased levels of physical activity. Our preliminary model suggests that this type of intervention is likely to be cost-effective based on short-to-medium term effects alone. If this is correct and the effects last longer, then additional benefits such as prevention of cardiovascular disease would enhance the cost-effectiveness of these interventions. This suggests that our cost-effectiveness results are conservative, and that quantification of long-term benefits is not necessary to demonstrate the cost-effectiveness of this type of intervention. However, the sensitivity analyses reported above do indicate some important uncertainties.

For peer review only - http://bmjopen.bmj.com/site/about/guidelines.xhtml

Our model has several shortcomings. The EACH-B study, which is the main trial evidence, is ongoing so there is currently no direct data on the effectiveness of this intervention. We have used evidence on treatment effectiveness from a systematic review of related interventions. When results become available from the EACH-B study, they will add to the evidence base and reduce uncertainty in these results. Further evidence for other model parameters would also be valuable. For example, where data from adolescent age groups were not available, we used epidemiological estimates from adult populations that might not be transferable. There is uncertainty around key assumptions related to the duration of benefits from the intervention. In our analysis, we have assumed that the benefit observed in the clinical trials will last for 4 years and then will gradually reduce over the next 10 years. We only include costs and effects over a 20-year time horizon and have assumed that there would be no further benefits of the intervention for chronic diseases such as cardiovascular diseases and diabetes. There are also limitations in our approach to estimating income lost due to obesity. We have adopted a simple approach; however, this is a complex interacting bi-directional system. We have not fully explored whether income loss is due to obesity or whether obesity is caused by income loss through, for example, unemployment. Other broader uncertainties, such as long-term effects of COVID on behaviour and mental health are also not addressed.

CONCLUSION

Complex behavioural interventions that aim to improve diet and increase levels of physical activity amongst school-aged children have the potential to provide cost-effective use of UK healthcare resources. Such interventions have the potential to reduce burden of NCDs, although benefits in later life are more sensitive to assumptions about the persistence of behavioural change and discounting. Our analysis focuses on a pragmatic subset of specific outcomes based on published literature on effectiveness of similar interventions, while noting these could be expanded on with emerging data on effectiveness, notably in relation to mental health and other long-term conditions.

Word count: 4188

For peer review only - http://bmjopen.bmj.com/site/about/guidelines.xhtml

Author Contributions NK designed the model, performed the analysis, interpreted the analysis, and wrote the first draft of the manuscript. KC designed the model and performed the analysis. JL supervised the process, provided comments on model structure and data analysis. KWT provided inputs for model parameters. JB, KMG, HI, MB and CC provided critical comments on the model structure. All authors contributed to the critical revision of the manuscript and approved the final version of the manuscript.

Ethics approval Not required as the model is informed by data from the published literature.

Funding The research is funded by the National Institute for Health Research (RP-PG-0216-20004). HMI and KMG are funded by the UK Medical Research Council (MC_UU_12011/4). KMG is supported by the National Institute for Health Research (NIHR Senior Investigator (NF-SI-0515-10042), NIHR Southampton 1000DaysPlus Global Nutrition Research Group (17/63/154) and NIHR Southampton Biomedical Research Centre (IS-BRC-1215-20004)), the European Union (Erasmus+ Programme ImpENSA 598488-EPP-1-2018-1-DE-EPPKA2-CBHE-JP) and the British Heart Foundation (RG/15/17/3174).

Competing Interests KMG has received reimbursement for speaking at conferences sponsored by companies selling nutritional products and is part of an academic consortium that has received research funding from Abbott Nutrition, Nestec, BenevolentAI Bio Ltd. and Danone. The other authors have no potentially competing interests to declare.

Patient consent for publication Not required.

Data availability statement Data from publicly available sources were used for the model. All model inputs are described in the paper.

For peer review only

For peer review only - http://bmjopen.bmj.com/site/about/guidelines.xhtml

Table 1 Input parameters used in the model

**Parameter**	Value	SD/SE	95% CI-Low	95% CI-High	Distribution	Reference
**Utility inputs**						
Disutility for Diabetes for 13-18 years old	-0.087	0.036	0.016	0.158	Beta	[45]
Disutility for Diabetes	-0.161	0.040	0.240	0.080	Beta	[44]
Disutility for pre-term babies < 33 weeks	-0.066	0.016	0.098	0.035	Beta	[48]
Disutility for parents of pre-term babies < 33 weeks	-0.094	0.019	0.057	0.131	Beta	Assumption
Disutility for depression	-0.188	0.038	0.114	0.262	Beta	[46]
Disutility for Chronic mental health condition	-0.448	0.036	0.377	0.519	Beta	[47]
Disutility for Intermittent mental health condition	-0.188	0.038	0.114	0.262	Beta	[46]
Disutility for New cases mental health condition	-0.094	0.019	0.057	0.131	Beta	Assumption
Quality-of-life gain for high activity	0.020	0.002	0.016	0.024	Beta	
**Direct costs**						
Type 2 diabetes	£622	£62	£500	£744	Gamma	[50]
Mental health condition	£1,334	£383	£585	£2,307	Gamma	[51]
Mental health condition- chronic	£4,233	£629	£3,001	£7,238	Gamma	[51]

For peer review only

Mental health condition- intermittent	£1,334	£383	£585	£2,307	Gamma	[51]
Mental health condition-new onset	£667	£191	£292	£1,042	Gamma	[51]
Pre-term birth <28 weeks	£25,452	£2,169	£21,201	£29,704	Gamma	[52;53]
Pre-term birth 28-33 weeks	£13,073	£1,114	£10,889	£15,256	Gamma	[52;53]
Pre-term birth, 34-36 weeks	£4,157	£1,020	£2,157	£6,157	Gamma	[52;53]
**Indirect costs**						
Type 2 diabetes	£4,116	£412	£3,309	£4,923	Gamma	[50]
Mental health condition	£223	£64	£98	£348	Gamma	[51]
Mental health condition- chronic	£707	£203	£310	£1,105	Gamma	[51]
Mental health condition- intermittent	£223	£64	£98	£348	Gamma	[51]
Mental health condition-new onset	£112	£32	£49	£174	Gamma	[51]
Pre-term birth <28 weeks	£397	£114	£174	£619	Gamma	[52;53]
Pre-term birth 28-33 weeks	£119	£34	£52	£185	Gamma	[52;53]
Pre-term birth, 34-36 weeks	£103	£29	£45	£161	Gamma	[52;53]
**Cost of the intervention**	£155	£15	£124	£185	Gamma	Estimated
SD: Standard Deviation; SE: Standard Error; CI: Confidence Interval						

For peer review only - http://bmjopen.bmj.com/site/about/guidelines.xhtml

Table 2 Incremental cost-effectiveness ratios in the base case and scenario analyses

	Total cost (£)	Total QALYs	Incremental cost (£)	Incremental QALY	ICER
**Base case analysis**					
Usual schooling	£4,416	13.07			
LifeLab Plus	£4,539	13.08	£123	0.0085	£14,367
**Perspective: NHS**					
Usual schooling	£2,001	13.07			
LifeLab Plus	£2,141	13.08	£140	0.0085	£16,339
**Costs discounted at 3.5%; QALYs at 1.5% per annum**					
Usual schooling	£4,416	15.76			
LifeLab Plus	£4,539	15.77	£123	0.0097	£12,640
**Costs and QALYs discounted at 1.5%**					
Usual schooling	£5,813	15.76			
LifeLab Plus	£5,930	15.77	£117	0.0097	£12,044
**Time Horizon: 5 years**					
Usual schooling	£488	4.25			
LifeLab Plus	£633	4.25	£145	0.0043	£33,423
**Time Horizon: 10 years**					
Usual schooling	£1,132	7.76			
LifeLab Plus	£1,265	7.77	£134	0.0071	£18,851
**Time Horizon: 30 years**					
Usual schooling	£11,137	16.67			
LifeLab Plus	£11,258	16.67	£121	0.0088	£13,719
**Time Horizon: 40 years**					
Usual schooling	£18,417	19.05			
LifeLab Plus	£18,537	19.06	£120	0.0089	£13,455
**Duration of treatment effect: 1 year & Treatment waning: 2 years**					
Usual schooling	£4,416	13.07			
LifeLab Plus	£4,566	13.08	£150	0.0030	£49,758
**Duration of treatment effect: 1 years & Treatment waning: 5 years**					
Usual schooling	£4,416	13.07			
LifeLab Plus	£4,563	13.08	£147	0.0043	£34,089
**Duration of treatment effect: 2 years & Treatment waning: 5 years**					
Usual schooling	£4,416	13.07			
LifeLab Plus	£4,560	13.08	£144	0.0051	£28,034
**Duration of treatment effect: 5 years & Treatment waning: 5 years**					
Usual schooling	£4,416	13.07			
LifeLab Plus	£4,553	13.08	£137	0.0075	£18,218
**Duration of treatment effect: 5 years & Treatment waning: 10 years**					
Usual schooling	£4,416	13.07			
LifeLab Plus	£4,529	13.08	£113	0.0092	£12,184
**Duration of treatment effect: 10 years & Treatment waning: 10 years**					
Usual schooling	£4,416	13.07			
LifeLab Plus	£4,432	13.09	£16	0.0125	£1,257
**Duration of treatment effect: 15 years & Treatment waning: 5 years**					
Usual schooling	£4,416	13.07			

For peer review only

Table 2 Incremental cost-effectiveness ratios in the base case and scenario analyses

	Total cost (£)	Total QALYs	Incremental cost (£)	Incremental QALY	ICER
LifeLab Plus	£4,308	13.09	-£108	0.0147	Dominates

ICER, incremental cost-effectiveness ratio (incremental cost/incremental QALY); QALY, quality-adjusted life year.

List of Figure legends

- Figure 1 provides an illustration of the conceptual framework for the cost-effective model and an illustration of the model structure that shows transition of the modelled population through the modelled health states.
- Figure 2 provides an illustration of the Tornado diagram obtained from the deterministic sensitivity analysis that shows the impact of varying the input values of the parameters that have the highest impact on the cost-effectiveness result.
- Figure 3 provides an illustration of the scatter plot obtained from the probabilistic sensitivity analysis. Results from all the 10,000 simulations fall within the North-East quadrant of the cost-effectiveness plan thereby indicating that the intervention (LifeLab Plus) is more expensive and more effective compared to usual schooling.

For peer review only

REFERENCES

1. Gc VS, Suhrcke M, Atkin AJ, et al. Cost-effectiveness of physical activity interventions in adolescents: model development and illustration using two exemplar interventions. *BMJ Open* 2019;9(8):e027566. doi: 10.1136/bmjopen-2018-027566 [published Online First: 2019/08/21]
2. Organisation WH. Unhealthy Diets & Physical Inactivity 2009 [Available from: https://www.who.int/nmh/publications/fact_sheet_diet_en.pdf accessed 19/11/2020.
3. Organisation WH. Adolescent Health 2020 [Available from: https://www.who.int/health-topics/adolescent-health/#tab=tab_1 accessed 13/11/20.
4. Viner RM, Ross D, Hardy R, et al. Life course epidemiology: recognising the importance of adolescence. *Journal of Epidemiology and Community Health* 2015;69(8):719-20. doi: 10.1136/jech-2014-205300
5. Rose T, Barker M, Maria Jacob C, et al. A Systematic Review of Digital Interventions for Improving the Diet and Physical Activity Behaviors of Adolescents. *J Adolesc Health* 2017;61(6):669-77. doi: 10.1016/j.jadohealth.2017.05.024 [published Online First: 2017/08/16]
6. Bates B, Lennox A, Prentice A, et al. National Diet and Nutrition Survey: Results from Years 1, 2, 3 and 4 (combined) of the Rolling Programme (2008/2009-2011/2012): a Survey Carried Out on Behalf of Public Health England and the Food Standards Agency: Public Health England, 2014.
7. Woods-Townsend K, Bagust L, Barker M, et al. Engaging teenagers in improving their health behaviours and increasing their interest in science (Evaluation of LifeLab Southampton): study protocol for a cluster randomized controlled trial. *Trials* 2015;16:372-72. doi: 10.1186/s13063-015-0890-z
8. NHS. Health Survey for England 2015 Physical activiy in children 2016 [Available from: http://healthsurvey.hscic.gov.uk/media/37752/hse2015-child-phy-act.pdf accessed 17/11/20.
9. term TNL. 2019 [Available from: https://www.longtermplan.nhs.uk/publication/nhs-long-term-plan accessed 15/09/20.
10. Milner J, Green R, Dangour AD, et al. Health effects of adopting low greenhouse gas emission diets in the UK. *BMJ Open* 2015;5(4):e007364. doi: 10.1136/bmjopen-2014-007364
11. Kelder SH, Perry CL, Klepp KI, et al. Longitudinal tracking of adolescent smoking, physical activity, and food choice behaviors. *Am J Public Health* 1994;84(7):1121-26. doi: 10.2105/ajph.84.7.1121
12. Craigie AM, Lake AA, Kelly SA, et al. Tracking of obesity-related behaviours from childhood to adulthood: A systematic review. *Maturitas* 2011;70(3):266-84. doi: 10.1016/j.maturitas.2011.08.005 [published Online First: 2011/09/17]
13. Hanson M, Gluckman P. Developmental origins of noncommunicable disease: population and public health implications. *Am J Clin Nutr* 2011;94(6 Suppl):1754s-58s. doi: 10.3945/ajcn.110.001206 [published Online First: 2011/04/29]
14. Gates M. Advancing the adolescent health agenda. *The Lancet* 2016;387(10036):2358-59. doi: 10.1016/S0140-6736(16)30298-7
15. Organisation WH. Global Accelerated Action for the Health of Adolescents (AAHA!): guidance to support country implementation. Geneva, 2017.
16. Kelishadi R, Azizi-Soleiman F. Controlling childhood obesity: A systematic review on strategies and challenges. *J Res Med Sci* 2014;19(10):993-1008.
17. Calvert S, Dempsey RC, Povey R. Delivering in-school interventions to improve dietary behaviours amongst 11- to 16-year-olds: A systematic review. *Obesity Reviews* 2019;20(4):543-53. doi: https://doi.org/10.1111/obr.12797

For peer review only - http://bmjopen.bmj.com/site/about/guidelines.xhtml

18. Woods-Townsend K, Hardy-Johnson P, Bagust L, et al. A cluster-randomised controlled trial of the LifeLab education intervention to improve health literacy in adolescents. *PLoS One* in press
19. Ofcom. Children and parents: Media use and attitudes report 2019 [Available from: https://www.ofcom.org.uk/__data/assets/pdf_file/0023/190616/children-media-use-attitudes-2019-report.pdf accessed 10/09/20.
20. Strömmer S, Barrett M, Woods-Townsend K, et al. Engaging adolescents in changing behaviour (EACH-B): a study protocol for a cluster randomised controlled trial to improve dietary quality and physical activity. *Trials* 2020;21(1):859. doi: 10.1186/s13063-020-04761-w
21. Borde R, Smith JJ, Sutherland R, et al. Methodological considerations and impact of school-based interventions on objectively measured physical activity in adolescents: a systematic review and meta-analysis. *Obesity Reviews* 2017;18(4):476-90. doi: https://doi.org/10.1111/obr.12517
22. Buxton MJ, Drummond MF, Van Hout BA, et al. Modelling in Ecomomic Evaluation: An Unavoidable Fact of Life. *Health Economics* 1997;6(3):217-27. doi: https://doi.org/10.1002/(SICI)1099-1050(199705)6:3<217::AID-HEC267>3.0.CO;2-W
23. NHS Digital. Health Survey for England 2016. 2017.
24. Digital N. Health Survey for England 2015 [Available from: https://digital.nhs.uk/data-and-information/publications/statistical/health-survey-for-england/health-survey-for-england-20152020.
25. Sharma M, Nazareth I, Petersen I. Trends in incidence, prevalence and prescribing in type 2 diabetes mellitus between 2000 and 2013 in primary care: a retrospective cohort study. *BMJ Open* 2016;6(1):e010210. doi: 10.1136/bmjopen-2015-010210 [published Online First: 2016/01/16]
26. McManus S BP, Jenkins R, Brugha T. Mental health and wellbeing in England: Adult Psychiatric Morbidity Survey 2014. 2016.
27. NHS Digital. Mental health of children and young people in England, 2017. 2017.
28. Stegenga BT, Kamphuis MH, King M, et al. The natural course and outcome of major depressive disorder in primary care: the PREDICT-NL study. *Soc Psychiatry Psychiatr Epidemiol* 2012;47(1):87-95. doi: 10.1007/s00127-010-0317-9 [published Online First: 2010/11/09]
29. Singhal A, Ross J, Seminog O, et al. Risk of self-harm and suicide in people with specific psychiatric and physical disorders: comparisons between disorders using English national record linkage. *J R Soc Med* 2014;107(5):194-204. doi: 10.1177/0141076814522033 [published Online First: 2014/02/15]
30. Office of National Statistics. Births in England and Wales: 2017. 2018.
31. Gray N, Picone G, Sloan F, et al. Relation between BMI and diabetes mellitus and its complications among US older adults. *South Med J* 2015;108(1):29-36. doi: 10.14423/SMJ.0000000000000214 [published Online First: 2015/01/13]
32. Sutaria S, Devakumar D, Yasuda SS, et al. Is obesity associated with depression in children? Systematic review and meta-analysis. *Arch Dis Child* 2019;104(1):64-74. doi: 10.1136/archdischild-2017-314608 [published Online First: 2018/07/01]
33. McDonald SD, Han Z, Mulla S, et al. Overweight and obesity in mothers and risk of preterm birth and low birth weight infants: systematic review and meta-analyses. *BMJ* 2010;341:c3428. doi: 10.1136/bmj.c3428 [published Online First: 2010/07/22]
34. Schuch FB, Vancampfort D, Firth J, et al. Physical Activity and Incident Depression: A Meta-Analysis of Prospective Cohort Studies. *Am J Psychiatry* 2018;175(7):631-48. doi: 10.1176/appi.ajp.2018.17111194 [published Online First: 2018/04/25]

For peer review only - http://bmjopen.bmj.com/site/about/guidelines.xhtml

35. Wahid A, Manek N, Nichols M, et al. Quantifying the Association Between Physical Activity and Cardiovascular Disease and Diabetes: A Systematic Review and Meta - Analysis. *Journal of the American Heart Association* 2016;5(9) doi: 10.1161/jaha.115.002495
36. Chireh B, Li M, D'Arcy C. Diabetes increases the risk of depression: A systematic review, meta-analysis and estimates of population attributable fractions based on prospective studies. *Prev Med Rep* 2019;14:100822. doi: 10.1016/j.pmedr.2019.100822 [published Online First: 2019/03/01]
37. Liu Z, Xu HM, Wen LM, et al. A systematic review and meta-analysis of the overall effects of school-based obesity prevention interventions and effect differences by intervention components. *Int J Behav Nutr Phys Act* 2019;16(1):95. doi: 10.1186/s12966-019-0848-8 [published Online First: 2019/10/31]
38. Lavelle HV, Mackay DF, Pell JP. Systematic review and meta-analysis of school-based interventions to reduce body mass index. *J Public Health (Oxf)* 2012;34(3):360-9. doi: 10.1093/pubmed/fdr116 [published Online First: 2012/01/24]
39. Jacob CM, Hardy-Johnson PL, Inskip HM, et al. A systematic review and meta-analysis of school-based interventions with health education to reduce body mass index in adolescents aged 10 to 19 years. *Int J Behav Nutr Phys Act* 2021;18(1):1. doi: 10.1186/s12966-020-01065-9 [published Online First: 2021/01/06]
40. Mears R, Jago R. Effectiveness of after-school interventions at increasing moderate-to-vigorous physical activity levels in 5- to 18-year olds: a systematic review and meta-analysis. *Br J Sports Med* 2016;50(21):1315-24. doi: 10.1136/bjsports-2015-094976 [published Online First: 2016/05/26]
41. Statistics OfN. National life tables: England. 2020. https://www.ons.gov.uk/peoplepopulationandcommunity/birthsdeathsandmarriages/lifeexpectancies/datasets/nationallifetablesenglandreferencetables (accessed 22/03/2021).
42. Mulnier HES, H.E.;Raleigh,V.S.;Soedamah-Muthu,S.S.;Colhoun,H.M.;Lawrenson,R.A.;. Mortality in people with Type 2 diabetes in the UK. *Diabetic Medicine* 2006(23):516-21.
43. Maheswaran H, Petrou S, Rees K, et al. Estimating EQ-5D utility values for major health behavioural risk factors in England. *J Epidemiol Community Health* 2013;67(2):172-80. doi: 10.1136/jech-2012-201019 [published Online First: 2012/07/31]
44. Janssen MF, Lubetkin EI, Sekhobo JP, et al. The use of the EQ-5D preference-based health status measure in adults with Type 2 diabetes mellitus. *Diabet Med* 2011;28(4):395-413. doi: 10.1111/j.1464-5491.2010.03136.x [published Online First: 2011/03/12]
45. Astrom M, Persson C, Linden-Bostrom M, et al. Population health status based on the EQ-5D-Y-3L among adolescents in Sweden: Results by sociodemographic factors and self-reported comorbidity. *Qual Life Res* 2018;27(11):2859-71. doi: 10.1007/s11136-018-1985-2 [published Online First: 2018/09/10]
46. Lynch FD, JF.; Feeny, DH.; Clarke, GN.; MacMillan, AL.;. Measuring Health-related Quality of Life in Teens With and Without Depression. *Medical Care* 2016;54(12):8.
47. Morriss R, Garland A, Nixon N, et al. Efficacy and cost-effectiveness of a specialist depression service versus usual specialist mental health care to manage persistent depression: a randomised controlled trial. *The Lancet Psychiatry* 2016;3(9):821-31. doi: 10.1016/s2215-0366(16)30143-2
48. Petrou S, Krabuanrat N, Khan K. Preference-Based Health-Related Quality of Life Outcomes Associated with Preterm Birth: A Systematic Review and Meta-analysis.

For peer review only - http://bmjopen.bmj.com/site/about/guidelines.xhtml

Pharmacoeconomics 2019 doi: 10.1007/s40273-019-00865-7 [published Online First: 2019/12/10]
49. Curtis LB, A.;. Unit costs of Health and Social Care 2018. 2018.
50. Hex N, Bartlett C, Wright D, et al. Estimating the current and future costs of Type 1 and Type 2 diabetes in the UK, including direct health costs and indirect societal and productivity costs. *Diabet Med* 2012;29(7):855-62. doi: 10.1111/j.1464-5491.2012.03698.x [published Online First: 2012/04/28]
51. Kuyken W, Hayes R, Barrett B, et al. Effectiveness and cost-effectiveness of mindfulness-based cognitive therapy compared with maintenance antidepressant treatment in the prevention of depressive relapse or recurrence (PREVENT): a randomised controlled trial. *The Lancet* 2015;386(9988):63-73. doi: 10.1016/s0140-6736(14)62222-4
52. Khan KA, Petrou S, Dritsaki M, et al. Economic costs associated with moderate and late preterm birth: a prospective population-based study. *BJOG* 2015;122(11):1495-505. doi: 10.1111/1471-0528.13515 [published Online First: 2015/07/30]
53. Mangham LJ, Petrou S, Doyle LW, et al. The cost of preterm birth throughout childhood in England and Wales. *Pediatrics* 2009;123(2):e312-27. doi: 10.1542/peds.2008-1827 [published Online First: 2009/01/28]
54. Hamilton D, Dee A, Perry IJ. The lifetime costs of overweight and obesity in childhood and adolescence: a systematic review. *Obes Rev* 2018;19(4):452-63. doi: 10.1111/obr.12649 [published Online First: 2017/12/23]
55. The National Institute for Health and Care Excellence. Guide to the methods of technology appraisal, 2013.
56. Briggs AH, Claxton K, Sculpher MJ. Decision Modelling for Health Economic Evaluation - Handbooks in Health Economic Evaluation Series. Oxford University Press. 2006.
57. Anderson YC, Leung W, Grant CC, et al. Economic evaluation of a multi-disciplinary community-based intervention programme for New Zealand children and adolescents with obesity. *Obesity Research & Clinical Practice* 2018;12(3):293-98. doi: https://dx.doi.org/10.1016/j.orcp.2018.04.001
58. Ladapo JA, Bogart LM, Klein DJ, et al. Cost and Cost-Effectiveness of Students for Nutrition and eXercise (SNaX). *Academic pediatrics* 2016;16(3):247-53. doi: https://dx.doi.org/10.1016/j.acap.2015.07.009
59. Lee BY, Adam A, Zenkov E, et al. Modeling The Economic And Health Impact Of Increasing Children's Physical Activity In The United States. *Health Affairs* 2017;36(5):902-08. doi: https://dx.doi.org/10.1377/hlthaff.2016.1315
60. Panca M, Christie D, Cole TJ, et al. Cost-effectiveness of a community-delivered multicomponent intervention compared with enhanced standard care of obese adolescents: cost-utility analysis alongside a randomised controlled trial (the HELP trial). *BMJ Open* 2018;8(2):e018640. doi: https://dx.doi.org/10.1136/bmjopen-2017-018640
61. Sutherland R, Reeves P, Campbell E, et al. Cost-effectiveness of a multi-component school-based physical activity intervention targeting adolescents: the 'Physical Activity 4 Everyone' cluster randomized trial. *International Journal of Behavioral Nutrition & Physical Activity* 2016;13:94. doi: https://dx.doi.org/10.1186/s12966-016-0418-2

For peer review only - http://bmjopen.bmj.com/site/about/guidelines.xhtml

For peer review only

For peer review only - http://bmjopen.bmj.com/site/about/guidelines.xhtml

Figure 1: Illustration of the conceptual framework and model structure

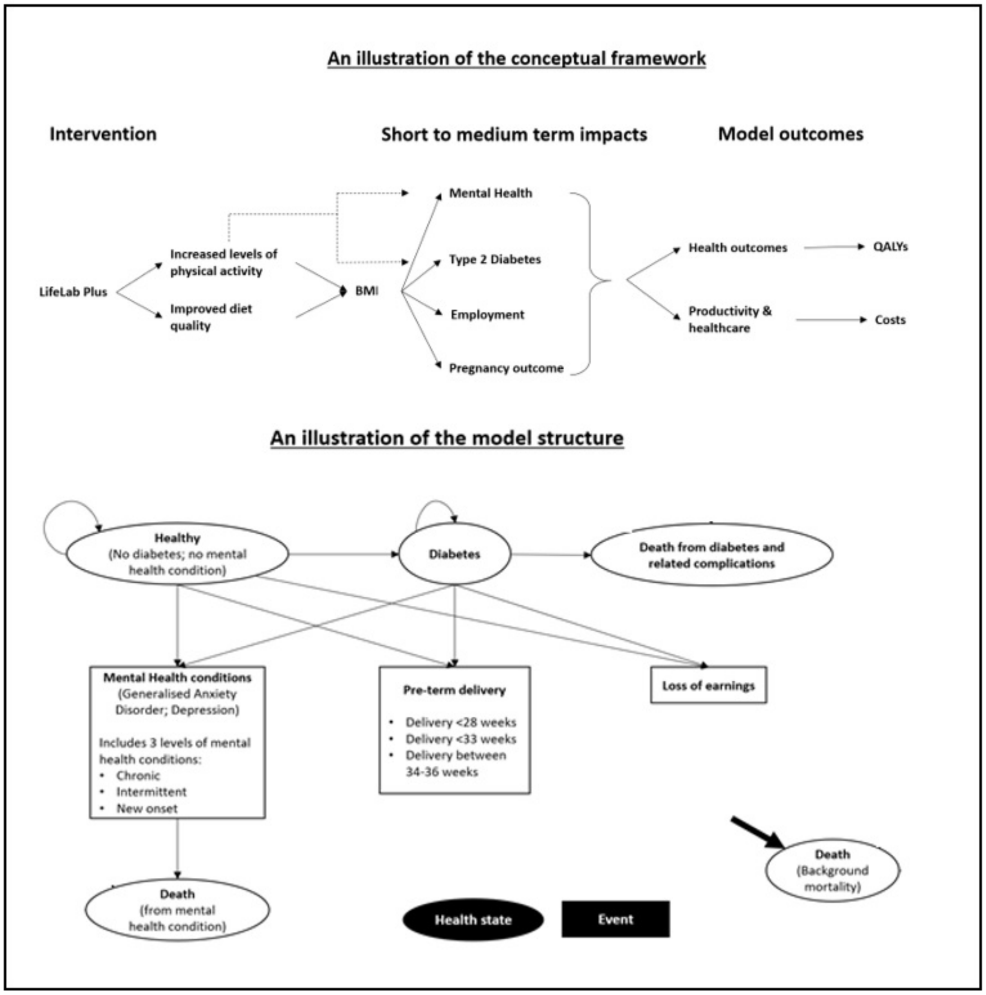

Figure 1: Illustration of the conceptual framework and model structure

137x145mm (144 x 144 DPI)

Figure 2: Tornado diagram to show the impact of varying parameter input values on ICERs

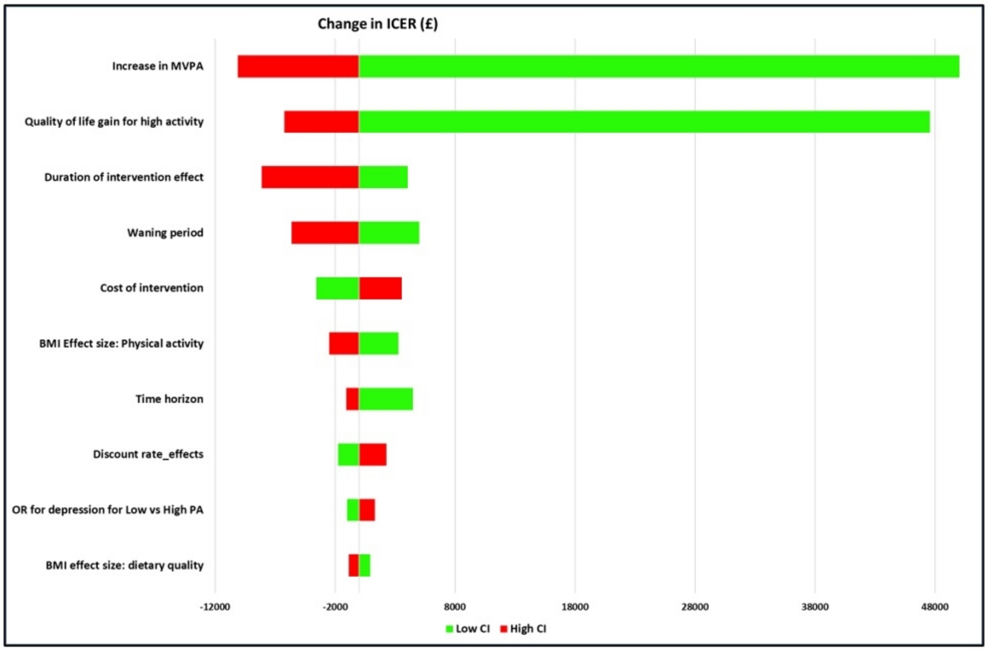

MVPA: Moderate or Vigorous Physical Activity; BMI: Body Mass Index; CI: Confidence Interval; ICER: Incremental Cost Effectiveness Ratio

Figure 2: Tornado diagram to show the impact of varying parameter input values on ICERs

175x127mm (144 x 144 DPI)

Figure 3: Scatter plot of PSA cost-effectiveness results

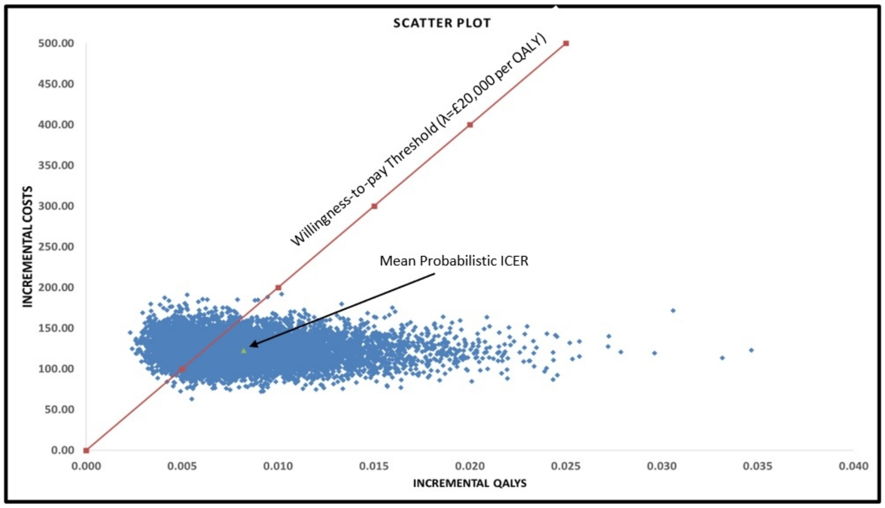

The probabilistic model was run for 10,000 simulations. All the plots fall in the North-East quadrant of the Cost-effectiveness plane, indicating the intevention- LifeLab Plus is associated with higher costs and higher QALYs compared to usual schooling. Majority of the plots, including the mean ICER, fall within the willingness-to-pay threshold of £20,000 per QALY.

Figure 3: Scatter plot of PSA cost-effectiveness results

199x122mm (144 x 144 DPI)

1 APPENDIX A

Model parameters were estimated by using the following equations. For the control arm, rate was based upon age only. For the intervention arm, rate was based on age and the change in BMI from the control population to the intervention.

Table 1: Parameter equations

Parameter	Equation	Units	Source
BMI - men	$-0.0031*age^2+0.4096*age+16.098$	Kg/m2	HSE 2017[1]
BMI - women	$-0.0032*Age^2 + 0.4235*Age+15.812$	Kg/m2	
Type 2 Diabetes Men Control, age <25y	0.025*Age-0.265	Incidence per 1000	Sharma 2015[2]
Type 2 Diabetes Men Control, age >25y	0.0054Age^2-0.191Age+1.7142	Incidence per 1000	
Type 2 Diabetes Women, Control, all ages	0.0031Age^2-0.0942Age+1.2045	Incidence per 1000	
Type 2 Diabetes Men, Intervention, age < 25y	(0.025*Age-0.265)/(0.081BMI_control-0.8451)*(0.081BMI_int-0.8451)	Incidence per 1000	Gray et al 2015[3]
Type 2 Diabetes Men, Intervention, > 25y	(0.0054Age^2-0.191Age+1.7142)/(0.081BMI_control-0.8451)*(0.081BMI_int-0.8451)	Incidence per 1000	
Type 2 Diabetes Women Intervention, all ages	(0.0031Age^2-0.0942Age+1.2045)/(0.0637BMI_control -0.3135)*(0.0637BMI_Int-0.3135)	Incidence per 1000	
Mental health Men, control	-0.00008*Age^2+0.0073*Age-0.0544	% Incidence	APMS 2014[4]
Mental health Women, control	-0.00007*Age^2+0.0062*Age+0.0034	% Incidence	
Mental Health Men, intervention	(-0.00007*Age^2+0.0062*Age+0.0034)*(0.0127*BMI_I+0.727)/(0.0127*BMI_C +0.727)	% Incidence	Sutaria et al 2018[5]
Mental Health Women, intervention	(-0.00007*Age^2+0.0062*Age+0.0034) *(0.0391*BMI_I+0.1087)/(0.0391*BMI_C+0.1087)	% Incidence	
Mental Health, diabetes	Multiply MH risk by 1.33		Chireh et al 2019[6]
Mental Health, Physical exercise	OR 0.83 high activity vs low activity. Applied to intervention arm only.		Schuch et al 2018[7]
Preterm birth <33 weeks, control	$-0.0512*Age^2+3.0922*Age-37.891$	Incidence per 1000	ONS 2017[8]
Preterm birth <33 weeks, intervention	(-0.0512*Age2+3.0922*Age-37.891)*(0.0514*BMI_I-0.1712)/(0.0514*BMI_C-0.1712)	Incidence per 1000	Mcdonald et al 2010[9]
Loss of earnings, men	12534BMI – 297703	£ over lifetime	

For peer review only - http://bmjopen.bmj.com/site/about/guidelines.xhtml

Parameter	Equation	Units	Source
Loss of earning, women	17695BMI - 439796	£ over lifetime	Hamilton et al 2018[10]

Table 2: Parameter equations for z-scores

Parameter	Equation	Units	Source
Mental Health Men, Intervention	(0.0054Age^2-0.191Age+1.7142)/(0.0274BMI_z_score_control+1.0011)*(0.0274BMI_z_scores_int+1.011)	Incidence per 1000	Gray et al 2015[3]
Mental Health Women, Intervention	(0.0031Age^2-0.0942Age+1.2045)/(0.084BMI_z_scores_control+0.9486)*(0.084BMI_z_scores_Int+0.9486)	Incidence per 1000	
Type 2 Diabetes Men, intervention	(-0.00007*Age^2+0.0062*Age+0.0034)/(0.1742BMI_z_scores_control+0.8964)*(0.1742BMI_z_scores_Int+0.8964)	% Incidence	Sutaria et al 2018[5]
Type 2 Diabetes Women, intervention	(-0.00007*Age^2+0.0062*Age+0.0034)*(0.1369*BMI_z_scores_I+1.0556)/(0.1369*BMI_z_scores_C+1.0556)	% Incidence	
Preterm birth <33 weeks, intervention	(-0.0512*Age2+3.0922*Age-37.891)*(0.1105*BMI_z_scores_I+0.9336)/(0.1105*BMI_z_scores_C+0.9336)	Incidence per 1000	Mcdonald et al 2010[9]

Table 3: Parameter equation for quality of life estimates

Parameter	Equation	Source
Quality of life, by age	0.9508566+0.0212126*Male-0.0002587*Age-0.0000332*Age^2	Maheswaran et al 2012[11]
Disutility for BMI, if BMI < 30	-0.0009BMI + 0.0189	
Disutility for BMI, if BMI > 30	-0.0036BMI+0.094	

For peer review only

For peer review only - http://bmjopen.bmj.com/site/about/guidelines.xhtml

Table 4: Input parameters used in the model

Parameter	Value	SD/SE	95% CI-Low	95% CI-High	Distribution
**Direct trial efficacy**					
BMI effect size: Physical activity	0.13	0.046	0.040	0.220	Beta
BMI effect size: Dietary quality	0.04	0.01423	0.012	0.068	Beta
BMI z-scores effect size: Physical activity	0.04	0.02	0.001	0.079	Beta
BMI z-scores effect size: Dietary quality	0.02	0.01	0.000	0.040	Beta
Prop. meeting PA guidelines control	0.13	0.0013	0.127	0.133	Beta
Prop. meeting PA guidelines intervention	0.16	0.049	0.117	0.257	Beta
Increase in Moderate to Vigorous PA	4.84	2.944	-0.940	10.610	Beta
**Relative effectiveness**					
OR for depression for Low vs High Physical Activity	0.83	0.026	0.790	0.880	Beta
OR of diabetes increasing the risk of depression	1.33	0.133	1.069	1.591	Lognormal
RR of PA on incidence of diabetes	0.74	0.074	0.595	0.885	Lognormal
**Mortality**					
Mental health: risk of death by suicide	0.001	0.000	0.001	0.001	Beta
HR of mortality those with type 2 Diabetes aged <65 years	2.978	0.190	2.605	3.350	Lognormal
HR of mortality those with type 2 Diabetes aged >65 years	1.958	0.050	1.860	2.056	Lognormal
**Relative effectiveness[a]**					
Pre-term birth relative risk for BMI P1	0.051	0.005	0.041	0.061	Beta
Pre-term birth relative risk for BMI P2	-0.171		0.111	-0.322	
MH BMI relative risk Boys P1	0.013	0.004	0.004	0.021	Beta
MH BMI relative risk Boys P2	0.727		0.914	0.540	
MH BMI relative risk girls P1	0.039	0.004	0.031	0.047	Beta
MH BMI relative risk girls P2	0.109		0.324	-0.005	
Diabetes relative risk boys P1	0.081	0.004	0.073	0.089	Beta
Diabetes relative risk boys P2	-0.845		-0.571	-0.912	
Diabetes relative risk girls P1	0.064	0.013	0.039	0.089	Beta

For peer review only - http://bmjopen.bmj.com/site/about/guidelines.xhtml

Parameter	Value	SD/SE	95% CI-Low	95% CI-High	Distribution
Diabetes relative risk girls P2	-0.314		0.167	-0.906	
Loss of earnings Men P1	12036	601.8	10856	13215.5	Gamma
Loss of earnings Men P2	-284478		-256213	-311886.5	
Loss of earnings Women P1	16689	1668.9	13418	19960.0	Gamma
Loss of earnings Women P2	-412424		-339474	-504989.1	
**Z scores [a]**					
Pre-term birth relative risk for BMI z-scores P1	0.1105	0.011	0.089	0.132	Beta
Pre-term birth relative risk for BMI z-scores P2	0.9336		-0.910	-1.841	
MH BMI z-scores relative risk Boys P1	0.0274	0.003	0.022	0.033	Beta
MH BMI z-scores relative risk Boys P2	1.0011		0.526	0.295	
MH BMI z-scores relative risk girls P1	0.084	0.008	0.068	0.100	Beta
MH BMI z-scores relative risk girls P2	0.9486		-0.452	-1.160	
Diabetes relative risk boys P1	0.1742	0.009	0.157	0.191	Beta
Diabetes relative risk boys P2	0.8964		-2.378	-3.112	
Diabetes relative risk girls P1	0.1369	0.027	0.083	0.191	Beta
Diabetes relative risk girls P2	1.0556		-0.790	-3.097	
**Proportions of population with mental health conditions**					
Chronic	0.170	0.017	0.137	0.203	Dirichlet
intermittent	0.400	0.040	0.322	0.478	Dirichlet
New and one-off	0.430	0.043	0.346	0.514	Dirichlet

[a] Risk equation is in the form: Relative risk = P1*BMI + P2

For peer review only - http://bmjopen.bmj.com/site/about/guidelines.xhtml

2 APPENDIX B

A breakdown of the resources used for estimating the costs of the intervention i.e. LifeLab Plus is presented below. The intervention comprises of three components:

i. Life Lab
ii. Digital App
iii. Teachers' Support (Healthy Conversation skills)

The costs are estimated for one-year that is, for the trial duration. The assumptions related to resources-used are based on a discussion with KTW on 5th June 2019.

Table 5: Life Lab

Item	Number	Comment
Number of classes per year	50	
No of pupils in each class	25	Varies between 25-32; assumed 25 for the base case
Total pupils	1250	=50 classes x 25 pupils per class
No of schoolteachers in each class	1	
Total teachers	50	

Table 6: Staff Training for Life Lab and Healthy Conversation Skills

Item	Number	Comment
Lifelab staff / training day (days)	6	Each training day / Lifelab day consists of 6 staff days to run. 4 staff days to run training and preparation and 2 days of administration.
Teacher training		
No. of Training days	5	Each training day is for 10 teachers.
Lifelab Staff days	30	=5 training days x 6 staff days / training day
Lifelab		
Number of days	50	=50x1
Lifelab Staff days	300	=50 life lab days x 6 staff day / lifelab
Scientists		Each lifelab day consists of 3 scientists
Number trained	75	Assumed each scientist does two lifelab days
Training days	3	=75 scientists /25; 25 trained per day
Lifelab Staff days	18	=3 training days x 6 staff days / training day
Training the trainers (Healthy Conversation Skills)		
Trainers	6	Assumption
Lifelab Staff days	1	Assume takes 2 hours
Showcase		All schools
Lifelab Staff days	3.5	1.5 days prep; 1 day delivery for 2 staff
Total lifelab staff days	352.5	Band 4 teacher

For peer review only - http://bmjopen.bmj.com/site/about/guidelines.xhtml

Data collection – Lifelab

Each class has two hours of data collection by two Lifelab staffs, 2 research nurses and 1 research assistant.

Table 7: Costs for data collection

Item	Number	Comment
Lifelab staff days	28.6	=2 hours x2 staff x50 (lifelab days)/7 (hours)
Research nurse	28.6	=2 hours x2 staff x50 (lifelab days)/7 (hours)
Research assistant	14.3	=2 hours x1 staff x50 (lifelab days)/7 (hours)

Table 8: Staff unit costs

Staff costs	Salary	Comment
Band 4 teacher	£34,189	i.e. £163 per day, Assumes 210 working days per year
Research assistant	£27,025	i.e. £129 per day, Assumes 210 working days per year
Research nurse Band 5	£26,231	i.e £125 per day, Assumes 210 working days per year

Table 9: Staff total costs

Staff costs	Total	Comment
Lifelab / training	£71,736	=£34,189/210x352.5*1.25;<br>= salary / working days per year*lifelab staff days*NI and pension adjustment<br>Assumes 210 working days per year, pension / NI contributions of 25%
Follow-up data collection	£12,641	=(£34,189*28.6 + £27,025 *28.6 + 26,231 *14.3)/210*1.25<br>= (salary teacher* data collection days teacher + salary research nurse* data collection days research nurse + salary research assistant*data collection days research assistant)/working days per year*NI / pension adjustment
Total	£84,377	

Table 10: Digital App development

Item	Total	Comment
Development of app	£324,000	Estimate from expert
Duration of development, year	5	As above
Duration of app, years	10	Assumption
Number of centres using app	10	Assumption
Cost per year development	£64,800	£324,000 / 5; total cost / number of years of development
Maintenance cost	£16,200	Assumed to be 25% of development cost
Cost per year of development across app lifetime per centre	£3240	£324,000 / 100; total development cost / (number of years app lasts for x number of centres)
EACH-B development for new centre	£87,445	Assumed to be 1 year of staff costs.

For peer review only - http://bmjopen.bmj.com/site/about/guidelines.xhtml

Item	Total	Comment
Number of years centre lasts	10	Assumption
Cost per year of centre	£8745	£87,445 / 10

Table 11: Summary of included costs

Item	Total	Comment
Staff costs	£84,377	
Room hire	£26,857	=400 (room hire cost)*67.14 (number of days); assumes cost of hire is £400 / day
Consumables	£15,839	Consumables include costs associated with printing, paper, classroom
Overheads	£37,970	Direct overheads based on 29% of direct care salary costs and indirect overheads based on 16% direct care salary costs (As used in Curtis et al 2019 for overheads for healthcare professionals)
Maintenance of app	£16,200	See above table
Capital cost, development of app	£3240	See above
Capital cost, development of EACH-B	£8745	See above

Table 12: Total costs

Item	Total	Comment
Total cost	£193,228	Sum of all of the above costs
Cost per pupil	£154.58	=£193,228/1250; (total cost /total number of pupils)

For peer review only

3 APPENDIX C

Figure 1: Cost-Effectiveness Acceptability Curve (CEAC)

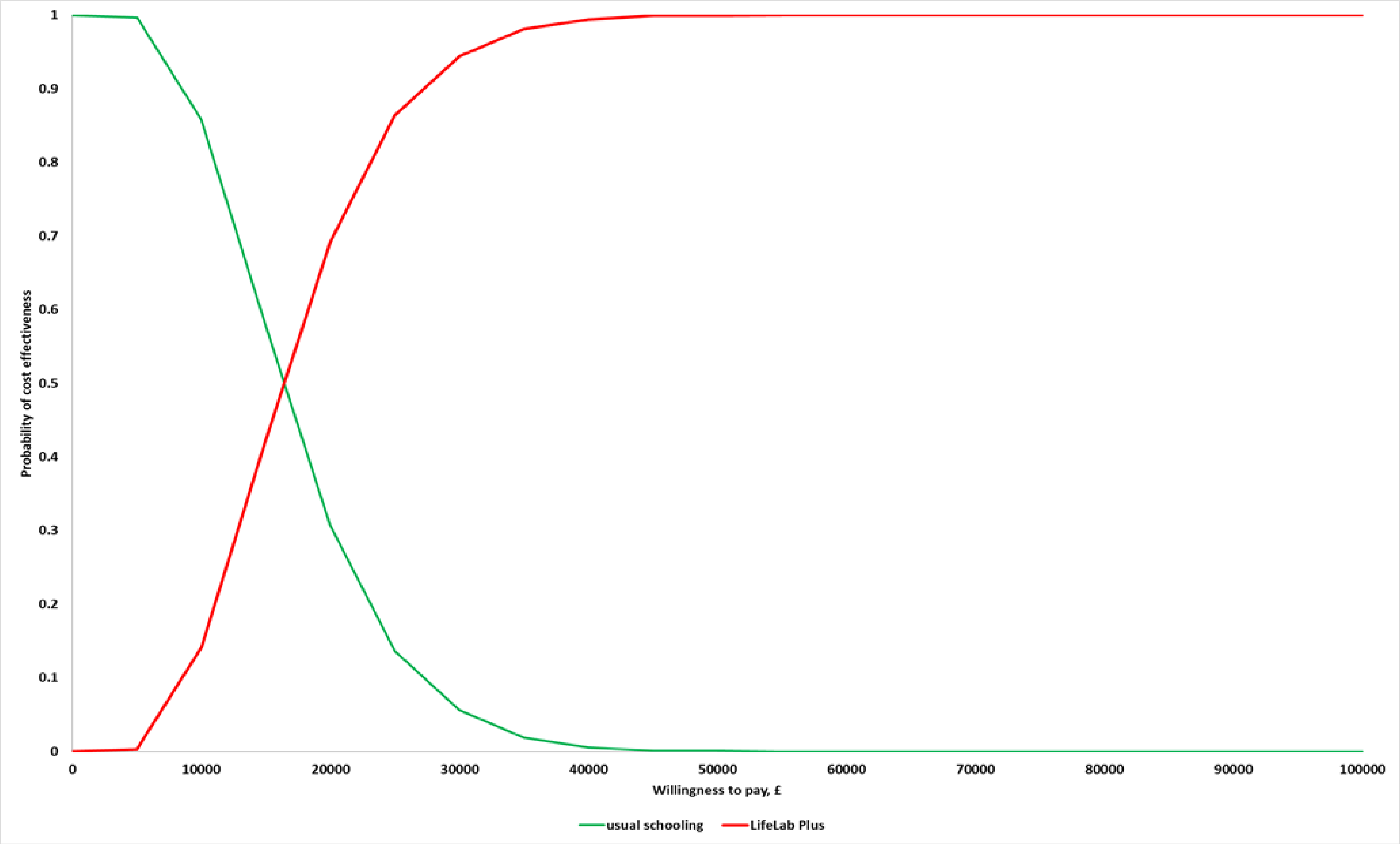

For peer review only - http://bmjopen.bmj.com/site/about/guidelines.xhtml

REFERENCES

1. NHS Digital. Health Survey for England 2016. 2017.
2. Sharma M, Nazareth I, Petersen I. Trends in incidence, prevalence and prescribing in type 2 diabetes mellitus between 2000 and 2013 in primary care: a retrospective cohort study. *BMJ Open* 2016;6(1):e010210. doi: 10.1136/bmjopen-2015-010210 [published Online First: 2016/01/16]
3. Gray N, Picone G, Sloan F, et al. Relation between BMI and diabetes mellitus and its complications among US older adults. *South Med J* 2015;108(1):29-36. doi: 10.14423/SMJ.0000000000000214 [published Online First: 2015/01/13]
4. McManus S BP, Jenkins R, Brugha T. Mental health and wellbeing in England: Adult Psychiatric Morbidity Survey 2014. 2016.
5. Sutaria S, Devakumar D, Yasuda SS, et al. Is obesity associated with depression in children? Systematic review and meta-analysis. *Arch Dis Child* 2019;104(1):64-74. doi: 10.1136/archdischild-2017-314608 [published Online First: 2018/07/01]
6. Chireh B, Li M, D'Arcy C. Diabetes increases the risk of depression: A systematic review, meta-analysis and estimates of population attributable fractions based on prospective studies. *Prev Med Rep* 2019;14:100822. doi: 10.1016/j.pmedr.2019.100822 [published Online First: 2019/03/01]
7. Schuch FB, Vancampfort D, Richards J, et al. Exercise as a treatment for depression: A meta-analysis adjusting for publication bias. *J Psychiatr Res* 2016;77:42-51. doi: 10.1016/j.jpsychires.2016.02.023 [published Online First: 2016/03/16]
8. Office of National Statistics. Births in England and Wales: 2017. 2018.
9. McDonald SD, Han Z, Mulla S, et al. Overweight and obesity in mothers and risk of preterm birth and low birth weight infants: systematic review and meta-analyses. *BMJ* 2010;341:c3428. doi: 10.1136/bmj.c3428 [published Online First: 2010/07/22]
10. Hamilton D, Dee A, Perry IJ. The lifetime costs of overweight and obesity in childhood and adolescence: a systematic review. *Obes Rev* 2018;19(4):452-63. doi: 10.1111/obr.12649 [published Online First: 2017/12/23]
11. Maheswaran H, Petrou S, Rees K, et al. Estimating EQ-5D utility values for major health behavioural risk factors in England. *J Epidemiol Community Health* 2013;67(2):172-80. doi: 10.1136/jech-2012-201019 [published Online First: 2012/07/31]

For peer review only

For peer review only - http://bmjopen.bmj.com/site/about/guidelines.xhtml

CHEERS checklist—Items to include when reporting economic evaluations of health interventions

Section/item	Item No	Recommendation	Reported on page No/ line No
**Title and abstract**			
Title	1	Identify the study as an economic evaluation or use more specific terms such as “cost-effectiveness analysis”, and describe the interventions compared.	page 1, line 1
Abstract	2	Provide a structured summary of objectives, perspective, setting, methods (including study design and inputs), results (including base case and uncertainty analyses), and conclusions.	page 2, lines 29 to 54
**Introduction**			
Background and objectives	3	Provide an explicit statement of the broader context for the study.	page 4, line 110 to page 5, line 137
		Present the study question and its relevance for health policy or practice decisions.	page 5, line 139 to 149
**Methods**			
Target population and subgroups	4	Describe characteristics of the base case population and subgroups analysed, including why they were chosen.	page 6, line 172 to 175
Setting and location	5	State relevant aspects of the system(s) in which the decision(s) need(s) to be made.	page 4, line 125 to page 5, line 137 and line 145 to line 149;
Study perspective	6	Describe the perspective of the study and relate this to the costs being evaluated.	page 11, line 347to 21; page 14, line 435;
Comparators	7	Describe the interventions or strategies being compared and state why they were chosen.	page 5, line 139to 149; page 6, line 174 to 175
Time horizon	8	State the time horizon(s) over which costs and consequences are being evaluated and say why appropriate.	page 7, line 214 to line 217 and line 222
Discount rate	9	Report the choice of discount rate(s) used for costs and outcomes and say why appropriate.	page 11, line 348
Choice of health outcomes	10	Describe what outcomes were used as the measure(s) of benefit in the evaluation and their relevance for the type of analysis performed.	page 7, line 201 to line 207
Measurement of effectiveness	11a	*Single study-based estimates:* Describe fully the design features of the single effectiveness study and why the single study was a sufficient source of clinical effectiveness data.	not applicable
	11b	*Synthesis-based estimates*: Describe fully the methods used for identification of included studies and synthesis of clinical effectiveness data.	page 8, line 229 to page 10, line 304; supplement appendix A
Measurement and valuation of preference based outcomes	12	If applicable, describe the population and methods used to elicit preferences for outcomes.	not applicable
Estimating resources and costs	13a	*Single study-based economic evaluation:*Describe approaches used to estimate resource use associated with the alternative interventions. Describe primary or secondary research methods for valuing each resource item in terms of its unit cost. Describe any adjustments made to approximate to opportunity costs.	not applicable

For peer review only

Section/item	Item No	Recommendation	Reported on page No/ line No
	13b	*Model-based economic evaluation:* Describe approaches and data sources used to estimate resource use associated with model health states. Describe primary or secondary research methods for valuing each resource item in terms of its unit cost. Describe any adjustments made to approximate to opportunity costs.	page 10, line 307 to page 11, line 337; pages 17-18, table 1; supplement Appendix B
Currency, price date, and conversion	14	Report the dates of the estimated resource quantities and unit costs. Describe methods for adjusting estimated unit costs to the year of reported costs if necessary. Describe methods for converting costs into a common currency base and the exchange rate.	page 10, line 322 to 323; page 11, line 335 to 337;
Choice of model	15	Describe and give reasons for the specific type of decision-analytical model used. Providing a figure to show model structure is strongly recommended.	page 5, line 153 to page 6, line 162; figure 1.
Assumptions	16	Describe all structural or other assumptions underpinning the decision-analytical model.	Page 6, line 159 to 160; Page 6, line 181 to 182; Page 7, line 210 to 211 and line 216 to 220; Page 8, line 242 to 244, 247 to 250, 253 to 254; Page 9, line 261 to 262, line 266 to 271, line 279 to 281, 291 to page 10 line 292; Page 10, line 294 to 304; Page 10, line 310 to 314; Page 11, line 326 to line 332; Page 12, line 361 to line 365; Page 13, line 403 to 404; Page 14, line 427 to 430, line 439 to 441.
Analytical methods	17	Describe all analytical methods supporting the evaluation. This could include methods for dealing with skewed, missing, or censored data; extrapolation methods; methods for pooling data; approaches to validate or make adjustments (such as half cycle corrections) to a model; and methods for handling population heterogeneity and uncertainty.	Page 11, line 340 to 343; page 11, line 356 to page12, line 367; ; Supplement Appendix A, Table 4
**Results**			
Study parameters	18	Report the values, ranges, references, and, if used, probability distributions for all parameters. Report reasons or sources for distributions used to represent uncertainty where appropriate. Providing a table to show the input values is strongly recommended.	Page 7, line 209 to page 12, line 367;; Pages 17 to 18, table 1; supplement Appendix A, Table 1 to 4.
Incremental costs and outcomes	19	For each intervention, report mean values for the main categories of estimated costs and outcomes of interest, as well as mean differences between the comparator groups. If applicable, report incremental cost-effectiveness ratios.	page 12, line 379 to 383; pages 19 to 20, table 2
Characterising uncertainty	20a	*Single study-based economic evaluation:*Describe the effects of sampling uncertainty for the estimated	not applicable

For peer review only

Section/item	Item No	Recommendation	Reported on page No/ line No
		incremental cost and incremental effectiveness parameters, together with the impact of methodological assumptions (such as discount rate, study perspective).	
	20b	*Model-based economic evaluation:* Describe the effects on the results of uncertainty for all input parameters, and uncertainty related to the structure of the model and assumptions.	page 12, line 386 to page 13, line 396; figures 2 and 3; pages 19 to 20, table 2; supplement Appendix C figure 1
Characterising heterogeneity	21	If applicable, report differences in costs, outcomes, or cost-effectiveness that can be explained by variations between subgroups of patients with different baseline characteristics or other observed variability in effects that are not reducible by more information.	not applicable
**Discussion**			
Study findings, limitations, generalisability, and current knowledge	22	Summarise key study findings and describe how they support the conclusions reached. Discuss limitations and the generalisability of the findings and how the findings fit with current knowledge.	page 13, line 399 to page 15, line 473
**Other**			
Source of funding	23	Describe how the study was funded and the role of the funder in the identification, design, conduct, and reporting of the analysis. Describe other non-monetary sources of support.	page 16, line 500 to line 506
Conflicts of interest	24	Describe any potential for conflict of interest of study contributors in accordance with journal policy. In the absence of a journal policy, we recommend authors comply with International Committee of Medical Journal Editors recommendations.	page 15, line 508 to line 511

For consistency, the CHEERS statement checklist format is based on the format of the CONSORT statement checklist

For peer review only

BMJ Open

Cost-effectiveness of a dietary and physical activity intervention in adolescents: a prototype modelling study based on the Engaging Adolescents in Changing Behaviour (EACH-B) programme

Journal:	*BMJ Open*
Manuscript ID	bmjopen-2021-052611.R2
Article Type:	Original research
Date Submitted by the Author:	11-May-2022

Complete List of Authors:	Kalita, Neelam; University of Southampton, Southampton Health Technology Assessments Centre<br>Cooper, Keith; Univ Southampton, SHTAC<br>Baird, Janis; University of Southampton, MRC Epidemiology Resource Centre; University of Southampton, NIHR Southampton Biomedical Research Centre<br>Woods-Townsend, Kathryn ; University of Southampton, Southampton Education School, Faculty of Social Sciences; University of Southampton , NIHR Southampton Biomedical Research Centre<br>Godfrey, Keith; University of Southampton and Southampton University Hospitals NHS Trust, MRC Lifecourse Epidemiology Unit; NIHR Southampton Biomedical Research Centre<br>Cooper, Cyrus; University of Southampton and Southampton University Hospitals NHS Trust, MRC Lifecourse Epidemiology Unit; University of Oxford, Nuffield Department of Orthopaedics, Rheumatology and Musculoskeletal Sciences<br>Inskip, Hazel; Medical Research Council, Epidemiology Resource Centre; NIHR Southampton Biomedical Research Centre<br>Barker, Mary; University of Southampton, MRC Lifecourse Epidemiology Unit; NIHR Southampton Biomedical Research Centre<br>Lord, Joanne; University of Southampton, Southampton Health Technology Assessments Centre

**Primary Subject Heading**:	Health economics
Secondary Subject Heading:	Diabetes and endocrinology, Health economics, Mental health, Nutrition and metabolism, Paediatrics
Keywords:	HEALTH ECONOMICS, MENTAL HEALTH, NUTRITION & DIETETICS, General diabetes < DIABETES & ENDOCRINOLOGY, Public health < INFECTIOUS DISEASES

SCHOLARONE™
Manuscripts

I, the Submitting Author has the right to grant and does grant on behalf of all authors of the Work (as defined in the below author licence), an exclusive licence and/or a non-exclusive licence for contributions from authors who are: i) UK Crown employees; ii) where BMJ has agreed a CC-BY licence shall apply, and/or iii) in accordance with the terms applicable for US Federal Government officers or employees acting as part of their official duties; on a worldwide, perpetual, irrevocable, royalty-free basis to BMJ Publishing Group Ltd ("BMJ") its licensees and where the relevant Journal is co-owned by BMJ to the co-owners of the Journal, to publish the Work in this journal and any other BMJ products and to exploit all rights, as set out in our licence.

The Submitting Author accepts and understands that any supply made under these terms is made by BMJ to the Submitting Author unless you are acting as an employee on behalf of your employer or a postgraduate student of an affiliated institution which is paying any applicable article publishing charge ("APC") for Open Access articles. Where the Submitting Author wishes to make the Work available on an Open Access basis (and intends to pay the relevant APC), the terms of reuse of such Open Access shall be governed by a Creative Commons licence – details of these licences and which Creative Commons licence will apply to this Work are set out in our licence referred to above.

Other than as permitted in any relevant BMJ Author's Self Archiving Policies, I confirm this Work has not been accepted for publication elsewhere, is not being considered for publication elsewhere and does not duplicate material already published. I confirm all authors consent to publication of this Work and authorise the granting of this licence.

For peer review only

Cost-effectiveness of a dietary and physical activity intervention in adolescents: a prototype modelling study based on the Engaging Adolescents in Changing Behaviour (EACH-B) programme

Authors: Neelam Kalita[1], Keith Cooper[1], Janis Baird[2;3], Kathryn Woods-Townsend[3;4], Keith M Godfrey[2;3], Cyrus Cooper[2;6;7], Hazel Inskip[2;3], Mary Barker[2;3;5], Joanne Lord[1] and the EACH-B study group

Affiliations:

[1] Southampton Health Technology Assessments Centre, University of Southampton, UK

[2] MRC Lifecourse Epidemiology Unit, University of Southampton, Southampton, UK

[3] NIHR Southampton Biomedical Research Centre, University of Southampton and University Hospital Southampton NHS Foundation Trust, Southampton, UK

[4] Southampton Education School, Faculty of Social Sciences, University of Southampton, Southampton, UK

[5] School of Health Sciences, University of Southampton, Southampton, UK

[6] Faculty of Medicine, University of Southampton

[7] Nuffield Department of Orthopaedics, Rheumatology and Musculoskeletal Sciences, University of Oxford

Corresponding author: Neelam Kalita

Email: N.Kalita@soton.ac.uk

For peer review only

ABSTRACT

Objective To assess costs, health outcomes and cost-effectiveness of interventions that aim to improve quality of diet and level of physical activity in adolescents.

Design A Markov model was developed to assess four potential benefits of healthy behaviour for adolescents: better mental health (episodes of depression and generalised anxiety disorder), higher earnings and reduced incidence of Type 2 diabetes and adverse pregnancy outcomes (in terms of pre-term delivery). The model parameters were informed by published literature. The analysis took a societal perspective over a 20-year period. One-way and probabilistic sensitivity analyses for 10,000 simulations were conducted.

Participants A hypothetical cohort of 100 adolescents with a mean age of 13 years.

Interventions An exemplar school-based, multi-component intervention that was developed by the Engaging Adolescents for Changing Behaviour programme, compared with usual schooling.

Outcome measure Incremental cost-effectiveness ratio as measured by cost per quality-adjusted life year (QALY) gained.

Results The exemplar dietary and physical activity intervention was associated with an incremental cost of £123 per adolescent and better health outcomes with a mean QALY gain of 0.0085 compared with usual schooling, resulting in an ICER of £14,367 per QALY. The results suggested that such an intervention has the potential to offer a cost-effective use of healthcare-resources for adolescents in the UK at a willingness-to-pay threshold of £20,000 per QALY. The key model drivers are the intervention effect on levels of physical activity, quality-of-life gain for high levels of physical activity, the duration of the intervention effects and the period over which effects wane.

Conclusions The model focused on short to medium-term benefits of healthy eating and physical activity exploiting the strong evidence base that exists for this age group. Other benefits in later life, such as reduced cardiovascular risk, are more sensitive to assumptions about the persistence of behavioural change and discounting.

For peer review only

ARTICLE SUMMARY

Strengths and limitations of this study

- The model was developed, based on currently available evidence, to assess the costs and health benefits of an exemplar school-based, multi-component intervention to improve adolescents' diet and increase their levels of physical activity.
- The economic evaluation followed published best-practice guidance.
- In the absence of effectiveness results from the on-going EACH-B trial, the treatment effect of the intervention is obtained from a published systematic review.
- The model does not include long-term health outcomes, such as for cardiovascular diseases or different types of cancer.
- There remains uncertainty around key model assumptions related to the duration of benefits of the intervention.

Mesh terms: Adolescent, Cost-effectiveness, School-based, Exercise, Diet

For peer review only - http://bmjopen.bmj.com/site/about/guidelines.xhtml

INTRODUCTION

Poor diet and lack of physical activity increase the risk of non-communicable diseases (NCDs), including cardiovascular diseases, Type 2 diabetes and some cancers such as breast, colon and endometrial, in part by contributing to overweight and obesity.[1 2] Adolescence, the life stage between childhood and adulthood, is a critical period for the development of health and disease in later life.[3 4] Compared with other age-groups, adolescents have the unhealthiest diets and most (over 80%) fail to meet national guidelines for physical activity.[5-7] Furthermore, the proportion meeting recommended levels of physical activity has been declining, particularly among girls (from 14% to 8%, 2008-2012).[8]

The disease burden of poor diet and physical inactivity on healthcare services is significant. In the UK, poor diet, and physical inactivity cost £7 billion to the National Health Service (NHS) annually.[9] Meeting current dietary recommendations would reduce years-of-life lost to coronary heart disease by 2 million, stroke by 400,000 and Type 2 diabetes by 19,000 over 20 years.[10]

Health behaviours in adolescence track into adulthood.[5 9 11 12] Therefore, sub-optimal diet and body composition in adolescence not only affect immediate physical and mental health but also increase the risk of NCDs in later life. Developmental plasticity in adolescence means that interventions to improve diet and levels of physical activity have the potential to reduce the trajectory of NCD risk over the life course.[13] While many adolescents find it difficult to engage with the long-term consequences of health behaviour, motivated and engaged adolescents can improve their health behaviours.[14 15] Evidence suggests that school-based interventions that offer combinations of peer-modelling, social support and choice, may be effective in improving diet and physical activity amongst adolescents.[16 17 18] Furthermore there is an increasing use of digital platforms by adolescents. According to 2018 estimates, 83% of 12-15 years olds in the UK own smartphones, with 99% spending an average of 20 hours per week online.[19 20] With an explosion in the use of such platforms to influence health behaviours in young people, they have potential as a complementary feature in complex interventions that aim to influence health behaviour in adolescents.[20]

Within this framework, a research programme Engaging Adolescents in CHanging Behaviour

For peer review only - http://bmjopen.bmj.com/site/about/guidelines.xhtml

(EACH-B) was designed to develop and test an intervention to encourage UK-based school students, aged 12-13 years, to adopt healthy behaviours such as eating better and exercising more (Trial registration: ISRCTN 74109264). EACH-B involves a cluster randomised controlled trial as a test of intervention effectiveness. Further details of the trial design are given elsewhere.[20] The research programme is funded by the National Institute for Health Research (RP-PG-0216-20004). The "LifeLab Plus" intervention developed as part of this programme is a complex three-part programme that comprises: i) an education module that teaches school students the science behind health messages through a 2-week module with a "hands-on" practical one-day visit to a teaching laboratory at University Hospital Southampton or in school while COVID-19 restrictions apply; ii) training for teachers in skills to support behaviour change; and iii) access to a specially-designed, interactive smartphone app with game features.

There is an emerging interest in identifying and developing interventions for improving diet and physical activity levels in adolescents. Taking this into consideration, we developed an illustrative decision-analytic model to assess the health benefits, costs, and cost-effectiveness of a multi-component intervention such as LifeLab Plus. This prototype model is designed to investigate how changes in diet quality and physical activity could affect future health outcomes and costs. We used cost data from the EACH-B programme and effectiveness estimates for similar interventions from published literature. The aims of this paper are to describe the structure, assumptions and parameters for our prototype model, to present preliminary (prior) estimates of cost-effectiveness based on currently available information and explore the sensitivity of results to key uncertainties. The model will be updated when results from the EACH-B trial are available.

METHODS

We developed a prototype *de novo* Markov model to estimate the costs, benefits and cost-effectiveness of school-based interventions that aim to improve diet quality and levels of physical activity compared with usual schooling for a cohort of adolescents. The model focussed on four potential short to medium-term benefits of healthy eating and physical activity: better mental health (episodes of depression and generalised anxiety disorder), higher earnings and reduced incidence of Type 2 diabetes and adverse pregnancy outcomes

For peer review only - http://bmjopen.bmj.com/site/about/guidelines.xhtml

(in terms of pre-term delivery). The model assumed that improved diet quality and increased physical activity would impact these four aspects of health via a reduction in BMI. Discussion with key project stakeholders reiterated these four benefits as the most relevant obesity-related effects in this population.

The model did not include later life impacts on cardiovascular disease or other chronic diseases as the impact on these outcomes of an intervention undertaken as an adolescent is uncertain. The model also investigated independent effects of physical activity on diabetes and depression (i.e. direct impacts not mediated by BMI). Information relating to epidemiology, mortality, effectiveness, health-related quality-of-life and costs was obtained from a variety of sources to inform the model parameters and assumptions.

Structuring the model

Population

A cohort of 100 adolescents with an equal proportion of boys and girls and a mean age of 13 years entered the model. The exemplar intervention is based on LifeLab Plus[20] and compared with usual schooling.

Model states

The Markov model consisted of three health states as described below (Figure 1). Outcomes associated with mental health, loss of earnings and adverse pregnancy outcome were incorporated as model events.

- *Healthy*: Adolescents enter the model in this state where they are assumed to have no other comorbidities including Type 2 diabetes or mental health problems. In each model cycle, a proportion will develop Type 2 diabetes and move to the *'Diabetes'* state or they may experience- mental health events, -pre-term births (girls only) or -loss of earnings. The latter three are treated as transient states.
- *Diabetes:* A proportion of adolescents with Type 2 diabetes may enter the transient states, as stated above.
- *Death:* This is an absorbing state. Adolescents may transition to this state either through general population mortality or death from- mental health conditions or -Type 2 diabetes and related complications.

For peer review only - http://bmjopen.bmj.com/site/about/guidelines.xhtml

For peer review only

Mental health encompasses a wide spectrum of conditions. Therefore, a pragmatic approach was adopted to include the two most prevalent mental health conditions for adolescents: clinical depression (henceforth, referred as depression) and General Anxiety Disorder (GAD). These conditions were categorised as: chronic (history of persistent mental illness), intermittent (experiencing intermittent episodes), and new onset (a one-time episode). Adverse pregnancy outcome was defined by pre-term delivery categorised as: extremely pre-term (delivery <28 weeks); very pre-term (delivery <33 weeks); and moderately pre-term (delivery between 34-36 weeks). The pregnancy outcome was applicable for girls only.

Each health state and event are associated with an impact on health-related quality-of-life (HRQoL) and excess cost. The model included intervention cost associated with LifeLab Plus and healthcare costs associated with- Type 2 diabetes, mental health events, pre-term delivery and loss of earnings due to obesity. In each model cycle, the total costs and QALYs are calculated by multiplying the individual costs and HRQoL by the number of people in the cohort still alive for each of the intervention and control arms. The total lifetime costs and QALYs are calculated by aggregating the costs and QALYs for all cycles.

Persistence of effects and time horizon

Adolescents were assumed to receive the intervention at the model entry, the duration of which lasted for a year. Although there is good epidemiological evidence of long-term tracking of health behaviour,[5,9,11,12] school-based trials rarely follow up for more than a year.[1,21,22] The persistence of intervention effect in terms of behaviour change is therefore uncertain. Hence, we take a conservative approach and focus on potential impacts of improved diet and physical activity from such an intervention likely to manifest in the short to medium term: up to a maximum time horizon of 20 years. However, due to the uncertainty, the treatment effect on behaviour was not assumed to last for 20 years. Based on our experts' judgement, for the base case the effect was assumed to sustain for 4 years, waning to no effect over a period of 10 years. We explored alternative assumptions about the persistence of effects on behaviour after trial follow-up.

The model time horizon was 20 years with a yearly cycle length. The time horizon was considered appropriate as the mean age of adolescents entering the model was 13 years as in the EACH-B trial. Costs and outcomes were half-cycle corrected.

For peer review only

For peer review only - http://bmjopen.bmj.com/site/about/guidelines.xhtml

Populating the model

Targeted literature searches were conducted to identify sources to inform model parameters. These are discussed below and in Appendices A and B.

Epidemiological data

Data on the relationship between mean BMI, age and sex was taken from Health Survey for England.[23] For adolescents aged ≤19 years, BMI z-scores are normally used. Therefore, we rescaled the values to relate relative risks to BMI z-scores, where:

BMI z-score = (observed value – median value of the reference population) / standard deviation value of reference population.

Physical activity levels for children aged 13-15 years were taken from Health Survey England, 2015.[24] The incidence of Type 2 diabetes in the UK was based on an analysis of longitudinal electronic health records in the Health Improvement Network (THIN) primary care database.[25] The prevalence of depressive episodes and GAD was taken from the Adult Psychiatric Morbidity Survey 2014[26] and from Mental Health of Children and Young people in England 2017.[27] The proportion in each category was assumed as follows: 17% had a chronic and 40% had a fluctuating (intermittent) course, while 43% remitted (new one-time episode).[28] Those individuals with depression or anxiety are at higher risk of suicide than the general population.[29] The excess death rate for those with depression and anxiety was calculated by using the suicide rate in the UK from Office of National Statistics (ONS) 2017 and applying a relative risk of 10.9 for depression and anxiety.[29] The proportions of pre-term deliveries, obtained from ONS 2017 data, were assumed as follows: 0.5% of total births as extremely pre-term (<28 weeks), 1.2% very pre-term birth (28 to <33 weeks) and 6.3% moderately pre-term birth (33-36 weeks) respectively.[30]

Relationship between BMI and risks of health events

The economic model assumed a positive correlation between increased BMI and the risks of - Type 2 diabetes, depression and GAD, pre-term delivery and loss of earnings. We fitted equations to the BMI relative risks. Hazard ratios, obtained from the Medicare Current Beneficiary Survey 1991-2010, were used to estimate the increased risk of individuals with higher BMI developing Type 2 diabetes.[31] The odds of depression and GAD in obese and

For peer review only - http://bmjopen.bmj.com/site/about/guidelines.xhtml

overweight adolescents compared with normal-weight adolescents were obtained from Sutaria et al.[32] This systematic review included 22 observational studies published between 2000 and 2017, representing 143,603 children. The relative risk of pre-term birth for mothers with overweight and obesity was obtained from Mcdonald et al.[33] This was assumed to be the same for all three categories of pre-term births. The loss of earnings which is applied to change in BMI over time is estimated annually over the model time horizon. It is scaled according to the population age to factor in average income by age.

Relationship between physical activity and risk of health event

The direct effect of physical activity on developing depression is modelled independent of the effect of physical activity via BMI. The odds ratio of developing depression was assumed to be 0.83 (95% CI 0.79-0.88) in those with high levels of physical activity compared to those with lower levels.[34] Furthermore, an increase from being inactive to achieving 150 minutes of moderate-intensity physical activity per week was assumed to lower the risk of Type 2 diabetes incidence by 26%, after adjustment for body weight.[35] The pooled odds ratio between Type 2 diabetes and risk of depression was 1.33 (95% CI, 1.18-1.51).[36]

Intervention effect

The intervention effect was based on three systematic reviews and meta-analyses that estimated the overall effects of school-based obesity prevention interventions.[37 38 39] The results of the meta-analyses were found to be significantly different between groups based on BMI (− 0.17 (95% CI− 0.29, − 0.06) kg/m^2)[38] and BMI z-score (− 0.06 (95% CI -0.10, − 0.03))[39] for multi-component interventions including physical activity, health education and dietary improvement. The effect of school-based intervention was assumed to increase the level of moderate or vigorous physical activity in children and adolescents by 4.84 min/day (95% CI −0.94 to 10.61).[40]

Mortality

General population mortality, adjusted for age and gender, was based on ONS 2020.[41] A hazard ratio of mortality of 2.98 was applied for individuals with Type 2 diabetes and aged between 35 and <65 years.[42]

Quality-of-life

For peer review only - http://bmjopen.bmj.com/site/about/guidelines.xhtml

EQ-5D-3L estimates adjusted for age, gender and BMI were used to estimate quality-of-life (Table 1). These estimates were obtained from 14,117 participants aged ≥16 years from the Health Survey for England 2008.[43] Adults with diabetes were assumed to have a disutility of -0.161.[44] We estimated the disutility by comparing the mean general population EQ-5D score with that for people with diabetes. A disutility of -0.087 is used for adolescents with Type 2 diabetes based on a Swedish cohort of adolescents aged 13-18 years.[45] A utility decrement of 0.188 was assumed for those with intermittent episodes of mental health conditions;[46] a decrement of 0.488 for those with persistent/chronic depression;[47] a decrement of 0.094 (half of the decrement for intermittent depression) was assumed for those with a new episode of depression. For pre-term delivery, a mean utility decrement of 0.066 was applied throughout the model time horizon. This was based on a systematic review and meta-analysis for health utility values associated with pre-term birth where all but one study used Health Utilities Index (HUI) Mark 2 (HUI2) or Mark 3 (HUI3) measures as their primary health utility assessment method.[48] We found no evidence for quality-of-life loss in parents of pre-term babies. Therefore, we assumed that the quality-of-life decrement would be like intermittent mental health condition and lasts for the first two years.

Costs

We describe the costs below and summarise in Table 1.

Intervention costs

The intervention cost for our illustrative analysis was based on LifeLab Plus. Further information on resources used for delivering LifeLab are in Appendix B. The cost of the app delivered as part of the intervention, was incorporated as a capital cost and was assumed to last 10 years and be used in 10 centres. Similarly, the cost of setting up LifeLab Plus in a different centre was assumed to- consist of one year's staff costs and last for 10 years. Maintenance costs were estimated at 25% of the development cost per year. Overheads were included according to the rates used in Curtis et al. 2019[49]: direct overheads based on 29% of direct care salary costs and indirect overheads based on 16% direct care salary costs.

Health state costs

Both direct and indirect costs relating to individuals with Type 2 diabetes,[50] depression,[51] pre-term birth,[52] and loss of earnings were included. Indirect costs included the effect of

For peer review only - http://bmjopen.bmj.com/site/about/guidelines.xhtml

depression on income and productivity. Costs were updated to 2019 prices using the hospital and community health services index.[49]

Direct health costs and indirect societal and productivity costs were estimated using a top-down approach. We assumed that individuals with Type 2 diabetes would not incur costs for complications as these are likely to affect individuals who have diabetes for a longer period. For costs associated with pre-term delivery, total societal costs for children born at 32-33 weeks and 34-36 weeks gestation from birth to 24 months were based on a study that compared these costs to those for children born at full-term.[53] The difference in costs for children born <28 weeks and 28-33 weeks were assumed to be same when compared to those born at full-term.

The lifetime indirect costs for overweight and obesity in childhood and adolescence is based on a study by Hamilton et al.[54] Mean total lifetime healthcare and productivity costs were estimated in Irish Euros, which were converted to GBP (£) and adjusted according to the average wage in the UK.

Validation

The structure of the prototype model was validated by the study team comprising epidemiologists, statisticians, trialists, public health experts and health economists. Internal validity of the model was established ensuring that the model predictions were consistent with the model specification.

Cost-effectiveness analysis

Costs and effects were estimated in accordance with the NICE Reference case,[55] except for the perspective adopted; we used a societal perspective that included both direct and indirect costs. Costs and health effects (QALYs) were discounted at 3.5% per year. The incremental cost-effectiveness ratio (ICER) was estimated as a ratio of the incremental costs of the intervention (LifeLab Plus) relative to the comparator (usual schooling) to the incremental QALYs of the intervention relative to the comparator. The intervention was considered cost-effective if the ICER was below the threshold of £20,000 per QALY gained, the lower threshold usually considered for the English NHS by NICE.[55]

Sensitivity analyses

For peer review only - http://bmjopen.bmj.com/site/about/guidelines.xhtml

To assess the uncertainty around the model predictions, we conducted deterministic sensitivity analysis (DSA), probabilistic sensitivity analyses (PSA) and scenario analyses. For the DSA, input parameters were varied by 95% confidence intervals (where available) or varied by 10% of the mean. Monte Carlo simulations of 10,000 iterations were run for the PSA to assess the combined effects of input parameter uncertainties where parameters were simultaneously sampled within a specified distribution. We used standard distributions for the PSA, as recommended by Briggs et al.[56]: effectiveness parameters were assigned beta and lognormal distributions; utilities were assumed to follow a beta distribution; and costs were assigned a gamma distribution (Table 1 and further details in Appendix A). Scenario analyses were conducted to assess structural uncertainties related to model assumptions. Uncertainty about the sustainability of the intervention effect was assessed by varying the duration of intervention effect and its waning period.

The model was developed and implemented in Microsoft Excel.

Patient and public involvement

The research questions addressed in the overarching research programme EACH-B were informed by public involvement. Furthermore, representatives from public involved in the EACH-B research programme were presented the conceptual framework, modelling approaches, and invited to comment.

RESULTS

Base case analysis

For the base case, the exemplar intervention based on the multi-component LifeLab Plus was associated with higher costs and better health outcomes (more QALYs) compared with usual schooling. LifeLab Plus was associated with a mean QALY gain of 0.0085 at an incremental cost of £123 per person compared with usual schooling, resulting in an ICER of £14,367 per QALY (Table 2).

Sensitivity analyses

The results of the one-way sensitivity analyses are presented in the Tornado plot (Figure 2). Parameters including the intervention effect on levels of physical activity (expressed in terms

For peer review only - http://bmjopen.bmj.com/site/about/guidelines.xhtml

of minutes of moderate or vigorous physical activity), quality-of-life gain for high levels of physical activity, duration of the intervention effect and duration of the treatment waning period had the highest impact on the cost-effectiveness results. Other parameters such as effect of physical activity on BMI, time horizon and intervention costs also influenced the base case results, but to a lesser extent. The results of the PSA are presented as a cost-effectiveness scatter plot in Figure 3. All the iterations lie in the North-East quadrant of the cost-effectiveness plane, thereby indicating that the intervention is likely to produce health benefits at an additional cost. At a willingness-to-pay threshold of £20,000 per QALY gained, the probability of LifeLab Plus being cost-effective was 69% compared with usual schooling (Appendix C).

DISCUSSION

Main findings

Based on our prototype economic model, we estimate that a multi-component intervention to improve dietary quality and physical activity, such as LifeLab Plus, is likely to be considered cost-effective under conventional willingness-to-pay thresholds of £20,000–£30,000 per QALY gained in the UK. For our base case, the duration of the treatment effect was assumed to sustain for 4 years with the effect waning over a further period of 10 years. Our sensitivity analyses showed that if the duration of the treatment effect was not sustained to this extent, the intervention would be less cost-effective (Table 2). Changes to physical activity had a greater effect on the model results than changes in BMI. This is largely because physical activity is associated with an additional improvement in quality-of-life, independent of any health benefits associated with the health states in our model.

Comparison with previous models

Previous cost-effectiveness studies evaluating dietary and physical activity interventions for adolescents have largely consisted of within-trial analyses that have not considered the benefits beyond the trial period.[57-61] Cost-effectiveness estimates vary between a cost saving of £408.22 (NZ$835) per child for a low intensity program[57] to an additional cost of £120,630 per QALY gained for a more intensive intervention.[60] However, comparison between studies is difficult because of the differences in- the study designs, the interventions considered and the outcomes reported.

For peer review only - http://bmjopen.bmj.com/site/about/guidelines.xhtml

Gc et al[1] developed a model to assess the long-term costs and health outcomes of two physical activity interventions targeting adolescents in the UK. The cost-effectiveness estimates varied from £11,426 per QALY for an after-school intervention to £68,056 per QALY for a multicomponent intervention. The costs of these interventions were estimated at £51 per participant for the after-school intervention and £207 (AUS$394) for the multicomponent intervention. Their model included different health states to our model for diseases which typically affect people in later life including chronic heart disease, stroke, heart failure, breast failure and colorectal cancer. They ran the model for a lifetime horizon of 65 years. We have not included these health states as we adopted a conservative assumption that treatment benefits for adolescents from such multicomponent intervention as LifeLab Plus do not persist beyond 20 years.

Strengths and limitations

The study addresses an important public health question by examining whether interventions targeting healthy eating and increased physical activity for adolescents provide value for money from a societal perspective. We incorporate existing evidence on the effect of improvement in adolescent health behaviours on four high prevalence short-to-medium term benefits relevant to adolescents: improved mental health, higher earnings, improved pregnancy outcomes and prevention of Type 2 diabetes. Sources for data used within our model were identified from a targeted literature review. Where data were not available for adolescents, we used data from adult population. Model structure and assumptions were informed by this review and discussions with public health experts. UK-specific incidence rates were used to ensure that patients entering the model matched the likely distribution of events in the UK. Our analysis focuses on a pragmatic subset of specific outcomes, while noting these could be expanded on with emerging data on effectiveness, notably in relation to mental health and other long-term conditions.

We do not attempt to estimate the long-term impacts of such interventions due to the lack of longitudinal data on lifetime trajectories of healthy diet and increased levels of physical activity. Our preliminary model suggests that this type of intervention is likely to be cost-effective based on short-to-medium term effects alone. If this is correct and the effects last longer, then additional benefits such as prevention of cardiovascular disease would enhance the cost-effectiveness of these interventions. This suggests that our cost-effectiveness results are conservative, and that quantification of long-term benefits is not necessary to demonstrate

For peer review only - http://bmjopen.bmj.com/site/about/guidelines.xhtml

the cost-effectiveness of this type of intervention. However, the sensitivity analyses reported above do indicate some important uncertainties.

Our model has several shortcomings. The EACH-B study, which is the main trial evidence, is ongoing so there is currently no direct data on the effectiveness of this intervention. We have used evidence on treatment effectiveness from a systematic review of related interventions. When results become available from the EACH-B study, they will add to the evidence base and reduce uncertainty in these results. Further evidence for other model parameters would also be valuable. For example, where data from adolescent age groups were not available, we used epidemiological estimates from adult populations that might not be transferable. There is uncertainty around key assumptions related to the duration of benefits from the intervention. In our analysis, we have assumed that the benefit observed in the clinical trials will last for 4 years and then will gradually reduce over the next 10 years. We only include costs and effects over a 20-year time horizon and have assumed that there would be no further benefits of the intervention for chronic diseases such as cardiovascular diseases and diabetes. There are also limitations in our approach to estimating income lost due to obesity. We have adopted a simple approach; however, this is a complex interacting bi-directional system. We have not fully explored whether income loss is due to obesity or whether obesity is caused by income loss through, for example, unemployment. Other broader uncertainties, such as long-term effects of COVID on behaviour and mental health are also not addressed.

CONCLUSION

Complex behavioural interventions that aim to improve diet and increase levels of physical activity amongst school-aged children have the potential to provide cost-effective use of UK healthcare resources. Such interventions have the potential to reduce burden of NCDs, although benefits in later life are more sensitive to assumptions about the persistence of behavioural change and discounting. Our analysis focuses on a pragmatic subset of specific outcomes based on published literature on effectiveness of similar interventions, while noting these could be expanded on with emerging data on effectiveness, notably in relation to mental health and other long-term conditions.

Word count: 4222

For peer review only

For peer review only - http://bmjopen.bmj.com/site/about/guidelines.xhtml

Author Contributions NK designed the model, performed the analysis, interpreted the analysis, and wrote the first draft of the manuscript. KC designed the model and performed the analysis. JL supervised the process, provided comments on model structure and data analysis. KWT provided inputs for model parameters. JB, KMG, HI, MB and CC provided critical comments on the model structure. All authors contributed to the critical revision of the manuscript and approved the final version of the manuscript.

Ethics approval Not required as the model is informed by data from the published literature.

Funding The research is funded by the National Institute for Health Research (RP-PG-0216-20004). HMI and KMG are funded by the UK Medical Research Council (MC_UU_12011/4). KMG is supported by the National Institute for Health Research (NIHR Senior Investigator (NF-SI-0515-10042), NIHR Southampton 1000DaysPlus Global Nutrition Research Group (17/63/154) and NIHR Southampton Biomedical Research Centre (IS-BRC-1215-20004)), the European Union (Erasmus+ Programme ImpENSA 598488-EPP-1-2018-1-DE-EPPKA2-CBHE-JP) and the British Heart Foundation (RG/15/17/3174).

Competing Interests KMG has received reimbursement for speaking at conferences sponsored by companies selling nutritional products and is part of an academic consortium that has received research funding from Abbott Nutrition, Nestec, BenevolentAI Bio Ltd. and Danone. The other authors have no potentially competing interests to declare.

Patient consent for publication Not required.

Data availability statement Data from publicly available sources were used for the model. All model inputs are described in the paper.

For peer review only

For peer review only - http://bmjopen.bmj.com/site/about/guidelines.xhtml

Table 1 Input parameters used in the model

**Parameter**	Value	SD/SE	95% CI-Low	95% CI-High	Distribution	Reference
**Utility inputs**						
Disutility for Diabetes for 13-18 years old	-0.087	0.036	0.016	0.158	Beta	[45]
Disutility for Diabetes	-0.161	0.040	0.240	0.080	Beta	[44]
Disutility for pre-term babies < 33 weeks	-0.066	0.016	0.098	0.035	Beta	[48]
Disutility for parents of pre-term babies < 33 weeks	-0.094	0.019	0.057	0.131	Beta	Assumption
Disutility for depression	-0.188	0.038	0.114	0.262	Beta	[46]
Disutility for Chronic mental health condition	-0.448	0.036	0.377	0.519	Beta	[47]
Disutility for Intermittent mental health condition	-0.188	0.038	0.114	0.262	Beta	[46]
Disutility for New cases mental health condition	-0.094	0.019	0.057	0.131	Beta	Assumption
Quality-of-life gain for high activity	0.020	0.002	0.016	0.024	Beta	
**Direct costs**						
Type 2 diabetes	£622	£62	£500	£744	Gamma	[50]
Mental health condition	£1,334	£383	£585	£2,307	Gamma	[51]
Mental health condition- chronic	£4,233	£629	£3,001	£7,238	Gamma	[51]

For peer review only

Mental health condition- intermittent	£1,334	£383	£585	£2,307	Gamma	[51]
Mental health condition-new onset	£667	£191	£292	£1,042	Gamma	[51]
Pre-term birth <28 weeks	£25,452	£2,169	£21,201	£29,704	Gamma	[52;53]
Pre-term birth 28-33 weeks	£13,073	£1,114	£10,889	£15,256	Gamma	[52;53]
Pre-term birth, 34-36 weeks	£4,157	£1,020	£2,157	£6,157	Gamma	[52;53]
**Indirect costs**						
Type 2 diabetes	£4,116	£412	£3,309	£4,923	Gamma	[50]
Mental health condition	£223	£64	£98	£348	Gamma	[51]
Mental health condition- chronic	£707	£203	£310	£1,105	Gamma	[51]
Mental health condition- intermittent	£223	£64	£98	£348	Gamma	[51]
Mental health condition-new onset	£112	£32	£49	£174	Gamma	[51]
Pre-term birth <28 weeks	£397	£114	£174	£619	Gamma	[52;53]
Pre-term birth 28-33 weeks	£119	£34	£52	£185	Gamma	[52;53]
Pre-term birth, 34-36 weeks	£103	£29	£45	£161	Gamma	[52;53]
**Cost of the intervention**	£155	£15	£124	£185	Gamma	Estimated

SD: Standard Deviation; SE: Standard Error; CI: Confidence Interval

For peer review only - http://bmjopen.bmj.com/site/about/guidelines.xhtml

Table 2 Incremental cost-effectiveness ratios in the base case and scenario analyses

	Total cost (£)	Total QALYs	Incremental cost (£)	Incremental QALY	ICER
**Base case analysis**					
Usual schooling	£4,416	13.07			
LifeLab Plus	£4,539	13.08	£123	0.0085	£14,367
**Perspective: NHS**					
Usual schooling	£2,001	13.07			
LifeLab Plus	£2,141	13.08	£140	0.0085	£16,339
**Costs discounted at 3.5%; QALYs at 1.5% per annum**					
Usual schooling	£4,416	15.76			
LifeLab Plus	£4,539	15.77	£123	0.0097	£12,640
**Costs and QALYs discounted at 1.5%**					
Usual schooling	£5,813	15.76			
LifeLab Plus	£5,930	15.77	£117	0.0097	£12,044
**Time Horizon: 5 years**					
Usual schooling	£488	4.25			
LifeLab Plus	£633	4.25	£145	0.0043	£33,423
**Time Horizon: 10 years**					
Usual schooling	£1,132	7.76			
LifeLab Plus	£1,265	7.77	£134	0.0071	£18,851
**Time Horizon: 30 years**					
Usual schooling	£11,137	16.67			
LifeLab Plus	£11,258	16.67	£121	0.0088	£13,719
**Time Horizon: 40 years**					
Usual schooling	£18,417	19.05			
LifeLab Plus	£18,537	19.06	£120	0.0089	£13,455
**Duration of treatment effect: 1 year & Treatment waning: 2 years**					
Usual schooling	£4,416	13.07			
LifeLab Plus	£4,566	13.08	£150	0.0030	£49,758
**Duration of treatment effect: 1 years & Treatment waning: 5 years**					
Usual schooling	£4,416	13.07			
LifeLab Plus	£4,563	13.08	£147	0.0043	£34,089
**Duration of treatment effect: 2 years & Treatment waning: 5 years**					
Usual schooling	£4,416	13.07			
LifeLab Plus	£4,560	13.08	£144	0.0051	£28,034
**Duration of treatment effect: 5 years & Treatment waning: 5 years**					
Usual schooling	£4,416	13.07			
LifeLab Plus	£4,553	13.08	£137	0.0075	£18,218
**Duration of treatment effect: 5 years & Treatment waning: 10 years**					
Usual schooling	£4,416	13.07			
LifeLab Plus	£4,529	13.08	£113	0.0092	£12,184
**Duration of treatment effect: 10 years & Treatment waning: 10 years**					
Usual schooling	£4,416	13.07			
LifeLab Plus	£4,432	13.09	£16	0.0125	£1,257
**Duration of treatment effect: 15 years & Treatment waning: 5 years**					
Usual schooling	£4,416	13.07			

For peer review only

Table 2 Incremental cost-effectiveness ratios in the base case and scenario analyses

	Total cost (£)	Total QALYs	Incremental cost (£)	Incremental QALY	ICER
LifeLab Plus	£4,308	13.09	-£108	0.0147	Dominates

ICER, incremental cost-effectiveness ratio (incremental cost/incremental QALY); QALY, quality-adjusted life year.

List of Figure legends

- Figure 1 provides an illustration of the conceptual framework for the cost-effective model and an illustration of the model structure that shows transition of the modelled population through the modelled health states.

- Figure 2 provides an illustration of the Tornado diagram obtained from the deterministic sensitivity analysis that shows the impact of varying the input values of the parameters that have the highest impact on the cost-effectiveness result.

- Figure 3 provides an illustration of the scatter plot obtained from the probabilistic sensitivity analysis. Results from all the 10,000 simulations fall within the North-East quadrant of the cost-effectiveness plan thereby indicating that the intervention (LifeLab Plus) is more expensive and more effective compared to usual schooling.

For peer review only

For peer review only - http://bmjopen.bmj.com/site/about/guidelines.xhtml

REFERENCES

1. Gc VS, Suhrcke M, Atkin AJ, et al. Cost-effectiveness of physical activity interventions in adolescents: model development and illustration using two exemplar interventions. *BMJ Open* 2019;9(8):e027566. doi: 10.1136/bmjopen-2018-027566 [published Online First: 2019/08/21]
2. Organisation WH. Unhealthy Diets & Physical Inactivity 2009 [Available from: https://www.who.int/nmh/publications/fact_sheet_diet_en.pdf accessed 19/11/2020.
3. Organisation WH. Adolescent Health 2020 [Available from: https://www.who.int/health-topics/adolescent-health/#tab=tab_1 accessed 13/11/20.
4. Viner RM, Ross D, Hardy R, et al. Life course epidemiology: recognising the importance of adolescence. *Journal of Epidemiology and Community Health* 2015;69(8):719-20. doi: 10.1136/jech-2014-205300
5. Rose T, Barker M, Maria Jacob C, et al. A Systematic Review of Digital Interventions for Improving the Diet and Physical Activity Behaviors of Adolescents. *J Adolesc Health* 2017;61(6):669-77. doi: 10.1016/j.jadohealth.2017.05.024 [published Online First: 2017/08/16]
6. Bates B, Lennox A, Prentice A, et al. National Diet and Nutrition Survey: Results from Years 1, 2, 3 and 4 (combined) of the Rolling Programme (2008/2009-2011/2012): a Survey Carried Out on Behalf of Public Health England and the Food Standards Agency: Public Health England, 2014.
7. Woods-Townsend K, Bagust L, Barker M, et al. Engaging teenagers in improving their health behaviours and increasing their interest in science (Evaluation of LifeLab Southampton): study protocol for a cluster randomized controlled trial. *Trials* 2015;16:372-72. doi: 10.1186/s13063-015-0890-z
8. NHS. Health Survey for England 2015 Physical activiy in children 2016 [Available from: http://healthsurvey.hscic.gov.uk/media/37752/hse2015-child-phy-act.pdf accessed 17/11/20.
9. term TNL. 2019 [Available from: https://www.longtermplan.nhs.uk/publication/nhs-long-term-plan accessed 15/09/20.
10. Milner J, Green R, Dangour AD, et al. Health effects of adopting low greenhouse gas emission diets in the UK. *BMJ Open* 2015;5(4):e007364. doi: 10.1136/bmjopen-2014-007364
11. Kelder SH, Perry CL, Klepp KI, et al. Longitudinal tracking of adolescent smoking, physical activity, and food choice behaviors. *Am J Public Health* 1994;84(7):1121-26. doi: 10.2105/ajph.84.7.1121
12. Craigie AM, Lake AA, Kelly SA, et al. Tracking of obesity-related behaviours from childhood to adulthood: A systematic review. *Maturitas* 2011;70(3):266-84. doi: 10.1016/j.maturitas.2011.08.005 [published Online First: 2011/09/17]
13. Hanson M, Gluckman P. Developmental origins of noncommunicable disease: population and public health implications. *Am J Clin Nutr* 2011;94(6 Suppl):1754s-58s. doi: 10.3945/ajcn.110.001206 [published Online First: 2011/04/29]
14. Gates M. Advancing the adolescent health agenda. *The Lancet* 2016;387(10036):2358-59. doi: 10.1016/S0140-6736(16)30298-7
15. Organisation WH. Global Accelerated Action for the Health of Adolescents (AAHA!): guidance to support country implementation. Geneva, 2017.
16. Kelishadi R, Azizi-Soleiman F. Controlling childhood obesity: A systematic review on strategies and challenges. *J Res Med Sci* 2014;19(10):993-1008.
17. Calvert S, Dempsey RC, Povey R. Delivering in-school interventions to improve dietary behaviours amongst 11- to 16-year-olds: A systematic review. *Obesity Reviews* 2019;20(4):543-53. doi: https://doi.org/10.1111/obr.12797

For peer review only - http://bmjopen.bmj.com/site/about/guidelines.xhtml

18. Woods-Townsend K, Hardy-Johnson P, Bagust L, et al. A cluster-randomised controlled trial of the LifeLab education intervention to improve health literacy in adolescents. *PLoS One* in press
19. Ofcom. Children and parents: Media use and attitudes report 2019 [Available from: https://www.ofcom.org.uk/__data/assets/pdf_file/0023/190616/children-media-use-attitudes-2019-report.pdf accessed 10/09/20.
20. Strömmer S, Barrett M, Woods-Townsend K, et al. Engaging adolescents in changing behaviour (EACH-B): a study protocol for a cluster randomised controlled trial to improve dietary quality and physical activity. *Trials* 2020;21(1):859. doi: 10.1186/s13063-020-04761-w
21. Borde R, Smith JJ, Sutherland R, et al. Methodological considerations and impact of school-based interventions on objectively measured physical activity in adolescents: a systematic review and meta-analysis. *Obesity Reviews* 2017;18(4):476-90. doi: https://doi.org/10.1111/obr.12517
22. Buxton MJ, Drummond MF, Van Hout BA, et al. Modelling in Ecomomic Evaluation: An Unavoidable Fact of Life. *Health Economics* 1997;6(3):217-27. doi: https://doi.org/10.1002/(SICI)1099-1050(199705)6:3<217::AID-HEC267>3.0.CO;2-W
23. NHS Digital. Health Survey for England 2016. 2017.
24. Digital N. Health Survey for England 2015 [Available from: https://digital.nhs.uk/data-and-information/publications/statistical/health-survey-for-england/health-survey-for-england-20152020.
25. Sharma M, Nazareth I, Petersen I. Trends in incidence, prevalence and prescribing in type 2 diabetes mellitus between 2000 and 2013 in primary care: a retrospective cohort study. *BMJ Open* 2016;6(1):e010210. doi: 10.1136/bmjopen-2015-010210 [published Online First: 2016/01/16]
26. McManus S BP, Jenkins R, Brugha T. Mental health and wellbeing in England: Adult Psychiatric Morbidity Survey 2014. 2016.
27. NHS Digital. Mental health of children and young people in England, 2017. 2017.
28. Stegenga BT, Kamphuis MH, King M, et al. The natural course and outcome of major depressive disorder in primary care: the PREDICT-NL study. *Soc Psychiatry Psychiatr Epidemiol* 2012;47(1):87-95. doi: 10.1007/s00127-010-0317-9 [published Online First: 2010/11/09]
29. Singhal A, Ross J, Seminog O, et al. Risk of self-harm and suicide in people with specific psychiatric and physical disorders: comparisons between disorders using English national record linkage. *J R Soc Med* 2014;107(5):194-204. doi: 10.1177/0141076814522033 [published Online First: 2014/02/15]
30. Office of National Statistics. Births in England and Wales: 2017. 2018.
31. Gray N, Picone G, Sloan F, et al. Relation between BMI and diabetes mellitus and its complications among US older adults. *South Med J* 2015;108(1):29-36. doi: 10.14423/SMJ.0000000000000214 [published Online First: 2015/01/13]
32. Sutaria S, Devakumar D, Yasuda SS, et al. Is obesity associated with depression in children? Systematic review and meta-analysis. *Arch Dis Child* 2019;104(1):64-74. doi: 10.1136/archdischild-2017-314608 [published Online First: 2018/07/01]
33. McDonald SD, Han Z, Mulla S, et al. Overweight and obesity in mothers and risk of preterm birth and low birth weight infants: systematic review and meta-analyses. *BMJ* 2010;341:c3428. doi: 10.1136/bmj.c3428 [published Online First: 2010/07/22]
34. Schuch FB, Vancampfort D, Firth J, et al. Physical Activity and Incident Depression: A Meta-Analysis of Prospective Cohort Studies. *Am J Psychiatry* 2018;175(7):631-48. doi: 10.1176/appi.ajp.2018.17111194 [published Online First: 2018/04/25]

For peer review only - http://bmjopen.bmj.com/site/about/guidelines.xhtml

35. Wahid A, Manek N, Nichols M, et al. Quantifying the Association Between Physical Activity and Cardiovascular Disease and Diabetes: A Systematic Review and Meta‐Analysis. *Journal of the American Heart Association* 2016;5(9) doi: 10.1161/jaha.115.002495
36. Chireh B, Li M, D'Arcy C. Diabetes increases the risk of depression: A systematic review, meta-analysis and estimates of population attributable fractions based on prospective studies. *Prev Med Rep* 2019;14:100822. doi: 10.1016/j.pmedr.2019.100822 [published Online First: 2019/03/01]
37. Liu Z, Xu HM, Wen LM, et al. A systematic review and meta-analysis of the overall effects of school-based obesity prevention interventions and effect differences by intervention components. *Int J Behav Nutr Phys Act* 2019;16(1):95. doi: 10.1186/s12966-019-0848-8 [published Online First: 2019/10/31]
38. Lavelle HV, Mackay DF, Pell JP. Systematic review and meta-analysis of school-based interventions to reduce body mass index. *J Public Health (Oxf)* 2012;34(3):360-9. doi: 10.1093/pubmed/fdr116 [published Online First: 2012/01/24]
39. Jacob CM, Hardy-Johnson PL, Inskip HM, et al. A systematic review and meta-analysis of school-based interventions with health education to reduce body mass index in adolescents aged 10 to 19 years. *Int J Behav Nutr Phys Act* 2021;18(1):1. doi: 10.1186/s12966-020-01065-9 [published Online First: 2021/01/06]
40. Mears R, Jago R. Effectiveness of after-school interventions at increasing moderate-to-vigorous physical activity levels in 5- to 18-year olds: a systematic review and meta-analysis. *Br J Sports Med* 2016;50(21):1315-24. doi: 10.1136/bjsports-2015-094976 [published Online First: 2016/05/26]
41. Statistics OfN. National life tables: England. 2020. https://www.ons.gov.uk/peoplepopulationandcommunity/birthsdeathsandmarriages/lifeexpectancies/datasets/nationallifetablesenglandreferencetables (accessed 22/03/2021).
42. Mulnier HES, H.E.;Raleigh,V.S.;Soedamah-Muthu,S.S.;Colhoun,H.M.;Lawrenson,R.A.;. Mortality in people with Type 2 diabetes in the UK. *Diabetic Medicine* 2006(23):516-21.
43. Maheswaran H, Petrou S, Rees K, et al. Estimating EQ-5D utility values for major health behavioural risk factors in England. *J Epidemiol Community Health* 2013;67(2):172-80. doi: 10.1136/jech-2012-201019 [published Online First: 2012/07/31]
44. Janssen MF, Lubetkin EI, Sekhobo JP, et al. The use of the EQ-5D preference-based health status measure in adults with Type 2 diabetes mellitus. *Diabet Med* 2011;28(4):395-413. doi: 10.1111/j.1464-5491.2010.03136.x [published Online First: 2011/03/12]
45. Astrom M, Persson C, Linden-Bostrom M, et al. Population health status based on the EQ-5D-Y-3L among adolescents in Sweden: Results by sociodemographic factors and self-reported comorbidity. *Qual Life Res* 2018;27(11):2859-71. doi: 10.1007/s11136-018-1985-2 [published Online First: 2018/09/10]
46. Lynch FD, JF.; Feeny, DH.; Clarke, GN.; MacMillan, AL.;. Measuring Health-related Quality of Life in Teens With and Without Depression. *Medical Care* 2016;54(12):8.
47. Morriss R, Garland A, Nixon N, et al. Efficacy and cost-effectiveness of a specialist depression service versus usual specialist mental health care to manage persistent depression: a randomised controlled trial. *The Lancet Psychiatry* 2016;3(9):821-31. doi: 10.1016/s2215-0366(16)30143-2
48. Petrou S, Krabuanrat N, Khan K. Preference-Based Health-Related Quality of Life Outcomes Associated with Preterm Birth: A Systematic Review and Meta-analysis.

For peer review only - http://bmjopen.bmj.com/site/about/guidelines.xhtml

Pharmacoeconomics 2019 doi: 10.1007/s40273-019-00865-7 [published Online First: 2019/12/10]
49. Curtis LB, A.;. Unit costs of Health and Social Care 2018. 2018.
50. Hex N, Bartlett C, Wright D, et al. Estimating the current and future costs of Type 1 and Type 2 diabetes in the UK, including direct health costs and indirect societal and productivity costs. *Diabet Med* 2012;29(7):855-62. doi: 10.1111/j.1464-5491.2012.03698.x [published Online First: 2012/04/28]
51. Kuyken W, Hayes R, Barrett B, et al. Effectiveness and cost-effectiveness of mindfulness-based cognitive therapy compared with maintenance antidepressant treatment in the prevention of depressive relapse or recurrence (PREVENT): a randomised controlled trial. *The Lancet* 2015;386(9988):63-73. doi: 10.1016/s0140-6736(14)62222-4
52. Khan KA, Petrou S, Dritsaki M, et al. Economic costs associated with moderate and late preterm birth: a prospective population-based study. *BJOG* 2015;122(11):1495-505. doi: 10.1111/1471-0528.13515 [published Online First: 2015/07/30]
53. Mangham LJ, Petrou S, Doyle LW, et al. The cost of preterm birth throughout childhood in England and Wales. *Pediatrics* 2009;123(2):e312-27. doi: 10.1542/peds.2008-1827 [published Online First: 2009/01/28]
54. Hamilton D, Dee A, Perry IJ. The lifetime costs of overweight and obesity in childhood and adolescence: a systematic review. *Obes Rev* 2018;19(4):452-63. doi: 10.1111/obr.12649 [published Online First: 2017/12/23]
55. The National Institute for Health and Care Excellence. Guide to the methods of technology appraisal, 2013.
56. Briggs AH, Claxton K, Sculpher MJ. Decision Modelling for Health Economic Evaluation - Handbooks in Health Economic Evaluation Series. Oxford University Press. 2006.
57. Anderson YC, Leung W, Grant CC, et al. Economic evaluation of a multi-disciplinary community-based intervention programme for New Zealand children and adolescents with obesity. *Obesity Research & Clinical Practice* 2018;12(3):293-98. doi: https://dx.doi.org/10.1016/j.orcp.2018.04.001
58. Ladapo JA, Bogart LM, Klein DJ, et al. Cost and Cost-Effectiveness of Students for Nutrition and eXercise (SNaX). *Academic pediatrics* 2016;16(3):247-53. doi: https://dx.doi.org/10.1016/j.acap.2015.07.009
59. Lee BY, Adam A, Zenkov E, et al. Modeling The Economic And Health Impact Of Increasing Children's Physical Activity In The United States. *Health Affairs* 2017;36(5):902-08. doi: https://dx.doi.org/10.1377/hlthaff.2016.1315
60. Panca M, Christie D, Cole TJ, et al. Cost-effectiveness of a community-delivered multicomponent intervention compared with enhanced standard care of obese adolescents: cost-utility analysis alongside a randomised controlled trial (the HELP trial). *BMJ Open* 2018;8(2):e018640. doi: https://dx.doi.org/10.1136/bmjopen-2017-018640
61. Sutherland R, Reeves P, Campbell E, et al. Cost-effectiveness of a multi-component school-based physical activity intervention targeting adolescents: the 'Physical Activity 4 Everyone' cluster randomized trial. *International Journal of Behavioral Nutrition & Physical Activity* 2016;13:94. doi: https://dx.doi.org/10.1186/s12966-016-0418-2

For peer review only - http://bmjopen.bmj.com/site/about/guidelines.xhtml

For peer review only

For peer review only - http://bmjopen.bmj.com/site/about/guidelines.xhtml

Figure 1: Illustration of the conceptual framework and model structure

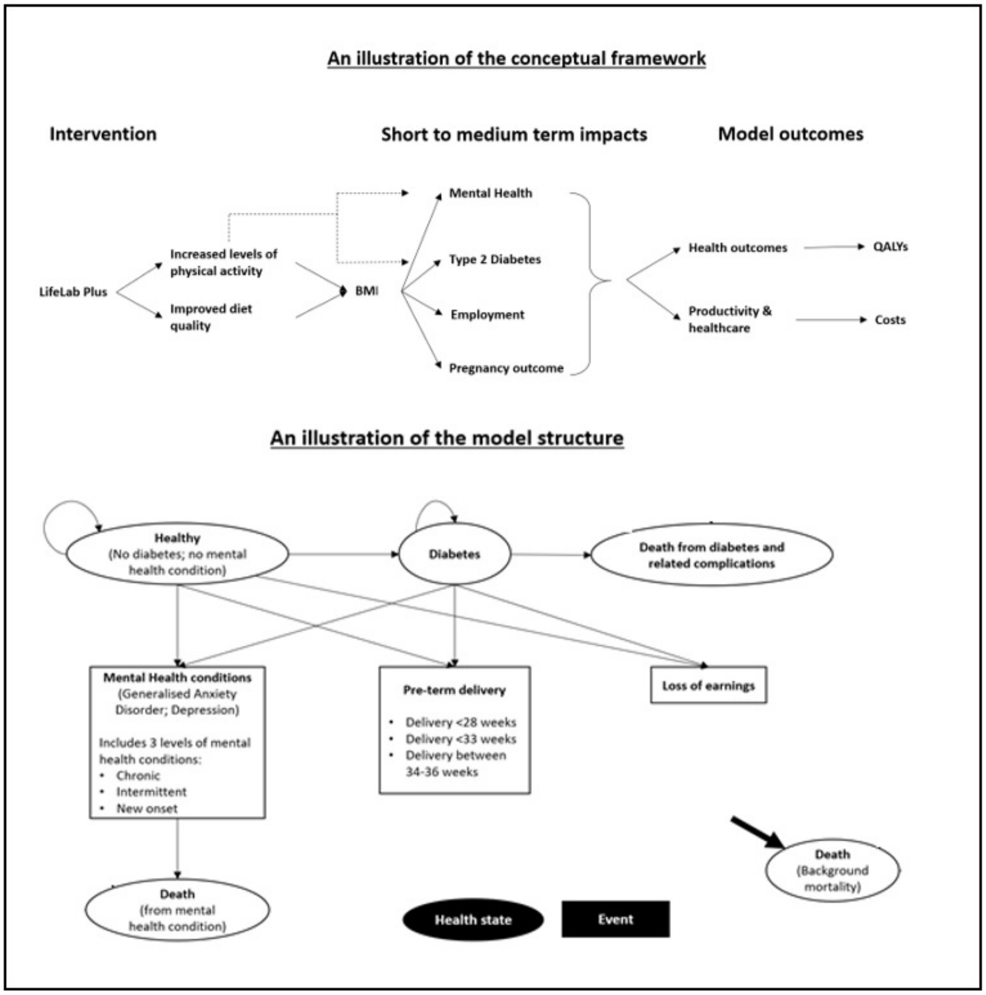

Figure 1: Illustration of the conceptual framework and model structure

137x145mm (144 x 144 DPI)

Figure 2: Tornado diagram to show the impact of varying parameter input values on ICERs

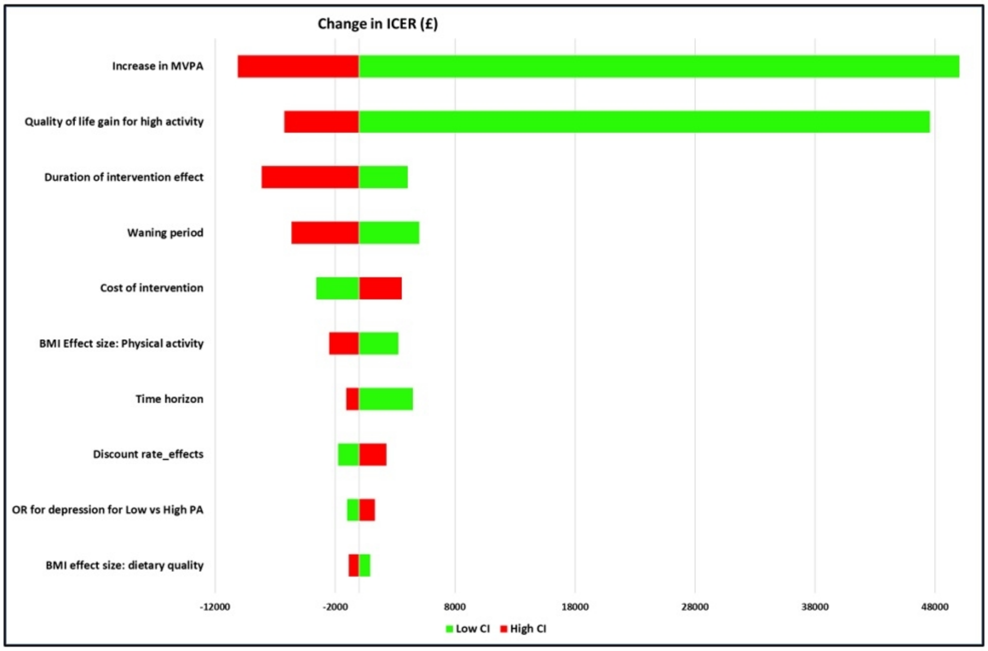

MVPA: Moderate or Vigorous Physical Activity; BMI: Body Mass Index; CI: Confidence Interval; ICER: Incremental Cost Effectiveness Ratio

Figure 2: Tornado diagram to show the impact of varying parameter input values on ICERs

175x127mm (144 x 144 DPI)

Figure 3: Scatter plot of PSA cost-effectiveness results

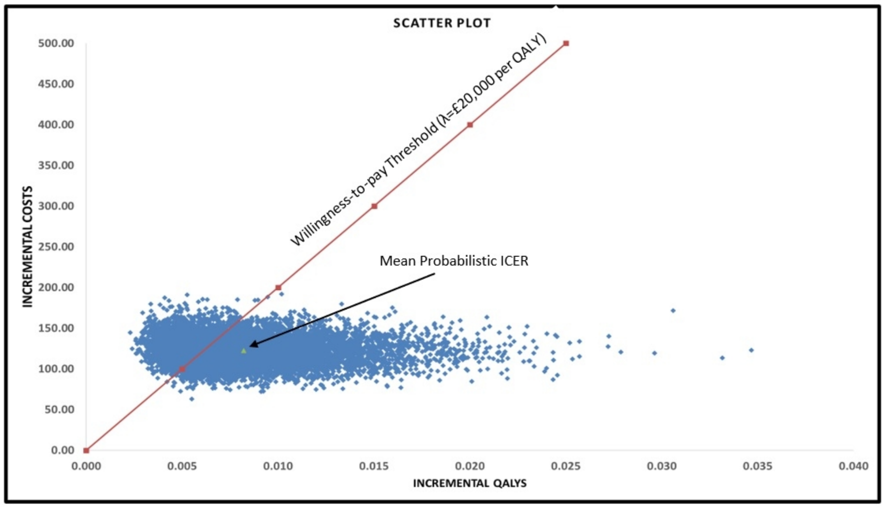

The probabilistic model was run for 10,000 simulations. All the plots fall in the North-East quadrant of the Cost-effectiveness plane, indicating the intevention- LifeLab Plus is associated with higher costs and higher QALYs compared to usual schooling. Majority of the plots, including the mean ICER, fall within the willingness-to-pay threshold of £20,000 per QALY.

Figure 3: Scatter plot of PSA cost-effectiveness results

199x122mm (144 x 144 DPI)

1 APPENDIX A

Model parameters were estimated by using the following equations. For the control arm, rate was based upon age only. For the intervention arm, rate was based on age and the change in BMI from the control population to the intervention.

Table 1: Parameter equations

Parameter	Equation	Units	Source
BMI - men	-0.0031*age^2+0.4096*age+16.098	Kg/m2	HSE 2017[1]
BMI - women	-0.0032*Age^2 + 0.4235*Age+15.812	Kg/m2	
Type 2 Diabetes Men Control, age <25y	0.025*Age-0.265	Incidence per 1000	Sharma 2015[2]
Type 2 Diabetes Men Control, age >25y	0.0054Age^2-0.191Age+1.7142	Incidence per 1000	
Type 2 Diabetes Women, Control, all ages	0.0031Age^2-0.0942Age+1.2045	Incidence per 1000	
Type 2 Diabetes Men, Intervention, age < 25y	(0.025*Age-0.265)/(0.081BMI_control-0.8451)*(0.081BMI_int-0.8451)	Incidence per 1000	Gray et al 2015[3]
Type 2 Diabetes Men, Intervention, > 25y	(0.0054Age^2-0.191Age+1.7142)/(0.081BMI_control-0.8451)*(0.081BMI_int-0.8451)	Incidence per 1000	
Type 2 Diabetes Women Intervention, all ages	(0.0031Age^2-0.0942Age+1.2045)/(0.0637BMI_control -0.3135)*(0.0637BMI_Int-0.3135)	Incidence per 1000	
Mental health Men, control	-0.00008*Age^2+0.0073*Age-0.0544	% Incidence	APMS 2014[4]
Mental health Women, control	-0.00007*Age^2+0.0062*Age+0.0034	% Incidence	
Mental Health Men, intervention	(-0.00007*Age^2+0.0062*Age+0.0034)*(0.0127*BMI_I+0.727)/(0.0127*BMI_C +0.727)	% Incidence	Sutaria et al 2018[5]
Mental Health Women, intervention	(-0.00007*Age^2+0.0062*Age+0.0034) *(0.0391*BMI_I+0.1087)/(0.0391*BMI_C+0.1087)	% Incidence	
Mental Health, diabetes	Multiply MH risk by 1.33		Chireh et al 2019[6]
Mental Health, Physical exercise	OR 0.83 high activity vs low activity. Applied to intervention arm only.		Schuch et al 2018[7]
Preterm birth <33 weeks, control	-0.0512*Age^2+3.0922*Age-37.891	Incidence per 1000	ONS 2017[8]
Preterm birth <33 weeks, intervention	(-0.0512*Age2+3.0922*Age-37.891)*(0.0514*BMI_I-0.1712)/(0.0514*BMI_C-0.1712)	Incidence per 1000	Mcdonald et al 2010[9]
Loss of earnings, men	12534BMI – 297703	£ over lifetime	

For peer review only

For peer review only - http://bmjopen.bmj.com/site/about/guidelines.xhtml

Parameter	Equation	Units	Source
Loss of earning, women	17695BMI - 439796	£ over lifetime	Hamilton et al 2018[10]

Table 2: Parameter equations for z-scores

Parameter	Equation	Units	Source
Mental Health Men, Intervention	(0.0054Age^2-0.191Age+1.7142)/(0.0274BMI_z_score_control+1.0011)*(0.0274BMI_z_scores_int+1.011)	Incidence per 1000	Gray et al 2015[3]
Mental Health Women, Intervention	(0.0031Age^2-0.0942Age+1.2045)/(0.084BMI_z_scores_control+ 0.9486)*(0.084BMI_z_scores_Int+0.9486)	Incidence per 1000	
Type 2 Diabetes Men, intervention	(-0.00007*Age^2+0.0062*Age+0.0034)/(0.1742BMI_z_scores_control+ 0.8964)*(0.1742BMI_z_scores_Int+0.8964)	% Incidence	Sutaria et al 2018[5]
Type 2 Diabetes Women, intervention	(-0.00007*Age^2+0.0062*Age+0.0034) *(0.1369*BMI_z_scores_I+1.0556)/(0.1369*BMI_z_scores_C+1.0556)	% Incidence	
Preterm birth <33 weeks, intervention	(-0.0512*Age2+3.0922*Age-37.891)*(0.1105*BMI_z_scores_I+0.9336)/(0.1105*BMI_z_scores_C+0.9336)	Incidence per 1000	Mcdonald et al 2010[9]

Table 3: Parameter equation for quality of life estimates

Parameter	Equation	Source
Quality of life, by age	0.9508566+0.0212126*Male-0.0002587*Age-0.0000332*Age^2	Maheswaran et al 2012[11]
Disutility for BMI, if BMI < 30	-0.0009BMI + 0.0189	
Disutility for BMI, if BMI > 30	-0.0036BMI+0.094	

For peer review only - http://bmjopen.bmj.com/site/about/guidelines.xhtml

Table 4: Input parameters used in the model

Parameter	Value	SD/SE	95% CI-Low	95% CI-High	Distribution
**Direct trial efficacy**					
BMI effect size: Physical activity	0.13	0.046	0.040	0.220	Beta
BMI effect size: Dietary quality	0.04	0.01423	0.012	0.068	Beta
BMI z-scores effect size: Physical activity	0.04	0.02	0.001	0.079	Beta
BMI z-scores effect size: Dietary quality	0.02	0.01	0.000	0.040	Beta
Prop. meeting PA guidelines control	0.13	0.0013	0.127	0.133	Beta
Prop. meeting PA guidelines intervention	0.16	0.049	0.117	0.257	Beta
Increase in Moderate to Vigorous PA	4.84	2.944	-0.940	10.610	Beta
**Relative effectiveness**					
OR for depression for Low vs High Physical Activity	0.83	0.026	0.790	0.880	Beta
OR of diabetes increasing the risk of depression	1.33	0.133	1.069	1.591	Lognormal
RR of PA on incidence of diabetes	0.74	0.074	0.595	0.885	Lognormal
**Mortality**					
Mental health: risk of death by suicide	0.001	0.000	0.001	0.001	Beta
HR of mortality those with type 2 Diabetes aged <65 years	2.978	0.190	2.605	3.350	Lognormal
HR of mortality those with type 2 Diabetes aged >65 years	1.958	0.050	1.860	2.056	Lognormal
**Relative effectiveness[a]**					
Pre-term birth relative risk for BMI P1	0.051	0.005	0.041	0.061	Beta
Pre-term birth relative risk for BMI P2	-0.171		0.111	-0.322	
MH BMI relative risk Boys P1	0.013	0.004	0.004	0.021	Beta
MH BMI relative risk Boys P2	0.727		0.914	0.540	
MH BMI relative risk girls P1	0.039	0.004	0.031	0.047	Beta
MH BMI relative risk girls P2	0.109		0.324	-0.005	
Diabetes relative risk boys P1	0.081	0.004	0.073	0.089	Beta
Diabetes relative risk boys P2	-0.845		-0.571	-0.912	
Diabetes relative risk girls P1	0.064	0.013	0.039	0.089	Beta

For peer review only - http://bmjopen.bmj.com/site/about/guidelines.xhtml

Parameter	Value	SD/SE	95% CI-Low	95% CI-High	Distribution
Diabetes relative risk girls P2	-0.314		0.167	-0.906	
Loss of earnings Men P1	12036	601.8	10856	13215.5	Gamma
Loss of earnings Men P2	-284478		-256213	-311886.5	
Loss of earnings Women P1	16689	1668.9	13418	19960.0	Gamma
Loss of earnings Women P2	-412424		-339474	-504989.1	
**Z scores [a]**					
Pre-term birth relative risk for BMI z-scores P1	0.1105	0.011	0.089	0.132	Beta
Pre-term birth relative risk for BMI z-scores P2	0.9336		-0.910	-1.841	
MH BMI z-scores relative risk Boys P1	0.0274	0.003	0.022	0.033	Beta
MH BMI z-scores relative risk Boys P2	1.0011		0.526	0.295	
MH BMI z-scores relative risk girls P1	0.084	0.008	0.068	0.100	Beta
MH BMI z-scores relative risk girls P2	0.9486		-0.452	-1.160	
Diabetes relative risk boys P1	0.1742	0.009	0.157	0.191	Beta
Diabetes relative risk boys P2	0.8964		-2.378	-3.112	
Diabetes relative risk girls P1	0.1369	0.027	0.083	0.191	Beta
Diabetes relative risk girls P2	1.0556		-0.790	-3.097	
**Proportions of population with mental health conditions**					
Chronic	0.170	0.017	0.137	0.203	Dirichlet
intermittent	0.400	0.040	0.322	0.478	Dirichlet
New and one-off	0.430	0.043	0.346	0.514	Dirichlet

[a] Risk equation is in the form: Relative risk = P1*BMI + P2

For peer review only - http://bmjopen.bmj.com/site/about/guidelines.xhtml

2 APPENDIX B

A breakdown of the resources used for estimating the costs of the intervention i.e. LifeLab Plus is presented below. The intervention comprises of three components:

i. Life Lab
ii. Digital App
iii. Teachers' Support (Healthy Conversation skills)

The costs are estimated for one-year that is, for the trial duration. The assumptions related to resources-used are based on a discussion with KTW on 5th June 2019.

Table 5: Life Lab

Item	Number	Comment
Number of classes per year	50	
No of pupils in each class	25	Varies between 25-32; assumed 25 for the base case
Total pupils	1250	=50 classes x 25 pupils per class
No of schoolteachers in each class	1	
Total teachers	50	

Table 6: Staff Training for Life Lab and Healthy Conversation Skills

Item	Number	Comment
Lifelab staff / training day (days)	6	Each training day / Lifelab day consists of 6 staff days to run. 4 staff days to run training and preparation and 2 days of administration.
Teacher training		
No. of Training days	5	Each training day is for 10 teachers.
Lifelab Staff days	30	=5 training days x 6 staff days / training day
Lifelab		
Number of days	50	=50x1
Lifelab Staff days	300	=50 life lab days x 6 staff day / lifelab
Scientists		Each lifelab day consists of 3 scientists
Number trained	75	Assumed each scientist does two lifelab days
Training days	3	=75 scientists /25; 25 trained per day
Lifelab Staff days	18	=3 training days x 6 staff days / training day
Training the trainers (Healthy Conversation Skills)		
Trainers	6	Assumption
Lifelab Staff days	1	Assume takes 2 hours
Showcase		All schools
Lifelab Staff days	3.5	1.5 days prep; 1 day delivery for 2 staff
Total lifelab staff days	352.5	Band 4 teacher

For peer review only - http://bmjopen.bmj.com/site/about/guidelines.xhtml

Data collection – Lifelab
Each class has two hours of data collection by two Lifelab staffs, 2 research nurses and 1 research assistant.

Table 7: Costs for data collection

Item	Number	Comment
Lifelab staff days	28.6	=2 hours x2 staff x50 (lifelab days)/7 (hours)
Research nurse	28.6	=2 hours x2 staff x50 (lifelab days)/7 (hours)
Research assistant	14.3	=2 hours x1 staff x50 (lifelab days)/7 (hours)

Table 8: Staff unit costs

Staff costs	Salary	Comment
Band 4 teacher	£34,189	i.e. £163 per day, Assumes 210 working days per year
Research assistant	£27,025	i.e. £129 per day, Assumes 210 working days per year
Research nurse Band 5	£26,231	i.e £125 per day, Assumes 210 working days per year

Table 9: Staff total costs

Staff costs	Total	Comment
Lifelab / training	£71,736	=£34,189/210x352.5*1.25;<br>= salary / working days per year*lifelab staff days*NI and pension adjustment<br>Assumes 210 working days per year, pension / NI contributions of 25%
Follow-up data collection	£12,641	=(£34,189*28.6 + £27,025 *28.6 + 26,231 *14.3)/210*1.25<br>= (salary teacher* data collection days teacher + salary research nurse* data collection days research nurse + salary research assistant*data collection days research assistant)/working days per year*NI / pension adjustment
Total	£84,377	

Table 10: Digital App development

Item	Total	Comment
Development of app	£324,000	Estimate from expert
Duration of development, year	5	As above
Duration of app, years	10	Assumption
Number of centres using app	10	Assumption
Cost per year development	£64,800	£324,000 / 5; total cost / number of years of development
Maintenance cost	£16,200	Assumed to be 25% of development cost
Cost per year of development across app lifetime per centre	£3240	£324,000 / 100; total development cost / (number of years app lasts for x number of centres)
EACH-B development for new centre	£87,445	Assumed to be 1 year of staff costs.

For peer review only - http://bmjopen.bmj.com/site/about/guidelines.xhtml

Item	Total	Comment
Number of years centre lasts	10	Assumption
Cost per year of centre	£8745	£87,445 / 10

Table 11: Summary of included costs

Item	Total	Comment
Staff costs	£84,377	
Room hire	£26,857	=400 (room hire cost)*67.14 (number of days); assumes cost of hire is £400 / day
Consumables	£15,839	Consumables include costs associated with printing, paper, classroom
Overheads	£37,970	Direct overheads based on 29% of direct care salary costs and indirect overheads based on 16% direct care salary costs (As used in Curtis et al 2019 for overheads for healthcare professionals)
Maintenance of app	£16,200	See above table
Capital cost, development of app	£3240	See above
Capital cost, development of EACH-B	£8745	See above

Table 12: Total costs

Item	Total	Comment
Total cost	£193,228	Sum of all of the above costs
Cost per pupil	£154.58	=£193,228/1250; (total cost /total number of pupils)

For peer review only

3 APPENDIX C

Figure 1: Cost-Effectiveness Acceptability Curve (CEAC)

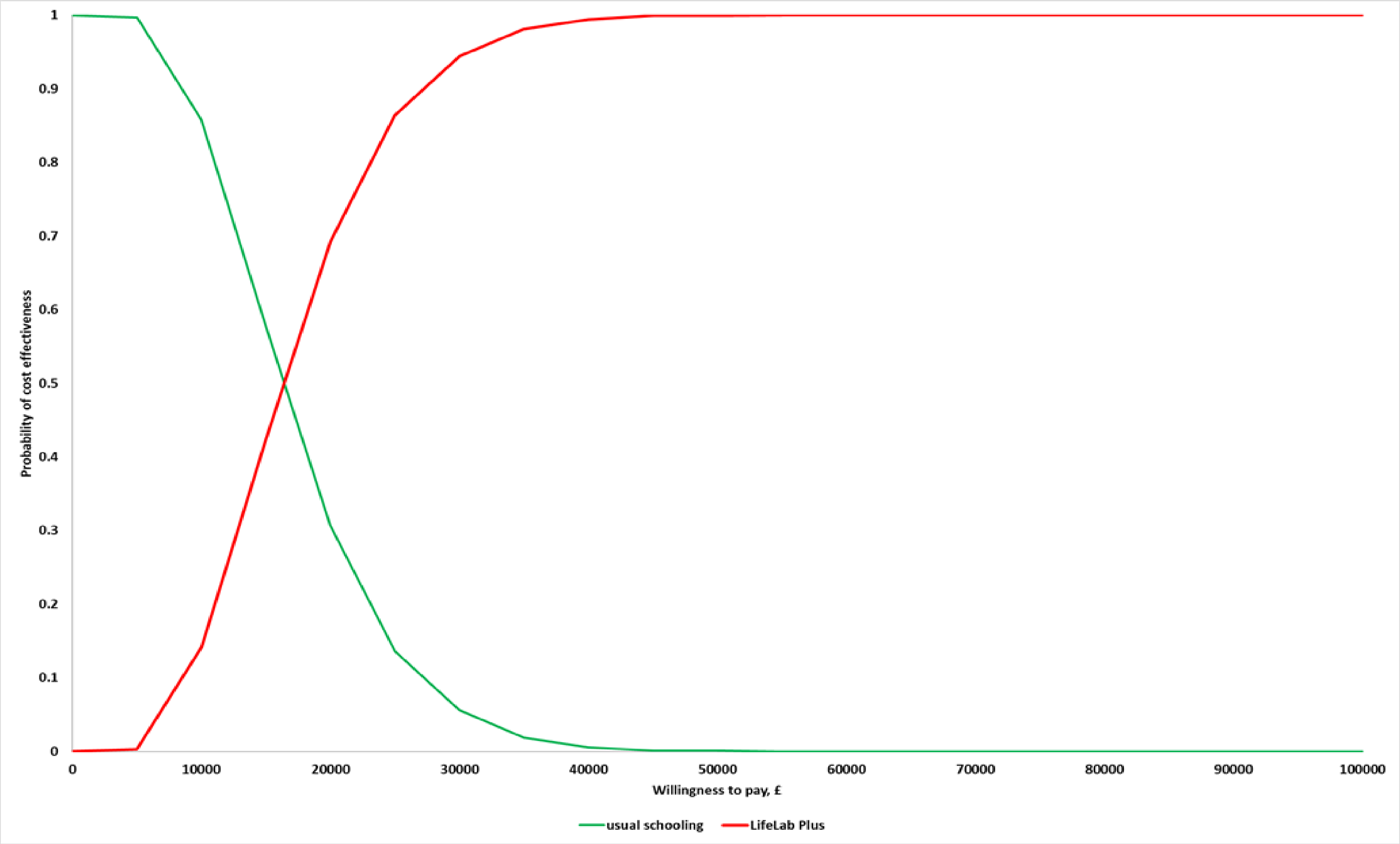

For peer review only - http://bmjopen.bmj.com/site/about/guidelines.xhtml

REFERENCES

1. NHS Digital. Health Survey for England 2016. 2017.
2. Sharma M, Nazareth I, Petersen I. Trends in incidence, prevalence and prescribing in type 2 diabetes mellitus between 2000 and 2013 in primary care: a retrospective cohort study. *BMJ Open* 2016;6(1):e010210. doi: 10.1136/bmjopen-2015-010210 [published Online First: 2016/01/16]
3. Gray N, Picone G, Sloan F, et al. Relation between BMI and diabetes mellitus and its complications among US older adults. *South Med J* 2015;108(1):29-36. doi: 10.14423/SMJ.0000000000000214 [published Online First: 2015/01/13]
4. McManus S BP, Jenkins R, Brugha T. Mental health and wellbeing in England: Adult Psychiatric Morbidity Survey 2014. 2016.
5. Sutaria S, Devakumar D, Yasuda SS, et al. Is obesity associated with depression in children? Systematic review and meta-analysis. *Arch Dis Child* 2019;104(1):64-74. doi: 10.1136/archdischild-2017-314608 [published Online First: 2018/07/01]
6. Chireh B, Li M, D'Arcy C. Diabetes increases the risk of depression: A systematic review, meta-analysis and estimates of population attributable fractions based on prospective studies. *Prev Med Rep* 2019;14:100822. doi: 10.1016/j.pmedr.2019.100822 [published Online First: 2019/03/01]
7. Schuch FB, Vancampfort D, Richards J, et al. Exercise as a treatment for depression: A meta-analysis adjusting for publication bias. *J Psychiatr Res* 2016;77:42-51. doi: 10.1016/j.jpsychires.2016.02.023 [published Online First: 2016/03/16]
8. Office of National Statistics. Births in England and Wales: 2017. 2018.
9. McDonald SD, Han Z, Mulla S, et al. Overweight and obesity in mothers and risk of preterm birth and low birth weight infants: systematic review and meta-analyses. *BMJ* 2010;341:c3428. doi: 10.1136/bmj.c3428 [published Online First: 2010/07/22]
10. Hamilton D, Dee A, Perry IJ. The lifetime costs of overweight and obesity in childhood and adolescence: a systematic review. *Obes Rev* 2018;19(4):452-63. doi: 10.1111/obr.12649 [published Online First: 2017/12/23]
11. Maheswaran H, Petrou S, Rees K, et al. Estimating EQ-5D utility values for major health behavioural risk factors in England. *J Epidemiol Community Health* 2013;67(2):172-80. doi: 10.1136/jech-2012-201019 [published Online First: 2012/07/31]

For peer review only

For peer review only - http://bmjopen.bmj.com/site/about/guidelines.xhtml

CHEERS checklist—Items to include when reporting economic evaluations of health interventions

Section/item	Item No	Recommendation	Reported on page No/ line No
**Title and abstract**			
Title	1	Identify the study as an economic evaluation or use more specific terms such as "cost-effectiveness analysis", and describe the interventions compared.	page 1, line 1
Abstract	2	Provide a structured summary of objectives, perspective, setting, methods (including study design and inputs), results (including base case and uncertainty analyses), and conclusions.	page 2, lines 29 to 54
**Introduction**			
Background and objectives	3	Provide an explicit statement of the broader context for the study.	page 4, line 110 to page 5, line 137
		Present the study question and its relevance for health policy or practice decisions.	page 5, line 139 to 149
**Methods**			
Target population and subgroups	4	Describe characteristics of the base case population and subgroups analysed, including why they were chosen.	page 6, line 172 to 175
Setting and location	5	State relevant aspects of the system(s) in which the decision(s) need(s) to be made.	page 4, line 125 to page 5, line 137 and line 145 to line 149;
Study perspective	6	Describe the perspective of the study and relate this to the costs being evaluated.	page 11, line 348 to 350; page 14, line 438;
Comparators	7	Describe the interventions or strategies being compared and state why they were chosen.	page 5, line 139 to 149; page 6, line 174 to 175
Time horizon	8	State the time horizon(s) over which costs and consequences are being evaluated and say why appropriate.	page 7, line 214 to line 217 and line 222
Discount rate	9	Report the choice of discount rate(s) used for costs and outcomes and say why appropriate.	page 11, line 350
Choice of health outcomes	10	Describe what outcomes were used as the measure(s) of benefit in the evaluation and their relevance for the type of analysis performed.	page 7, line 201 to line 207
Measurement of effectiveness	11a	*Single study-based estimates:* Describe fully the design features of the single effectiveness study and why the single study was a sufficient source of clinical effectiveness data.	not applicable
	11b	*Synthesis-based estimates*: Describe fully the methods used for identification of included studies and synthesis of clinical effectiveness data.	page 8, line 229 to page 10, line 306; supplement appendix A
Measurement and valuation of preference based outcomes	12	If applicable, describe the population and methods used to elicit preferences for outcomes.	not applicable
Estimating resources and costs	13a	*Single study-based economic evaluation:*Describe approaches used to estimate resource use associated with the alternative interventions. Describe primary or secondary research methods for valuing each resource item in terms of its unit cost. Describe any adjustments made to approximate to opportunity costs.	not applicable

For peer review only

Section/item	Item No	Recommendation	Reported on page No/ line No
	13b	*Model-based economic evaluation:* Describe approaches and data sources used to estimate resource use associated with model health states. Describe primary or secondary research methods for valuing each resource item in terms of its unit cost. Describe any adjustments made to approximate to opportunity costs.	page 10, line 309 to page 11, line 339; pages 17-18, table 1; supplement Appendix B
Currency, price date, and conversion	14	Report the dates of the estimated resource quantities and unit costs. Describe methods for adjusting estimated unit costs to the year of reported costs if necessary. Describe methods for converting costs into a common currency base and the exchange rate.	page 11, line 324 to 325; page 11, line 337 to 339;
Choice of model	15	Describe and give reasons for the specific type of decision-analytical model used. Providing a figure to show model structure is strongly recommended.	page 5, line 153 to page 6, line 162; figure 1.
Assumptions	16	Describe all structural or other assumptions underpinning the decision-analytical model.	Page 6, line 159 to 160; Page 6, line 181 to 182; Page 7, line 210 to 211 and line 216 to 220; Page 8, line 242 to 244, 247 to 250, 253 to 254; Page 9, line 261 to 262, line 267 to 273, line 281 to 283, 286 to 288 page 10 line 306; Page 10, line 312 to 319; Page 11, line 327 to line 334; Page 12, line 363 to line 367; Page 13, line 406 to 407; Page 14, line 431 to 433, line 441 to 442.
Analytical methods	17	Describe all analytical methods supporting the evaluation. This could include methods for dealing with skewed, missing, or censored data; extrapolation methods; methods for pooling data; approaches to validate or make adjustments (such as half cycle corrections) to a model; and methods for handling population heterogeneity and uncertainty.	Page 11, line 342 to 345; page 12, line 358 to line 369; Supplement Appendix A, Table 4
**Results**			
Study parameters	18	Report the values, ranges, references, and, if used, probability distributions for all parameters. Report reasons or sources for distributions used to represent uncertainty where appropriate. Providing a table to show the input values is strongly recommended.	Page 7, line 209 to page 12, line 369; Pages 17 to 18, table 1; supplement Appendix A, Table 1 to 4.
Incremental costs and outcomes	19	For each intervention, report mean values for the main categories of estimated costs and outcomes of interest, as well as mean differences between the comparator groups. If applicable, report incremental cost-effectiveness ratios.	page 12, line 381 to 385; pages 19 to 20, table 2
Characterising uncertainty	20a	*Single study-based economic evaluation:*Describe the effects of sampling uncertainty for the estimated incremental cost and incremental effectiveness	not applicable

For peer review only

Section/item	Item No	Recommendation	Reported on page No/ line No
		parameters, together with the impact of methodological assumptions (such as discount rate, study perspective).	
	20b	*Model-based economic evaluation:* Describe the effects on the results of uncertainty for all input parameters, and uncertainty related to the structure of the model and assumptions.	page 12, line 388 to page 13, line 399; figures 2 and 3; pages 19 to 20, table 2; supplement Appendix C figure 1
Characterising heterogeneity	21	If applicable, report differences in costs, outcomes, or cost-effectiveness that can be explained by variations between subgroups of patients with different baseline characteristics or other observed variability in effects that are not reducible by more information.	not applicable
**Discussion**			
Study findings, limitations, generalisability, and current knowledge	22	Summarise key study findings and describe how they support the conclusions reached. Discuss limitations and the generalisability of the findings and how the findings fit with current knowledge.	page 13, line 403 to page 15, line 476
**Other**			
Source of funding	23	Describe how the study was funded and the role of the funder in the identification, design, conduct, and reporting of the analysis. Describe other non-monetary sources of support.	page 16, line 499 to line 505
Conflicts of interest	24	Describe any potential for conflict of interest of study contributors in accordance with journal policy. In the absence of a journal policy, we recommend authors comply with International Committee of Medical Journal Editors recommendations.	page 15, line 507 to line 510

For consistency, the CHEERS statement checklist format is based on the format of the CONSORT statement checklist

For peer review only

BMJ Open

Cost-effectiveness of a dietary and physical activity intervention in adolescents: a prototype modelling study based on the Engaging Adolescents in Changing Behaviour (EACH-B) programme

Journal:	*BMJ Open*
Manuscript ID	bmjopen-2021-052611.R3
Article Type:	Original research
Date Submitted by the Author:	14-Jun-2022
Complete List of Authors:	Kalita, Neelam; University of Southampton, Southampton Health Technology Assessments Centre<br>Cooper, Keith; Univ Southampton, SHTAC<br>Baird, Janis; University of Southampton, MRC Epidemiology Resource Centre; University of Southampton, NIHR Southampton Biomedical Research Centre<br>Woods-Townsend, Kathryn ; University of Southampton, Southampton Education School, Faculty of Social Sciences; University of Southampton , NIHR Southampton Biomedical Research Centre<br>Godfrey, Keith; University of Southampton and Southampton University Hospitals NHS Trust, MRC Lifecourse Epidemiology Unit; NIHR Southampton Biomedical Research Centre<br>Cooper, Cyrus; University of Southampton and Southampton University Hospitals NHS Trust, MRC Lifecourse Epidemiology Unit; University of Oxford, Nuffield Department of Orthopaedics, Rheumatology and Musculoskeletal Sciences<br>Inskip, Hazel; Medical Research Council, Epidemiology Resource Centre; NIHR Southampton Biomedical Research Centre<br>Barker, Mary; University of Southampton, MRC Lifecourse Epidemiology Unit; NIHR Southampton Biomedical Research Centre<br>Lord, Joanne; University of Southampton, Southampton Health Technology Assessments Centre
**Primary Subject Heading**:	Health economics
Secondary Subject Heading:	Diabetes and endocrinology, Health economics, Mental health, Nutrition and metabolism, Paediatrics
Keywords:	HEALTH ECONOMICS, MENTAL HEALTH, NUTRITION & DIETETICS, General diabetes < DIABETES & ENDOCRINOLOGY, Public health < INFECTIOUS DISEASES

SCHOLARONE™
Manuscripts

I, the Submitting Author has the right to grant and does grant on behalf of all authors of the Work (as defined in the below author licence), an exclusive licence and/or a non-exclusive licence for contributions from authors who are: i) UK Crown employees; ii) where BMJ has agreed a CC-BY licence shall apply, and/or iii) in accordance with the terms applicable for US Federal Government officers or employees acting as part of their official duties; on a worldwide, perpetual, irrevocable, royalty-free basis to BMJ Publishing Group Ltd ("BMJ") its licensees and where the relevant Journal is co-owned by BMJ to the co-owners of the Journal, to publish the Work in this journal and any other BMJ products and to exploit all rights, as set out in our licence.

The Submitting Author accepts and understands that any supply made under these terms is made by BMJ to the Submitting Author unless you are acting as an employee on behalf of your employer or a postgraduate student of an affiliated institution which is paying any applicable article publishing charge ("APC") for Open Access articles. Where the Submitting Author wishes to make the Work available on an Open Access basis (and intends to pay the relevant APC), the terms of reuse of such Open Access shall be governed by a Creative Commons licence – details of these licences and which Creative Commons licence will apply to this Work are set out in our licence referred to above.

Other than as permitted in any relevant BMJ Author's Self Archiving Policies, I confirm this Work has not been accepted for publication elsewhere, is not being considered for publication elsewhere and does not duplicate material already published. I confirm all authors consent to publication of this Work and authorise the granting of this licence.

For peer review only

Cost-effectiveness of a dietary and physical activity intervention in adolescents: a prototype modelling study based on the Engaging Adolescents in Changing Behaviour (EACH-B) programme

Authors: Neelam Kalita[1], Keith Cooper[1], Janis Baird[2;3], Kathryn Woods-Townsend[3;4], Keith M Godfrey[2;3], Cyrus Cooper[2;6;7], Hazel Inskip[2;3], Mary Barker[2;3;5], Joanne Lord[1] and the EACH-B study group

Affiliations:

[1] Southampton Health Technology Assessments Centre, University of Southampton, UK

[2] MRC Lifecourse Epidemiology Unit, University of Southampton, Southampton, UK

[3] NIHR Southampton Biomedical Research Centre, University of Southampton and University Hospital Southampton NHS Foundation Trust, Southampton, UK

[4] Southampton Education School, Faculty of Social Sciences, University of Southampton, Southampton, UK

[5] School of Health Sciences, University of Southampton, Southampton, UK

[6] Faculty of Medicine, University of Southampton

[7] Nuffield Department of Orthopaedics, Rheumatology and Musculoskeletal Sciences, University of Oxford

Corresponding author: Neelam Kalita

Email: N.Kalita@soton.ac.uk

For peer review only

ABSTRACT

Objective To assess costs, health outcomes and cost-effectiveness of interventions that aim to improve quality of diet and level of physical activity in adolescents.

Design A Markov model was developed to assess four potential benefits of healthy behaviour for adolescents: better mental health (episodes of depression and generalised anxiety disorder), higher earnings and reduced incidence of Type 2 diabetes and adverse pregnancy outcomes (in terms of pre-term delivery). The model parameters were informed by published literature. The analysis took a societal perspective over a 20-year period. One-way and probabilistic sensitivity analyses for 10,000 simulations were conducted.

Participants A hypothetical cohort of 100 adolescents with a mean age of 13 years.

Interventions An exemplar school-based, multi-component intervention that was developed by the Engaging Adolescents for Changing Behaviour programme, compared with usual schooling.

Outcome measure Incremental cost-effectiveness ratio as measured by cost per quality-adjusted life year (QALY) gained.

Results The exemplar dietary and physical activity intervention was associated with an incremental cost of £123 per adolescent and better health outcomes with a mean QALY gain of 0.0085 compared with usual schooling, resulting in an ICER of £14,367 per QALY. The key model drivers are the intervention effect on levels of physical activity, quality-of-life gain for high levels of physical activity, the duration of the intervention effects and the period over which effects wane.

Conclusions The results suggested that such an intervention has the potential to offer a cost-effective use of healthcare-resources for adolescents in the UK at a willingness-to-pay threshold of £20,000 per QALY. The model focused on short to medium-term benefits of healthy eating and physical activity exploiting the strong evidence base that exists for this age group. Other benefits in later life, such as reduced cardiovascular risk, are more sensitive to assumptions about the persistence of behavioural change and discounting.

For peer review only

For peer review only - http://bmjopen.bmj.com/site/about/guidelines.xhtml

ARTICLE SUMMARY

Strengths and limitations of this study

- The model was developed, based on currently available evidence, to assess the costs and health benefits of an exemplar school-based, multi-component intervention to improve adolescents' diet and increase their levels of physical activity.
- The economic evaluation followed published best-practice guidance.
- In the absence of effectiveness results from the on-going EACH-B trial, the treatment effect of the intervention is obtained from a published systematic review.
- The model does not include long-term health outcomes, such as for cardiovascular diseases or different types of cancer.
- There remains uncertainty around key model assumptions related to the duration of benefits of the intervention.

Mesh terms: Adolescent, Cost-effectiveness, School-based, Exercise, Diet

For peer review only

For peer review only - http://bmjopen.bmj.com/site/about/guidelines.xhtml

INTRODUCTION

Poor diet and lack of physical activity increase the risk of non-communicable diseases (NCDs), including cardiovascular diseases, Type 2 diabetes and some cancers such as breast, colon and endometrial, in part by contributing to overweight and obesity.[1][2] Adolescence, the life stage between childhood and adulthood, is a critical period for the development of health and disease in later life.[3][4] Compared with other age-groups, adolescents have the unhealthiest diets and most (over 80%) fail to meet national guidelines for physical activity.[5-7] Furthermore, the proportion meeting recommended levels of physical activity has been declining, particularly among girls (from 14% to 8%, 2008-2012).[8]

The disease burden of poor diet and physical inactivity on healthcare services is significant. In the UK, poor diet, and physical inactivity cost £7 billion to the National Health Service (NHS) annually.[9] Meeting current dietary recommendations would reduce years-of-life lost to coronary heart disease by 2 million, stroke by 400,000 and Type 2 diabetes by 19,000 over 20 years.[10]

Health behaviours in adolescence track into adulthood.[5][9][11][12] Therefore, sub-optimal diet and body composition in adolescence not only affect immediate physical and mental health but also increase the risk of NCDs in later life. Developmental plasticity in adolescence means that interventions to improve diet and levels of physical activity have the potential to reduce the trajectory of NCD risk over the life course.[13] While many adolescents find it difficult to engage with the long-term consequences of health behaviour, motivated and engaged adolescents can improve their health behaviours.[14][15] Evidence suggests that school-based interventions that offer combinations of peer-modelling, social support and choice, may be effective in improving diet and physical activity amongst adolescents.[16-18] Furthermore there is an increasing use of digital platforms by adolescents. According to 2018 estimates, 83% of 12-15 years olds in the UK own smartphones, with 99% spending an average of 20 hours per week online.[19][20] With an explosion in the use of such platforms to influence health behaviours in young people, they have potential as a complementary feature in complex interventions that aim to influence health behaviour in adolescents.[20]

Within this framework, a research programme Engaging Adolescents in CHanging Behaviour

For peer review only - http://bmjopen.bmj.com/site/about/guidelines.xhtml

(EACH-B) was designed to develop and test an intervention to encourage UK-based school students, aged 12-13 years, to adopt healthy behaviours such as eating better and exercising more (Trial registration: ISRCTN 74109264). EACH-B involves a cluster randomised controlled trial as a test of intervention effectiveness. Further details of the trial design are given elsewhere.[20] The research programme is funded by the National Institute for Health Research (RP-PG-0216-20004). The "LifeLab Plus" intervention developed as part of this programme is a complex three-part programme that comprises: i) an education module that teaches school students the science behind health messages through a 2-week module with a "hands-on" practical one-day visit to a teaching laboratory at University Hospital Southampton or in school while COVID-19 restrictions apply; ii) training for teachers in skills to support behaviour change; and iii) access to a specially-designed, interactive smartphone app with game features.

There is an emerging interest in identifying and developing interventions for improving diet and physical activity levels in adolescents. Taking this into consideration, we developed an illustrative decision-analytic model to assess the health benefits, costs, and cost-effectiveness of a multi-component intervention such as LifeLab Plus. This prototype model is designed to investigate how changes in diet quality and physical activity could affect future health outcomes and costs. We used cost data from the EACH-B programme and effectiveness estimates for similar interventions from published literature. The aims of this paper are to describe the structure, assumptions and parameters for our prototype model, to present preliminary (prior) estimates of cost-effectiveness based on currently available information and explore the sensitivity of results to key uncertainties. The model will be updated when results from the EACH-B trial are available.

METHODS

We developed a prototype *de novo* Markov model to estimate the costs, benefits and cost-effectiveness of school-based interventions that aim to improve diet quality and levels of physical activity compared with usual schooling for a cohort of adolescents. The model focussed on four potential short to medium-term benefits of healthy eating and physical activity: better mental health (episodes of depression and generalised anxiety disorder), higher earnings and reduced incidence of Type 2 diabetes and adverse pregnancy outcomes

For peer review only - http://bmjopen.bmj.com/site/about/guidelines.xhtml

(in terms of pre-term delivery). The model assumed that improved diet quality and increased physical activity would impact these four aspects of health via a reduction in BMI. Discussion with key project stakeholders reiterated these four benefits as the most relevant obesity-related effects in this population.

The model did not include later life impacts on cardiovascular disease or other chronic diseases as the impact on these outcomes of an intervention undertaken as an adolescent is uncertain. The model also investigated independent effects of physical activity on diabetes and depression (i.e. direct impacts not mediated by BMI). Information relating to epidemiology, mortality, effectiveness, health-related quality-of-life and costs was obtained from a variety of sources to inform the model parameters and assumptions.

Structuring the model

Population

A cohort of 100 adolescents with an equal proportion of boys and girls and a mean age of 13 years entered the model. The exemplar intervention is based on LifeLab Plus[20] and compared with usual schooling.

Model states

The Markov model consisted of three health states as described below (Figure 1). Outcomes associated with mental health, loss of earnings and adverse pregnancy outcome were incorporated as model events.

- *Healthy*: Adolescents enter the model in this state where they are assumed to have no other comorbidities including Type 2 diabetes or mental health problems. In each model cycle, a proportion will develop Type 2 diabetes and move to the *'Diabetes'* state or they may experience- mental health events, -pre-term births (girls only) or -loss of earnings. The latter three are treated as transient states.
- *Diabetes:* A proportion of adolescents with Type 2 diabetes may enter the transient states, as stated above.
- *Death:* This is an absorbing state. Adolescents may transition to this state either through general population mortality or death from- mental health conditions or -Type 2 diabetes and related complications.

For peer review only - http://bmjopen.bmj.com/site/about/guidelines.xhtml

Mental health encompasses a wide spectrum of conditions. Therefore, a pragmatic approach was adopted to include the two most prevalent mental health conditions for adolescents: clinical depression (henceforth, referred as depression) and General Anxiety Disorder (GAD). These conditions were categorised as: chronic (history of persistent mental illness), intermittent (experiencing intermittent episodes), and new onset (a one-time episode). Adverse pregnancy outcome was defined by pre-term delivery categorised as: extremely pre-term (delivery <28 weeks); very pre-term (delivery <33 weeks); and moderately pre-term (delivery between 34-36 weeks). The pregnancy outcome was applicable for girls only.

Each health state and event are associated with an impact on health-related quality-of-life (HRQoL) and excess cost. The model included intervention cost associated with LifeLab Plus and healthcare costs associated with- Type 2 diabetes, mental health events, pre-term delivery and loss of earnings due to obesity. In each model cycle, the total costs and QALYs are calculated by multiplying the individual costs and HRQoL by the number of people in the cohort still alive for each of the intervention and control arms. The total lifetime costs and QALYs are calculated by aggregating the costs and QALYs for all cycles.

Persistence of effects and time horizon

Adolescents were assumed to receive the intervention at the model entry, the duration of which lasted for a year. Although there is good epidemiological evidence of long-term tracking of health behaviour,[5][9][11][12] school-based trials rarely follow up for more than a year.[1][21][22] The persistence of intervention effect in terms of behaviour change is therefore uncertain. Hence, we take a conservative approach and focus on potential impacts of improved diet and physical activity from such an intervention likely to manifest in the short to medium term: up to a maximum time horizon of 20 years. However, due to the uncertainty, the treatment effect on behaviour was not assumed to last for 20 years. Based on our experts' judgement, for the base case the effect was assumed to sustain for 4 years, waning to no effect over a period of 10 years. We explored alternative assumptions about the persistence of effects on behaviour after trial follow-up.

The model time horizon was 20 years with a yearly cycle length. The time horizon was considered appropriate as the mean age of adolescents entering the model was 13 years as in the EACH-B trial. Costs and outcomes were half-cycle corrected.

For peer review only

For peer review only - http://bmjopen.bmj.com/site/about/guidelines.xhtml

Populating the model

Targeted literature searches were conducted to identify sources to inform model parameters. These are discussed below and in Appendices A and B.

Epidemiological data

Data on the relationship between mean BMI, age and sex was taken from Health Survey for England.[23] For adolescents aged ≤19 years, BMI z-scores are normally used. Therefore, we rescaled the values to relate relative risks to BMI z-scores, where:

BMI z-score = (observed value – median value of the reference population) / standard deviation value of reference population.

Physical activity levels for children aged 13-15 years were taken from Health Survey England, 2015.[24] The incidence of Type 2 diabetes in the UK was based on an analysis of longitudinal electronic health records in the Health Improvement Network (THIN) primary care database.[25] The prevalence of depressive episodes and GAD was taken from the Adult Psychiatric Morbidity Survey 2014[26] and from Mental Health of Children and Young people in England 2017.[27] The proportion in each category was assumed as follows: 17% had a chronic and 40% had a fluctuating (intermittent) course, while 43% remitted (new one-time episode).[28] Those individuals with depression or anxiety are at higher risk of suicide than the general population.[29] The excess death rate for those with depression and anxiety was calculated by using the suicide rate in the UK from Office of National Statistics (ONS) 2017 and applying a relative risk of 10.9 for depression and anxiety.[29] The proportions of pre-term deliveries, obtained from ONS 2017 data, were assumed as follows: 0.5% of total births as extremely pre-term (<28 weeks), 1.2% very pre-term birth (28 to <33 weeks) and 6.3% moderately pre-term birth (33-36 weeks) respectively.[30]

Relationship between BMI and risks of health events

The economic model assumed a positive correlation between increased BMI and the risks of - Type 2 diabetes, depression and GAD, pre-term delivery and loss of earnings. We fitted equations to the BMI relative risks. Hazard ratios, obtained from the Medicare Current Beneficiary Survey 1991-2010, were used to estimate the increased risk of individuals with higher BMI developing Type 2 diabetes.[31] The odds of depression and GAD in obese and

For peer review only - http://bmjopen.bmj.com/site/about/guidelines.xhtml

overweight adolescents compared with normal-weight adolescents were obtained from Sutaria et al.[32] This systematic review included 22 observational studies published between 2000 and 2017, representing 143,603 children. The relative risk of pre-term birth for mothers with overweight and obesity was obtained from Mcdonald et al.[33] This was assumed to be the same for all three categories of pre-term births. The loss of earnings which is applied to change in BMI over time is estimated annually over the model time horizon. It is scaled according to the population age to factor in average income by age.

Relationship between physical activity and risk of health event

The direct effect of physical activity on developing depression is modelled independent of the effect of physical activity via BMI. The odds ratio of developing depression was assumed to be 0.83 (95% CI 0.79-0.88) in those with high levels of physical activity compared to those with lower levels.[34] Furthermore, an increase from being inactive to achieving 150 minutes of moderate-intensity physical activity per week was assumed to lower the risk of Type 2 diabetes incidence by 26%, after adjustment for body weight.[35] The pooled odds ratio between Type 2 diabetes and risk of depression was 1.33 (95% CI, 1.18-1.51).[36]

Intervention effect

The intervention effect was based on three systematic reviews and meta-analyses that estimated the overall effects of school-based obesity prevention interventions.[37-39] The results of the meta-analyses were found to be significantly different between groups based on BMI (− 0.17 (95% CI− 0.29, − 0.06) kg/m^2)[38] and BMI z-score (− 0.06 (95% CI -0.10, − 0.03))[39] for multi-component interventions including physical activity, health education and dietary improvement. The effect of school-based intervention was assumed to increase the level of moderate or vigorous physical activity in children and adolescents by 4.84 min/day (95% CI −0.94 to 10.61).[40]

Mortality

General population mortality, adjusted for age and gender, was based on ONS 2020.[41] A hazard ratio of mortality of 2.98 was applied for individuals with Type 2 diabetes and aged between 35 and <65 years.[42]

Quality-of-life

For peer review only - http://bmjopen.bmj.com/site/about/guidelines.xhtml

EQ-5D-3L estimates adjusted for age, gender and BMI were used to estimate quality-of-life (Table 1). These estimates were obtained from 14,117 participants aged ≥16 years from the Health Survey for England 2008.[43] Adults with diabetes were assumed to have a disutility of -0.161.[44] We estimated the disutility by comparing the mean general population EQ-5D score with that for people with diabetes. A disutility of -0.087 is used for adolescents with Type 2 diabetes based on a Swedish cohort of adolescents aged 13-18 years.[45] A utility decrement of 0.188 was assumed for those with intermittent episodes of mental health conditions;[46] a decrement of 0.488 for those with persistent/chronic depression;[47] a decrement of 0.094 (half of the decrement for intermittent depression) was assumed for those with a new episode of depression. For pre-term delivery, a mean utility decrement of 0.066 was applied throughout the model time horizon. This was based on a systematic review and meta-analysis for health utility values associated with pre-term birth where all but one study used Health Utilities Index (HUI) Mark 2 (HUI2) or Mark 3 (HUI3) measures as their primary health utility assessment method.[48] We found no evidence for quality-of-life loss in parents of pre-term babies. Therefore, we assumed that the quality-of-life decrement would be like intermittent mental health condition and lasts for the first two years.

Costs

We describe the costs below and summarise in Table 1.

Intervention costs

The intervention cost for our illustrative analysis was based on LifeLab Plus. Further information on resources used for delivering LifeLab are in Appendix B. The cost of the app delivered as part of the intervention, was incorporated as a capital cost and was assumed to last 10 years and be used in 10 centres. Similarly, the cost of setting up LifeLab Plus in a different centre was assumed to- consist of one year's staff costs and last for 10 years. Maintenance costs were estimated at 25% of the development cost per year. Overheads were included according to the rates used in Curtis et al. 2019[49]: direct overheads based on 29% of direct care salary costs and indirect overheads based on 16% direct care salary costs.

Health state costs

Both direct and indirect costs relating to individuals with Type 2 diabetes,[50] depression,[51] pre-term birth,[52] and loss of earnings were included. Indirect costs included

For peer review only - http://bmjopen.bmj.com/site/about/guidelines.xhtml

the effect of depression on income and productivity. Costs were updated to 2019 prices using the hospital and community health services index.[49]

Direct health costs and indirect societal and productivity costs were estimated using a top-down approach. We assumed that individuals with Type 2 diabetes would not incur costs for complications as these are likely to affect individuals who have diabetes for a longer period. For costs associated with pre-term delivery, total societal costs for children born at 32-33 weeks and 34-36 weeks gestation from birth to 24 months were based on a study that compared these costs to those for children born at full-term.[53] The difference in costs for children born <28 weeks and 28-33 weeks were assumed to be same when compared to those born at full-term.

The lifetime indirect costs for overweight and obesity in childhood and adolescence is based on a study by Hamilton et al.[54] Mean total lifetime healthcare and productivity costs were estimated in Irish Euros, which were converted to GBP (£) and adjusted according to the average wage in the UK.

Validation

The structure of the prototype model was validated by the study team comprising epidemiologists, statisticians, trialists, public health experts and health economists. Internal validity of the model was established ensuring that the model predictions were consistent with the model specification.

Cost-effectiveness analysis

Costs and effects were estimated in accordance with the NICE Reference case,[55] except for the perspective adopted; we used a societal perspective that included both direct and indirect costs. Costs and health effects (QALYs) were discounted at 3.5% per year. The incremental cost-effectiveness ratio (ICER) was estimated as a ratio of the incremental costs of the intervention (LifeLab Plus) relative to the comparator (usual schooling) to the incremental QALYs of the intervention relative to the comparator. The intervention was considered cost-effective if the ICER was below the threshold of £20,000 per QALY gained, the lower threshold usually considered for the English NHS by NICE.[55]

Sensitivity analyses

For peer review only - http://bmjopen.bmj.com/site/about/guidelines.xhtml

To assess the uncertainty around the model predictions, we conducted deterministic sensitivity analysis (DSA), probabilistic sensitivity analyses (PSA) and scenario analyses. For the DSA, input parameters were varied by 95% confidence intervals (where available) or varied by 10% of the mean. Monte Carlo simulations of 10,000 iterations were run for the PSA to assess the combined effects of input parameter uncertainties where parameters were simultaneously sampled within a specified distribution. We used standard distributions for the PSA, as recommended by Briggs et al.[56]: effectiveness parameters were assigned beta and lognormal distributions; utilities were assumed to follow a beta distribution; and costs were assigned a gamma distribution (Table 1 and further details in Appendix A). Scenario analyses were conducted to assess structural uncertainties related to model assumptions. Uncertainty about the sustainability of the intervention effect was assessed by varying the duration of intervention effect and its waning period.

The model was developed and implemented in Microsoft Excel.

Patient and public involvement

The research questions addressed in the overarching research programme EACH-B were informed by public involvement. Furthermore, representatives from public involved in the EACH-B research programme were presented the conceptual framework, modelling approaches, and invited to comment.

RESULTS

Base case analysis

For the base case, the exemplar intervention based on the multi-component LifeLab Plus was associated with higher costs and better health outcomes (more QALYs) compared with usual schooling. LifeLab Plus was associated with a mean QALY gain of 0.0085 at an incremental cost of £123 per person compared with usual schooling, resulting in an ICER of £14,367 per QALY (Table 2).

Sensitivity analyses

The results of the one-way sensitivity analyses are presented in the Tornado plot (Figure 2). Parameters including the intervention effect on levels of physical activity (expressed in terms

For peer review only - http://bmjopen.bmj.com/site/about/guidelines.xhtml

of minutes of moderate or vigorous physical activity), quality-of-life gain for high levels of physical activity, duration of the intervention effect and duration of the treatment waning period had the highest impact on the cost-effectiveness results. Other parameters such as effect of physical activity on BMI, time horizon and intervention costs also influenced the base case results, but to a lesser extent. The results of the PSA are presented as a cost-effectiveness scatter plot in Figure 3. All the iterations lie in the North-East quadrant of the cost-effectiveness plane, thereby indicating that the intervention is likely to produce health benefits at an additional cost. At a willingness-to-pay threshold of £20,000 per QALY gained, the probability of LifeLab Plus being cost-effective was 69% compared with usual schooling (Appendix C).

DISCUSSION

Main findings

Based on our prototype economic model, we estimate that a multi-component intervention to improve dietary quality and physical activity, such as LifeLab Plus, is likely to be considered cost-effective under conventional willingness-to-pay thresholds of £20,000–£30,000 per QALY gained in the UK. For our base case, the duration of the treatment effect was assumed to sustain for 4 years with the effect waning over a further period of 10 years. Our sensitivity analyses showed that if the duration of the treatment effect was not sustained to this extent, the intervention would be less cost-effective (Table 2). Changes to physical activity had a greater effect on the model results than changes in BMI. This is largely because physical activity is associated with an additional improvement in quality-of-life, independent of any health benefits associated with the health states in our model.

Comparison with previous models

Previous cost-effectiveness studies evaluating dietary and physical activity interventions for adolescents have largely consisted of within-trial analyses that have not considered the benefits beyond the trial period.[57-61] Cost-effectiveness estimates vary between a cost saving of £408.22 (NZ$835) per child for a low intensity program[57] to an additional cost of £120,630 per QALY gained for a more intensive intervention.[60] However, comparison between studies is difficult because of the differences in- the study designs, the interventions considered and the outcomes reported.

For peer review only - http://bmjopen.bmj.com/site/about/guidelines.xhtml

Gc et al.[1] developed a model to assess the long-term costs and health outcomes of two physical activity interventions targeting adolescents in the UK. The cost-effectiveness estimates varied from £11,426 per QALY for an after-school intervention to £68,056 per QALY for a multicomponent intervention. The costs of these interventions were estimated at £51 per participant for the after-school intervention and £207 (AUS$394) for the multicomponent intervention. Their model included different health states to our model for diseases which typically affect people in later life including chronic heart disease, stroke, heart failure, breast failure and colorectal cancer. They ran the model for a lifetime horizon of 65 years. We have not included these health states as we adopted a conservative assumption that treatment benefits for adolescents from such multicomponent intervention as LifeLab Plus do not persist beyond 20 years.

Strengths and limitations

The study addresses an important public health question by examining whether interventions targeting healthy eating and increased physical activity for adolescents provide value for money from a societal perspective. We incorporate existing evidence on the effect of improvement in adolescent health behaviours on four high prevalence short-to-medium term benefits relevant to adolescents: improved mental health, higher earnings, improved pregnancy outcomes and prevention of Type 2 diabetes. Sources for data used within our model were identified from a targeted literature review. Where data were not available for adolescents, we used data from adult population. Model structure and assumptions were informed by this review and discussions with public health experts. UK-specific incidence rates were used to ensure that patients entering the model matched the likely distribution of events in the UK. Our analysis focuses on a pragmatic subset of specific outcomes, while noting these could be expanded on with emerging data on effectiveness, notably in relation to mental health and other long-term conditions.

We do not attempt to estimate the long-term impacts of such interventions due to the lack of longitudinal data on lifetime trajectories of healthy diet and increased levels of physical activity. Our preliminary model suggests that this type of intervention is likely to be cost-effective based on short-to-medium term effects alone. If this is correct and the effects last longer, then additional benefits such as prevention of cardiovascular disease would enhance the cost-effectiveness of these interventions. This suggests that our cost-effectiveness results are conservative, and that quantification of long-term benefits is not necessary to demonstrate

For peer review only - http://bmjopen.bmj.com/site/about/guidelines.xhtml

the cost-effectiveness of this type of intervention. However, the sensitivity analyses reported above do indicate some important uncertainties.

Our model has several shortcomings. The EACH-B study, which is the main trial evidence, is ongoing so there is currently no direct data on the effectiveness of this intervention. We have used evidence on treatment effectiveness from a systematic review of related interventions. When results become available from the EACH-B study, they will add to the evidence base and reduce uncertainty in these results. Further evidence for other model parameters would also be valuable. For example, where data from adolescent age groups were not available, we used epidemiological estimates from adult populations that might not be transferable. There is uncertainty around key assumptions related to the duration of benefits from the intervention. In our analysis, we have assumed that the benefit observed in the clinical trials will last for 4 years and then will gradually reduce over the next 10 years. We only include costs and effects over a 20-year time horizon and have assumed that there would be no further benefits of the intervention for chronic diseases such as cardiovascular diseases and diabetes. There are also limitations in our approach to estimating income lost due to obesity. We have adopted a simple approach; however, this is a complex interacting bi-directional system. We have not fully explored whether income loss is due to obesity or whether obesity is caused by income loss through, for example, unemployment. Other broader uncertainties, such as long-term effects of COVID on behaviour and mental health are also not addressed.

CONCLUSION

Complex behavioural interventions that aim to improve diet and increase levels of physical activity amongst school-aged children have the potential to provide cost-effective use of UK healthcare resources. Such interventions have the potential to reduce burden of NCDs, although benefits in later life are more sensitive to assumptions about the persistence of behavioural change and discounting. Our analysis focuses on a pragmatic subset of specific outcomes based on published literature on effectiveness of similar interventions, while noting these could be expanded on with emerging data on effectiveness, notably in relation to mental health and other long-term conditions.

Word count: 4222

For peer review only - http://bmjopen.bmj.com/site/about/guidelines.xhtml

Author Contributions NK designed the model, performed the analysis, interpreted the analysis, and wrote the first draft of the manuscript. KC designed the model and performed the analysis. JL supervised the process, provided comments on model structure and data analysis. KWT provided inputs for model parameters. JB, KMG, HI, MB and CC provided critical comments on the model structure. All authors contributed to the critical revision of the manuscript and approved the final version of the manuscript.

Ethics approval Not required as the model is informed by data from the published literature.

Funding The research is funded by the National Institute for Health Research (RP-PG-0216-20004). HMI and KMG are funded by the UK Medical Research Council (MC_UU_12011/4). KMG is supported by the National Institute for Health Research (NIHR Senior Investigator (NF-SI-0515-10042), NIHR Southampton 1000DaysPlus Global Nutrition Research Group (17/63/154) and NIHR Southampton Biomedical Research Centre (IS-BRC-1215-20004)), the European Union (Erasmus+ Programme ImpENSA 598488-EPP-1-2018-1-DE-EPPKA2-CBHE-JP) and the British Heart Foundation (RG/15/17/3174).

Competing Interests KMG has received reimbursement for speaking at conferences sponsored by companies selling nutritional products and is part of an academic consortium that has received research funding from Abbott Nutrition, Nestec, BenevolentAI Bio Ltd. and Danone. The other authors have no potentially competing interests to declare.

Patient consent for publication Not required.

Data availability statement Data from publicly available sources were used for the model. All model inputs are described in the paper.

For peer review only

For peer review only - http://bmjopen.bmj.com/site/about/guidelines.xhtml

Table 1 Input parameters used in the model

Parameter	Value	SD/SE	95% CI-Low	95% CI-High	Distribution	Reference
**Utility inputs**						
Disutility for Diabetes for 13-18 years old	-0.087	0.036	0.016	0.158	Beta	[45]
Disutility for Diabetes	-0.161	0.040	0.240	0.080	Beta	[44]
Disutility for pre-term babies < 33 weeks	-0.066	0.016	0.098	0.035	Beta	[48]
Disutility for parents of pre-term babies < 33 weeks	-0.094	0.019	0.057	0.131	Beta	Assumption
Disutility for depression	-0.188	0.038	0.114	0.262	Beta	[46]
Disutility for Chronic mental health condition	-0.448	0.036	0.377	0.519	Beta	[47]
Disutility for Intermittent mental health condition	-0.188	0.038	0.114	0.262	Beta	[46]
Disutility for New cases mental health condition	-0.094	0.019	0.057	0.131	Beta	Assumption
Quality-of-life gain for high activity	0.020	0.002	0.016	0.024	Beta	
**Direct costs**						
Type 2 diabetes	£622	£62	£500	£744	Gamma	[50]
Mental health condition	£1,334	£383	£585	£2,307	Gamma	[51]
Mental health condition- chronic	£4,233	£629	£3,001	£7,238	Gamma	[51]

For peer review only

Mental health condition- intermittent	£1,334	£383	£585	£2,307	Gamma	[51]
Mental health condition-new onset	£667	£191	£292	£1,042	Gamma	[51]
Pre-term birth <28 weeks	£25,452	£2,169	£21,201	£29,704	Gamma	[52][53]
Pre-term birth 28-33 weeks	£13,073	£1,114	£10,889	£15,256	Gamma	[52][53]
Pre-term birth, 34-36 weeks	£4,157	£1,020	£2,157	£6,157	Gamma	[52][53]
**Indirect costs**						
Type 2 diabetes	£4,116	£412	£3,309	£4,923	Gamma	[50]
Mental health condition	£223	£64	£98	£348	Gamma	[51]
Mental health condition- chronic	£707	£203	£310	£1,105	Gamma	[51]
Mental health condition- intermittent	£223	£64	£98	£348	Gamma	[51]
Mental health condition-new onset	£112	£32	£49	£174	Gamma	[51]
Pre-term birth <28 weeks	£397	£114	£174	£619	Gamma	[52][53]
Pre-term birth 28-33 weeks	£119	£34	£52	£185	Gamma	[52][53]
Pre-term birth, 34-36 weeks	£103	£29	£45	£161	Gamma	[52][53]
**Cost of the intervention**	£155	£15	£124	£185	Gamma	Estimated

SD: Standard Deviation; SE: Standard Error; CI: Confidence Interval

For peer review only - http://bmjopen.bmj.com/site/about/guidelines.xhtml

Table 2 Incremental cost-effectiveness ratios in the base case and scenario analyses					
	**Total cost (£)**	**Total QALYs**	**Incremental cost (£)**	**Incremental QALY**	**ICER**
**Base case analysis**					
Usual schooling	£4,416	13.07			
LifeLab Plus	£4,539	13.08	£123	0.0085	£14,367
**Perspective: NHS**					
Usual schooling	£2,001	13.07			
LifeLab Plus	£2,141	13.08	£140	0.0085	£16,339
**Costs discounted at 3.5%; QALYs at 1.5% per annum**					
Usual schooling	£4,416	15.76			
LifeLab Plus	£4,539	15.77	£123	0.0097	£12,640
**Costs and QALYs discounted at 1.5%**					
Usual schooling	£5,813	15.76			
LifeLab Plus	£5,930	15.77	£117	0.0097	£12,044
**Time Horizon: 5 years**					
Usual schooling	£488	4.25			
LifeLab Plus	£633	4.25	£145	0.0043	£33,423
**Time Horizon: 10 years**					
Usual schooling	£1,132	7.76			
LifeLab Plus	£1,265	7.77	£134	0.0071	£18,851
**Time Horizon: 30 years**					
Usual schooling	£11,137	16.67			
LifeLab Plus	£11,258	16.67	£121	0.0088	£13,719
**Time Horizon: 40 years**					
Usual schooling	£18,417	19.05			
LifeLab Plus	£18,537	19.06	£120	0.0089	£13,455
**Duration of treatment effect: 1 year & Treatment waning: 2 years**					
Usual schooling	£4,416	13.07			
LifeLab Plus	£4,566	13.08	£150	0.0030	£49,758
**Duration of treatment effect: 1 years & Treatment waning: 5 years**					
Usual schooling	£4,416	13.07			
LifeLab Plus	£4,563	13.08	£147	0.0043	£34,089
**Duration of treatment effect: 2 years & Treatment waning: 5 years**					
Usual schooling	£4,416	13.07			
LifeLab Plus	£4,560	13.08	£144	0.0051	£28,034
**Duration of treatment effect: 5 years & Treatment waning: 5 years**					
Usual schooling	£4,416	13.07			
LifeLab Plus	£4,553	13.08	£137	0.0075	£18,218
**Duration of treatment effect: 5 years & Treatment waning: 10 years**					
Usual schooling	£4,416	13.07			
LifeLab Plus	£4,529	13.08	£113	0.0092	£12,184
**Duration of treatment effect: 10 years & Treatment waning: 10 years**					
Usual schooling	£4,416	13.07			
LifeLab Plus	£4,432	13.09	£16	0.0125	£1,257
**Duration of treatment effect: 15 years & Treatment waning: 5 years**					
Usual schooling	£4,416	13.07			

For peer review only - http://bmjopen.bmj.com/site/about/guidelines.xhtml

Table 2 Incremental cost-effectiveness ratios in the base case and scenario analyses

	Total cost (£)	Total QALYs	Incremental cost (£)	Incremental QALY	ICER
LifeLab Plus	£4,308	13.09	-£108	0.0147	Dominates

ICER, incremental cost-effectiveness ratio (incremental cost/incremental QALY); QALY, quality-adjusted life year.

List of Figure legends

- Figure 1 provides an illustration of the conceptual framework for the cost-effective model and an illustration of the model structure that shows transition of the modelled population through the modelled health states.
- Figure 2 provides an illustration of the Tornado diagram obtained from the deterministic sensitivity analysis that shows the impact of varying the input values of the parameters that have the highest impact on the cost-effectiveness result.
- Figure 3 provides an illustration of the scatter plot obtained from the probabilistic sensitivity analysis. Results from all the 10,000 simulations fall within the North-East quadrant of the cost-effectiveness plan thereby indicating that the intervention (LifeLab Plus) is more expensive and more effective compared to usual schooling.

For peer review only

REFERENCES

1. Gc VS, Suhrcke M, Atkin AJ, et al. Cost-effectiveness of physical activity interventions in adolescents: model development and illustration using two exemplar interventions. *BMJ Open* 2019;9(8):e027566. doi: 10.1136/bmjopen-2018-027566 [published Online First: 2019/08/21]
2. Organisation WH. Unhealthy Diets & Physical Inactivity 2009 [Available from: https://www.who.int/nmh/publications/fact_sheet_diet_en.pdf accessed 19/11/2020.
3. Organisation WH. Adolescent Health 2020 [Available from: https://www.who.int/health-topics/adolescent-health/#tab=tab_1 accessed 13/11/20.
4. Viner RM, Ross D, Hardy R, et al. Life course epidemiology: recognising the importance of adolescence. *Journal of Epidemiology and Community Health* 2015;69(8):719-20. doi: 10.1136/jech-2014-205300
5. Rose T, Barker M, Maria Jacob C, et al. A Systematic Review of Digital Interventions for Improving the Diet and Physical Activity Behaviors of Adolescents. *J Adolesc Health* 2017;61(6):669-77. doi: 10.1016/j.jadohealth.2017.05.024 [published Online First: 2017/08/16]
6. Bates B, Lennox A, Prentice A, et al. National Diet and Nutrition Survey: Results from Years 1, 2, 3 and 4 (combined) of the Rolling Programme (2008/2009-2011/2012): a Survey Carried Out on Behalf of Public Health England and the Food Standards Agency: Public Health England, 2014.
7. Woods-Townsend K, Bagust L, Barker M, et al. Engaging teenagers in improving their health behaviours and increasing their interest in science (Evaluation of LifeLab Southampton): study protocol for a cluster randomized controlled trial. *Trials* 2015;16:372-72. doi: 10.1186/s13063-015-0890-z
8. NHS. Health Survey for England 2015 Physical activiy in children 2016 [Available from: http://healthsurvey.hscic.gov.uk/media/37752/hse2015-child-phy-act.pdf accessed 17/11/20.
9. term TNL. 2019 [Available from: https://www.longtermplan.nhs.uk/publication/nhs-long-term-plan accessed 15/09/20.
10. Milner J, Green R, Dangour AD, et al. Health effects of adopting low greenhouse gas emission diets in the UK. *BMJ Open* 2015;5(4):e007364. doi: 10.1136/bmjopen-2014-007364
11. Kelder SH, Perry CL, Klepp KI, et al. Longitudinal tracking of adolescent smoking, physical activity, and food choice behaviors. *Am J Public Health* 1994;84(7):1121-26. doi: 10.2105/ajph.84.7.1121
12. Craigie AM, Lake AA, Kelly SA, et al. Tracking of obesity-related behaviours from childhood to adulthood: A systematic review. *Maturitas* 2011;70(3):266-84. doi: 10.1016/j.maturitas.2011.08.005 [published Online First: 2011/09/17]
13. Hanson M, Gluckman P. Developmental origins of noncommunicable disease: population and public health implications. *Am J Clin Nutr* 2011;94(6 Suppl):1754s-58s. doi: 10.3945/ajcn.110.001206 [published Online First: 2011/04/29]
14. Gates M. Advancing the adolescent health agenda. *The Lancet* 2016;387(10036):2358-59. doi: 10.1016/S0140-6736(16)30298-7
15. Organisation WH. Global Accelerated Action for the Health of Adolescents (AAHA!): guidance to support country implementation. Geneva, 2017.
16. Kelishadi R, Azizi-Soleiman F. Controlling childhood obesity: A systematic review on strategies and challenges. *J Res Med Sci* 2014;19(10):993-1008.
17. Calvert S, Dempsey RC, Povey R. Delivering in-school interventions to improve dietary behaviours amongst 11- to 16-year-olds: A systematic review. *Obesity Reviews* 2019;20(4):543-53. doi: https://doi.org/10.1111/obr.12797

For peer review only - http://bmjopen.bmj.com/site/about/guidelines.xhtml

18. Woods-Townsend K, Hardy-Johnson P, Bagust L, et al. A cluster-randomised controlled trial of the LifeLab education intervention to improve health literacy in adolescents. *PLoS One* in press
19. Ofcom. Children and parents: Media use and attitudes report 2019 [Available from: https://www.ofcom.org.uk/__data/assets/pdf_file/0023/190616/children-media-use-attitudes-2019-report.pdf accessed 10/09/20.
20. Strömmer S, Barrett M, Woods-Townsend K, et al. Engaging adolescents in changing behaviour (EACH-B): a study protocol for a cluster randomised controlled trial to improve dietary quality and physical activity. *Trials* 2020;21(1):859. doi: 10.1186/s13063-020-04761-w
21. Borde R, Smith JJ, Sutherland R, et al. Methodological considerations and impact of school-based interventions on objectively measured physical activity in adolescents: a systematic review and meta-analysis. *Obesity Reviews* 2017;18(4):476-90. doi: https://doi.org/10.1111/obr.12517
22. Buxton MJ, Drummond MF, Van Hout BA, et al. Modelling in Ecomomic Evaluation: An Unavoidable Fact of Life. *Health Economics* 1997;6(3):217-27. doi: https://doi.org/10.1002/(SICI)1099-1050(199705)6:3<217::AID-HEC267>3.0.CO;2-W
23. NHS Digital. Health Survey for England 2016. 2017.
24. Digital N. Health Survey for England 2015 [Available from: https://digital.nhs.uk/data-and-information/publications/statistical/health-survey-for-england/health-survey-for-england-20152020.
25. Sharma M, Nazareth I, Petersen I. Trends in incidence, prevalence and prescribing in type 2 diabetes mellitus between 2000 and 2013 in primary care: a retrospective cohort study. *BMJ Open* 2016;6(1):e010210. doi: 10.1136/bmjopen-2015-010210 [published Online First: 2016/01/16]
26. McManus S BP, Jenkins R, Brugha T. Mental health and wellbeing in England: Adult Psychiatric Morbidity Survey 2014. 2016.
27. NHS Digital. Mental health of children and young people in England, 2017. 2017.
28. Stegenga BT, Kamphuis MH, King M, et al. The natural course and outcome of major depressive disorder in primary care: the PREDICT-NL study. *Soc Psychiatry Psychiatr Epidemiol* 2012;47(1):87-95. doi: 10.1007/s00127-010-0317-9 [published Online First: 2010/11/09]
29. Singhal A, Ross J, Seminog O, et al. Risk of self-harm and suicide in people with specific psychiatric and physical disorders: comparisons between disorders using English national record linkage. *J R Soc Med* 2014;107(5):194-204. doi: 10.1177/0141076814522033 [published Online First: 2014/02/15]
30. Office of National Statistics. Births in England and Wales: 2017. 2018.
31. Gray N, Picone G, Sloan F, et al. Relation between BMI and diabetes mellitus and its complications among US older adults. *South Med J* 2015;108(1):29-36. doi: 10.14423/SMJ.0000000000000214 [published Online First: 2015/01/13]
32. Sutaria S, Devakumar D, Yasuda SS, et al. Is obesity associated with depression in children? Systematic review and meta-analysis. *Arch Dis Child* 2019;104(1):64-74. doi: 10.1136/archdischild-2017-314608 [published Online First: 2018/07/01]
33. McDonald SD, Han Z, Mulla S, et al. Overweight and obesity in mothers and risk of preterm birth and low birth weight infants: systematic review and meta-analyses. *BMJ* 2010;341:c3428. doi: 10.1136/bmj.c3428 [published Online First: 2010/07/22]
34. Schuch FB, Vancampfort D, Firth J, et al. Physical Activity and Incident Depression: A Meta-Analysis of Prospective Cohort Studies. *Am J Psychiatry* 2018;175(7):631-48. doi: 10.1176/appi.ajp.2018.17111194 [published Online First: 2018/04/25]

For peer review only - http://bmjopen.bmj.com/site/about/guidelines.xhtml

35. Wahid A, Manek N, Nichols M, et al. Quantifying the Association Between Physical Activity and Cardiovascular Disease and Diabetes: A Systematic Review and Meta‐Analysis. *Journal of the American Heart Association* 2016;5(9) doi: 10.1161/jaha.115.002495
36. Chireh B, Li M, D'Arcy C. Diabetes increases the risk of depression: A systematic review, meta-analysis and estimates of population attributable fractions based on prospective studies. *Prev Med Rep* 2019;14:100822. doi: 10.1016/j.pmedr.2019.100822 [published Online First: 2019/03/01]
37. Liu Z, Xu HM, Wen LM, et al. A systematic review and meta-analysis of the overall effects of school-based obesity prevention interventions and effect differences by intervention components. *Int J Behav Nutr Phys Act* 2019;16(1):95. doi: 10.1186/s12966-019-0848-8 [published Online First: 2019/10/31]
38. Lavelle HV, Mackay DF, Pell JP. Systematic review and meta-analysis of school-based interventions to reduce body mass index. *J Public Health (Oxf)* 2012;34(3):360-9. doi: 10.1093/pubmed/fdr116 [published Online First: 2012/01/24]
39. Jacob CM, Hardy-Johnson PL, Inskip HM, et al. A systematic review and meta-analysis of school-based interventions with health education to reduce body mass index in adolescents aged 10 to 19 years. *Int J Behav Nutr Phys Act* 2021;18(1):1. doi: 10.1186/s12966-020-01065-9 [published Online First: 2021/01/06]
40. Mears R, Jago R. Effectiveness of after-school interventions at increasing moderate-to-vigorous physical activity levels in 5- to 18-year olds: a systematic review and meta-analysis. *Br J Sports Med* 2016;50(21):1315-24. doi: 10.1136/bjsports-2015-094976 [published Online First: 2016/05/26]
41. Statistics OfN. National life tables: England. 2020. https://www.ons.gov.uk/peoplepopulationandcommunity/birthsdeathsandmarriages/lifeexpectancies/datasets/nationallifetablesenglandreferencetables (accessed 22/03/2021).
42. Mulnier HES, H.E.;Raleigh,V.S.;Soedamah-Muthu,S.S.;Colhoun,H.M.;Lawrenson,R.A.;. Mortality in people with Type 2 diabetes in the UK. *Diabetic Medicine* 2006(23):516-21.
43. Maheswaran H, Petrou S, Rees K, et al. Estimating EQ-5D utility values for major health behavioural risk factors in England. *J Epidemiol Community Health* 2013;67(2):172-80. doi: 10.1136/jech-2012-201019 [published Online First: 2012/07/31]
44. Janssen MF, Lubetkin EI, Sekhobo JP, et al. The use of the EQ-5D preference-based health status measure in adults with Type 2 diabetes mellitus. *Diabet Med* 2011;28(4):395-413. doi: 10.1111/j.1464-5491.2010.03136.x [published Online First: 2011/03/12]
45. Astrom M, Persson C, Linden-Bostrom M, et al. Population health status based on the EQ-5D-Y-3L among adolescents in Sweden: Results by sociodemographic factors and self-reported comorbidity. *Qual Life Res* 2018;27(11):2859-71. doi: 10.1007/s11136-018-1985-2 [published Online First: 2018/09/10]
46. Lynch FD, JF.; Feeny, DH.; Clarke, GN.; MacMillan, AL.;. Measuring Health-related Quality of Life in Teens With and Without Depression. *Medical Care* 2016;54(12):8.
47. Morriss R, Garland A, Nixon N, et al. Efficacy and cost-effectiveness of a specialist depression service versus usual specialist mental health care to manage persistent depression: a randomised controlled trial. *The Lancet Psychiatry* 2016;3(9):821-31. doi: 10.1016/s2215-0366(16)30143-2
48. Petrou S, Krabuanrat N, Khan K. Preference-Based Health-Related Quality of Life Outcomes Associated with Preterm Birth: A Systematic Review and Meta-analysis.

For peer review only - http://bmjopen.bmj.com/site/about/guidelines.xhtml

Pharmacoeconomics 2019 doi: 10.1007/s40273-019-00865-7 [published Online First: 2019/12/10]

49. Curtis LB, A.;. Unit costs of Health and Social Care 2018. 2018.
50. Hex N, Bartlett C, Wright D, et al. Estimating the current and future costs of Type 1 and Type 2 diabetes in the UK, including direct health costs and indirect societal and productivity costs. *Diabet Med* 2012;29(7):855-62. doi: 10.1111/j.1464-5491.2012.03698.x [published Online First: 2012/04/28]
51. Kuyken W, Hayes R, Barrett B, et al. Effectiveness and cost-effectiveness of mindfulness-based cognitive therapy compared with maintenance antidepressant treatment in the prevention of depressive relapse or recurrence (PREVENT): a randomised controlled trial. *The Lancet* 2015;386(9988):63-73. doi: 10.1016/s0140-6736(14)62222-4
52. Khan KA, Petrou S, Dritsaki M, et al. Economic costs associated with moderate and late preterm birth: a prospective population-based study. *BJOG* 2015;122(11):1495-505. doi: 10.1111/1471-0528.13515 [published Online First: 2015/07/30]
53. Mangham LJ, Petrou S, Doyle LW, et al. The cost of preterm birth throughout childhood in England and Wales. *Pediatrics* 2009;123(2):e312-27. doi: 10.1542/peds.2008-1827 [published Online First: 2009/01/28]
54. Hamilton D, Dee A, Perry IJ. The lifetime costs of overweight and obesity in childhood and adolescence: a systematic review. *Obes Rev* 2018;19(4):452-63. doi: 10.1111/obr.12649 [published Online First: 2017/12/23]
55. The National Institute for Health and Care Excellence. Guide to the methods of technology appraisal, 2013.
56. Briggs AH, Claxton K, Sculpher MJ. Decision Modelling for Health Economic Evaluation - Handbooks in Health Economic Evaluation Series. Oxford University Press. 2006.
57. Anderson YC, Leung W, Grant CC, et al. Economic evaluation of a multi-disciplinary community-based intervention programme for New Zealand children and adolescents with obesity. *Obesity Research & Clinical Practice* 2018;12(3):293-98. doi: https://dx.doi.org/10.1016/j.orcp.2018.04.001
58. Ladapo JA, Bogart LM, Klein DJ, et al. Cost and Cost-Effectiveness of Students for Nutrition and eXercise (SNaX). *Academic pediatrics* 2016;16(3):247-53. doi: https://dx.doi.org/10.1016/j.acap.2015.07.009
59. Lee BY, Adam A, Zenkov E, et al. Modeling The Economic And Health Impact Of Increasing Children's Physical Activity In The United States. *Health Affairs* 2017;36(5):902-08. doi: https://dx.doi.org/10.1377/hlthaff.2016.1315
60. Panca M, Christie D, Cole TJ, et al. Cost-effectiveness of a community-delivered multicomponent intervention compared with enhanced standard care of obese adolescents: cost-utility analysis alongside a randomised controlled trial (the HELP trial). *BMJ Open* 2018;8(2):e018640. doi: https://dx.doi.org/10.1136/bmjopen-2017-018640
61. Sutherland R, Reeves P, Campbell E, et al. Cost-effectiveness of a multi-component school-based physical activity intervention targeting adolescents: the 'Physical Activity 4 Everyone' cluster randomized trial. *International Journal of Behavioral Nutrition & Physical Activity* 2016;13:94. doi: https://dx.doi.org/10.1186/s12966-016-0418-2

For peer review only - http://bmjopen.bmj.com/site/about/guidelines.xhtml

For peer review only

For peer review only - http://bmjopen.bmj.com/site/about/guidelines.xhtml

Figure 1: Illustration of the conceptual framework and model structure

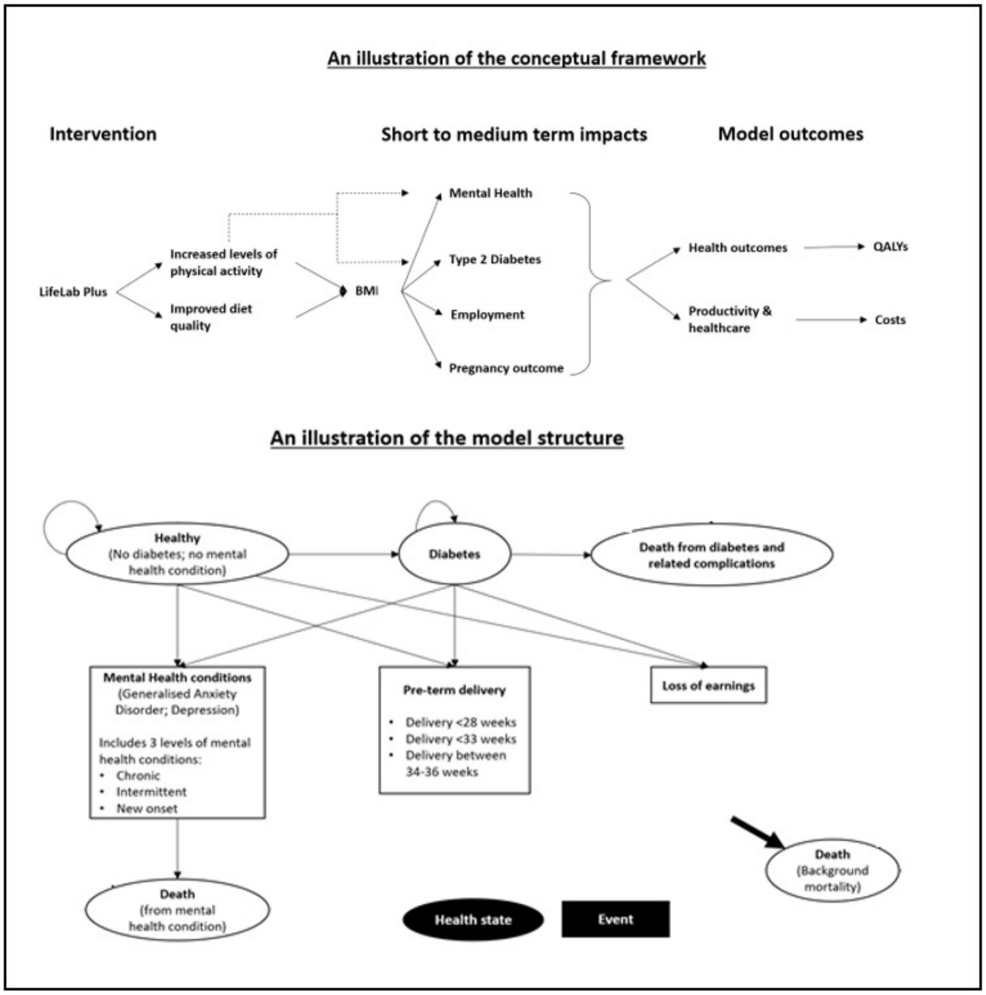

• Figure 1 provides an illustration of the conceptual framework for the cost-effective model and an illustration of the model structure that shows transition of the modelled population through the modelled health states.

137x145mm (144 x 144 DPI)

Figure 2: Tornado diagram to show the impact of varying parameter input values on ICERs

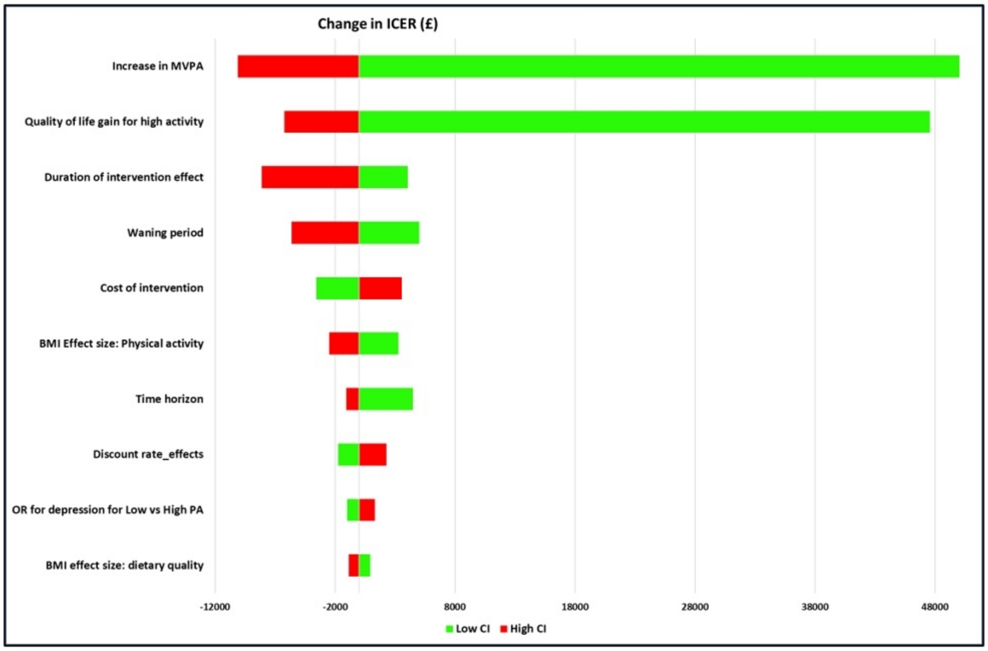

MVPA: Moderate or Vigorous Physical Activity; BMI: Body Mass Index; CI: Confidence Interval; ICER: Incremental Cost Effectiveness Ratio

• Figure 2 provides an illustration of the Tornado diagram obtained from the deterministic sensitivity analysis that shows the impact of varying the input values of the parameters that have the highest impact on the cost-effectiveness result.

175x127mm (144 x 144 DPI)

Figure 3: Scatter plot of PSA cost-effectiveness results

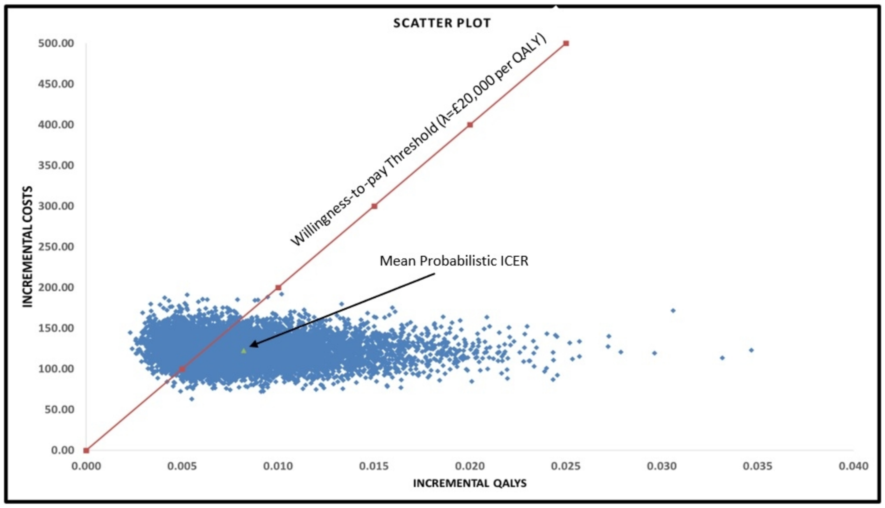

The probabilistic model was run for 10,000 simulations. All the plots fall in the North-East quadrant of the Cost-effectiveness plane, indicating the intevention- LifeLab Plus is associated with higher costs and higher QALYs compared to usual schooling. Majority of the plots, including the mean ICER, fall within the willingness-to-pay threshold of £20,000 per QALY.

• Figure 3 provides an illustration of the scatter plot obtained from the probabilistic sensitivity analysis. Results from all the 10,000 simulations fall within the North-East quadrant of the cost-effectiveness plan thereby indicating that the intervention (LifeLab Plus) is more expensive and more effective compared to usual schooling.

199x122mm (144 x 144 DPI)

1 APPENDIX A

Model parameters were estimated by using the following equations. For the control arm, rate was based upon age only. For the intervention arm, rate was based on age and the change in BMI from the control population to the intervention.

Table 1: Parameter equations

**Parameter**	**Equation**	**Units**	**Source**
BMI - men	-0.0031*age^2+0.4096*age+16.098	Kg/m2	HSE 2017[1]
BMI - women	-0.0032*Age^2 + 0.4235*Age+15.812	Kg/m2	
Type 2 Diabetes Men Control, age <25y	0.025*Age-0.265	Incidence per 1000	Sharma 2015[2]
Type 2 Diabetes Men Control, age >25y	0.0054Age^2-0.191Age+1.7142	Incidence per 1000	
Type 2 Diabetes Women, Control, all ages	0.0031Age^2-0.0942Age+1.2045	Incidence per 1000	
Type 2 Diabetes Men, Intervention, age < 25y	(0.025*Age-0.265)/(0.081BMI_control-0.8451)*(0.081BMI_int-0.8451)	Incidence per 1000	Gray et al 2015[3]
Type 2 Diabetes Men, Intervention, > 25y	(0.0054Age^2-0.191Age+1.7142)/(0.081BMI_control-0.8451)*(0.081BMI_int-0.8451)	Incidence per 1000	
Type 2 Diabetes Women Intervention, all ages	(0.0031Age^2-0.0942Age+1.2045)/(0.0637BMI_control -0.3135)*(0.0637BMI_Int-0.3135)	Incidence per 1000	
Mental health Men, control	-0.00008*Age^2+0.0073*Age-0.0544	% Incidence	APMS 2014[4]
Mental health Women, control	-0.00007*Age^2+0.0062*Age+0.0034	% Incidence	
Mental Health Men, intervention	(-0.00007*Age^2+0.0062*Age+0.0034)*(0.0127*BMI_I+0.727)/(0.0127*BMI_C +0.727)	% Incidence	Sutaria et al 2018[5]
Mental Health Women, intervention	(-0.00007*Age^2+0.0062*Age+0.0034) *(0.0391*BMI_I+0.1087)/(0.0391*BMI_C+0.1087)	% Incidence	
Mental Health, diabetes	Multiply MH risk by 1.33		Chireh et al 2019[6]
Mental Health, Physical exercise	OR 0.83 high activity vs low activity. Applied to intervention arm only.		Schuch et al 2018[7]
Preterm birth <33 weeks, control	-0.0512*Age^2+3.0922*Age-37.891	Incidence per 1000	ONS 2017[8]
Preterm birth <33 weeks, intervention	(-0.0512*Age2+3.0922*Age-37.891)*(0.0514*BMI_I-0.1712)/(0.0514*BMI_C-0.1712)	Incidence per 1000	Mcdonald et al 2010[9]
Loss of earnings, men	12534BMI – 297703	£ over lifetime	

For peer review only - http://bmjopen.bmj.com/site/about/guidelines.xhtml

Parameter	Equation	Units	Source
Loss of earning, women	17695BMI - 439796	£ over lifetime	Hamilton et al 2018[10]

Table 2: Parameter equations for z-scores

Parameter	Equation	Units	Source
Mental Health Men, Intervention	(0.0054Age^2-0.191Age+1.7142)/(0.0274BMI_z_score_control+1.0011)*(0.0274BMI_z_scores_int+1.011)	Incidence per 1000	Gray et al 2015[3]
Mental Health Women, Intervention	(0.0031Age^2-0.0942Age+1.2045)/(0.084BMI_z_scores_control+0.9486)*(0.084BMI_z_scores_Int+0.9486)	Incidence per 1000	
Type 2 Diabetes Men, intervention	(-0.00007*Age^2+0.0062*Age+0.0034)/(0.1742BMI_z_scores_control+0.8964)*(0.1742BMI_z_scores_Int+0.8964)	% Incidence	Sutaria et al 2018[5]
Type 2 Diabetes Women, intervention	(-0.00007*Age^2+0.0062*Age+0.0034)*(0.1369*BMI_z_scores_I+1.0556)/(0.1369*BMI_z_scores_C+1.0556)	% Incidence	
Preterm birth <33 weeks, intervention	(-0.0512*Age2+3.0922*Age-37.891)*(0.1105*BMI_z_scores_I+0.9336)/(0.1105*BMI_z_scores_C+0.9336)	Incidence per 1000	Mcdonald et al 2010[9]

Table 3: Parameter equation for quality of life estimates

Parameter	Equation	Source
Quality of life, by age	$0.9508566+0.0212126*Male-0.0002587*Age-0.0000332*Age^2$	Maheswaran et al 2012[11]
Disutility for BMI, if BMI < 30	-0.0009BMI + 0.0189	
Disutility for BMI, if BMI > 30	-0.0036BMI+0.094	

For peer review only

For peer review only - http://bmjopen.bmj.com/site/about/guidelines.xhtml

Table 4: Input parameters used in the model

Parameter	Value	SD/SE	95% CI-Low	95% CI-High	Distribution
**Direct trial efficacy**					
BMI effect size: Physical activity	0.13	0.046	0.040	0.220	Beta
BMI effect size: Dietary quality	0.04	0.01423	0.012	0.068	Beta
BMI z-scores effect size: Physical activity	0.04	0.02	0.001	0.079	Beta
BMI z-scores effect size: Dietary quality	0.02	0.01	0.000	0.040	Beta
Prop. meeting PA guidelines control	0.13	0.0013	0.127	0.133	Beta
Prop. meeting PA guidelines intervention	0.16	0.049	0.117	0.257	Beta
Increase in Moderate to Vigorous PA	4.84	2.944	-0.940	10.610	Beta
**Relative effectiveness**					
OR for depression for Low vs High Physical Activity	0.83	0.026	0.790	0.880	Beta
OR of diabetes increasing the risk of depression	1.33	0.133	1.069	1.591	Lognormal
RR of PA on incidence of diabetes	0.74	0.074	0.595	0.885	Lognormal
**Mortality**					
Mental health: risk of death by suicide	0.001	0.000	0.001	0.001	Beta
HR of mortality those with type 2 Diabetes aged <65 years	2.978	0.190	2.605	3.350	Lognormal
HR of mortality those with type 2 Diabetes aged >65 years	1.958	0.050	1.860	2.056	Lognormal
**Relative effectiveness[a]**					
Pre-term birth relative risk for BMI P1	0.051	0.005	0.041	0.061	Beta
Pre-term birth relative risk for BMI P2	-0.171		0.111	-0.322	
MH BMI relative risk Boys P1	0.013	0.004	0.004	0.021	Beta
MH BMI relative risk Boys P2	0.727		0.914	0.540	
MH BMI relative risk girls P1	0.039	0.004	0.031	0.047	Beta
MH BMI relative risk girls P2	0.109		0.324	-0.005	
Diabetes relative risk boys P1	0.081	0.004	0.073	0.089	Beta
Diabetes relative risk boys P2	-0.845		-0.571	-0.912	
Diabetes relative risk girls P1	0.064	0.013	0.039	0.089	Beta

For peer review only - http://bmjopen.bmj.com/site/about/guidelines.xhtml

Parameter	Value	SD/SE	95% CI-Low	95% CI-High	Distribution
Diabetes relative risk girls P2	-0.314		0.167	-0.906	
Loss of earnings Men P1	12036	601.8	10856	13215.5	Gamma
Loss of earnings Men P2	-284478		-256213	-311886.5	
Loss of earnings Women P1	16689	1668.9	13418	19960.0	Gamma
Loss of earnings Women P2	-412424		-339474	-504989.1	
**Z scores [a]**					
Pre-term birth relative risk for BMI z-scores P1	0.1105	0.011	0.089	0.132	Beta
Pre-term birth relative risk for BMI z-scores P2	0.9336		-0.910	-1.841	
MH BMI z-scores relative risk Boys P1	0.0274	0.003	0.022	0.033	Beta
MH BMI z-scores relative risk Boys P2	1.0011		0.526	0.295	
MH BMI z-scores relative risk girls P1	0.084	0.008	0.068	0.100	Beta
MH BMI z-scores relative risk girls P2	0.9486		-0.452	-1.160	
Diabetes relative risk boys P1	0.1742	0.009	0.157	0.191	Beta
Diabetes relative risk boys P2	0.8964		-2.378	-3.112	
Diabetes relative risk girls P1	0.1369	0.027	0.083	0.191	Beta
Diabetes relative risk girls P2	1.0556		-0.790	-3.097	
**Proportions of population with mental health conditions**					
Chronic	0.170	0.017	0.137	0.203	Dirichlet
intermittent	0.400	0.040	0.322	0.478	Dirichlet
New and one-off	0.430	0.043	0.346	0.514	Dirichlet

[a] Risk equation is in the form: Relative risk = P1*BMI + P2

2 APPENDIX B

A breakdown of the resources used for estimating the costs of the intervention i.e. LifeLab Plus is presented below. The intervention comprises of three components:

i. Life Lab
ii. Digital App
iii. Teachers' Support (Healthy Conversation skills)

The costs are estimated for one-year that is, for the trial duration. The assumptions related to resources-used are based on a discussion with KTW on 5th June 2019.

Table 5: Life Lab

Item	Number	Comment
Number of classes per year	50	
No of pupils in each class	25	Varies between 25-32; assumed 25 for the base case
Total pupils	1250	=50 classes x 25 pupils per class
No of schoolteachers in each class	1	
Total teachers	50	

Table 6: Staff Training for Life Lab and Healthy Conversation Skills

Item	Number	Comment
Lifelab staff / training day (days)	6	Each training day / Lifelab day consists of 6 staff days to run. 4 staff days to run training and preparation and 2 days of administration.
Teacher training		
No. of Training days	5	Each training day is for 10 teachers.
Lifelab Staff days	30	=5 training days x 6 staff days / training day
Lifelab		
Number of days	50	=50x1
Lifelab Staff days	300	=50 life lab days x 6 staff day / lifelab
Scientists		Each lifelab day consists of 3 scientists
Number trained	75	Assumed each scientist does two lifelab days
Training days	3	=75 scientists /25; 25 trained per day
Lifelab Staff days	18	=3 training days x 6 staff days / training day
Training the trainers (Healthy Conversation Skills)		
Trainers	6	Assumption
Lifelab Staff days	1	Assume takes 2 hours
Showcase		All schools
Lifelab Staff days	3.5	1.5 days prep; 1 day delivery for 2 staff
Total lifelab staff days	352.5	Band 4 teacher

For peer review only - http://bmjopen.bmj.com/site/about/guidelines.xhtml

Data collection – Lifelab

Each class has two hours of data collection by two Lifelab staffs, 2 research nurses and 1 research assistant.

Table 7: Costs for data collection

Item	Number	Comment
Lifelab staff days	28.6	=2 hours x2 staff x50 (lifelab days)/7 (hours)
Research nurse	28.6	=2 hours x2 staff x50 (lifelab days)/7 (hours)
Research assistant	14.3	=2 hours x1 staff x50 (lifelab days)/7 (hours)

Table 8: Staff unit costs

Staff costs	Salary	Comment
Band 4 teacher	£34,189	i.e. £163 per day, Assumes 210 working days per year
Research assistant	£27,025	i.e. £129 per day, Assumes 210 working days per year
Research nurse Band 5	£26,231	i.e £125 per day, Assumes 210 working days per year

Table 9: Staff total costs

Staff costs	Total	Comment
Lifelab / training	£71,736	=£34,189/210x352.5*1.25; = salary / working days per year*lifelab staff days*NI and pension adjustment Assumes 210 working days per year, pension / NI contributions of 25%
Follow-up data collection	£12,641	=(£34,189*28.6 + £27,025 *28.6 + 26,231 *14.3)/210*1.25 = (salary teacher* data collection days teacher + salary research nurse* data collection days research nurse + salary research assistant*data collection days research assistant)/working days per year*NI / pension adjustment
Total	£84,377	

Table 10: Digital App development

Item	Total	Comment
Development of app	£324,000	Estimate from expert
Duration of development, year	5	As above
Duration of app, years	10	Assumption
Number of centres using app	10	Assumption
Cost per year development	£64,800	£324,000 / 5; total cost / number of years of development
Maintenance cost	£16,200	Assumed to be 25% of development cost
Cost per year of development across app lifetime per centre	£3240	£324,000 / 100; total development cost / (number of years app lasts for x number of centres)
EACH-B development for new centre	£87,445	Assumed to be 1 year of staff costs.

For peer review only - http://bmjopen.bmj.com/site/about/guidelines.xhtml

Item	Total	Comment
Number of years centre lasts	10	Assumption
Cost per year of centre	£8745	£87,445 / 10

Table 11: Summary of included costs

Item	Total	Comment
Staff costs	£84,377	
Room hire	£26,857	=400 (room hire cost)*67.14 (number of days); assumes cost of hire is £400 / day
Consumables	£15,839	Consumables include costs associated with printing, paper, classroom
Overheads	£37,970	Direct overheads based on 29% of direct care salary costs and indirect overheads based on 16% direct care salary costs (As used in Curtis et al 2019 for overheads for healthcare professionals)
Maintenance of app	£16,200	See above table
Capital cost, development of app	£3240	See above
Capital cost, development of EACH-B	£8745	See above

Table 12: Total costs

Item	Total	Comment
Total cost	£193,228	Sum of all of the above costs
Cost per pupil	£154.58	=£193,228/1250; (total cost /total number of pupils)

For peer review only

3 APPENDIX C

Figure 1: Cost-Effectiveness Acceptability Curve (CEAC)

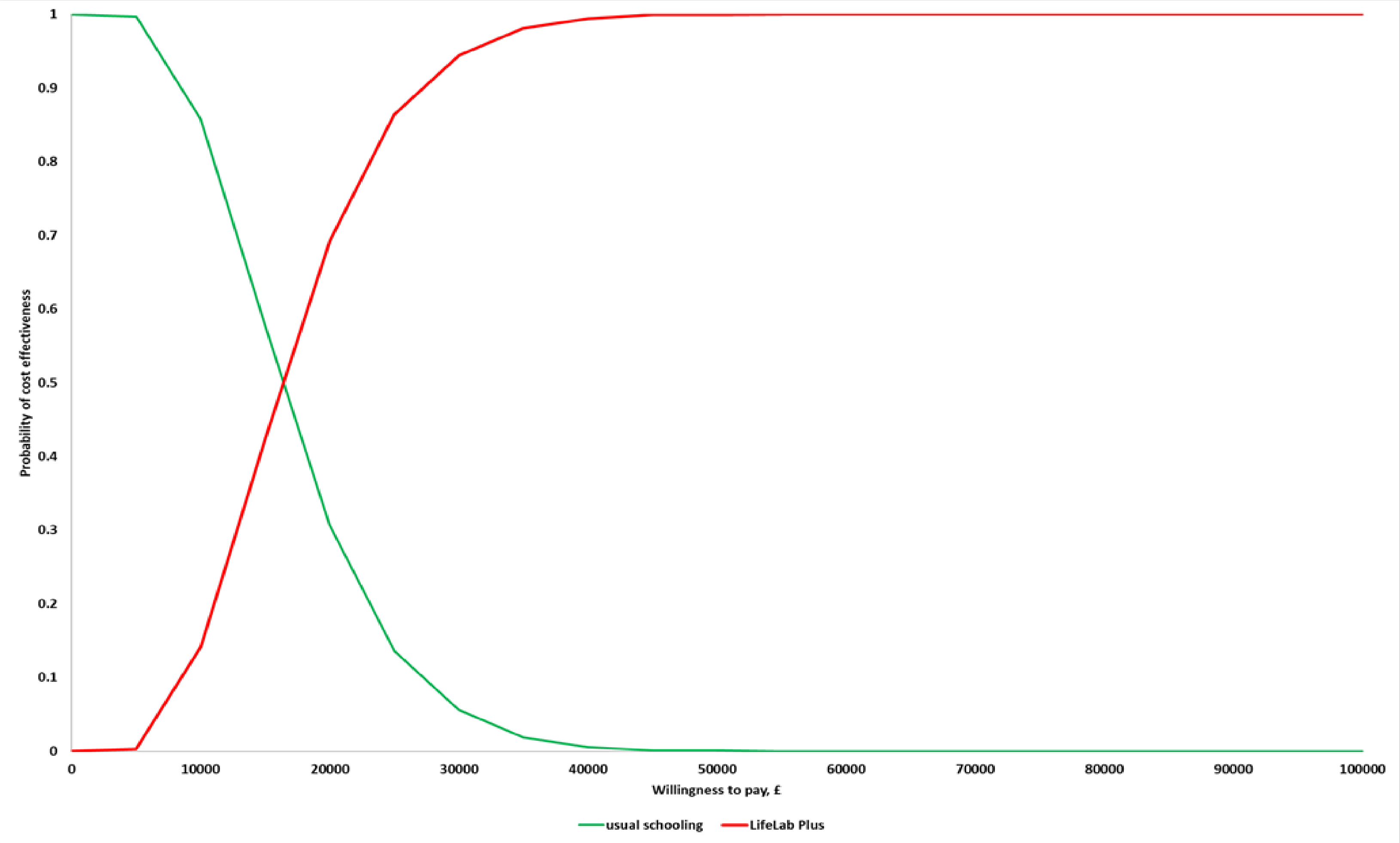

For peer review only - http://bmjopen.bmj.com/site/about/guidelines.xhtml

REFERENCES

1. NHS Digital. Health Survey for England 2016. 2017.
2. Sharma M, Nazareth I, Petersen I. Trends in incidence, prevalence and prescribing in type 2 diabetes mellitus between 2000 and 2013 in primary care: a retrospective cohort study. *BMJ Open* 2016;6(1):e010210. doi: 10.1136/bmjopen-2015-010210 [published Online First: 2016/01/16]
3. Gray N, Picone G, Sloan F, et al. Relation between BMI and diabetes mellitus and its complications among US older adults. *South Med J* 2015;108(1):29-36. doi: 10.14423/SMJ.0000000000000214 [published Online First: 2015/01/13]
4. McManus S BP, Jenkins R, Brugha T. Mental health and wellbeing in England: Adult Psychiatric Morbidity Survey 2014. 2016.
5. Sutaria S, Devakumar D, Yasuda SS, et al. Is obesity associated with depression in children? Systematic review and meta-analysis. *Arch Dis Child* 2019;104(1):64-74. doi: 10.1136/archdischild-2017-314608 [published Online First: 2018/07/01]
6. Chireh B, Li M, D'Arcy C. Diabetes increases the risk of depression: A systematic review, meta-analysis and estimates of population attributable fractions based on prospective studies. *Prev Med Rep* 2019;14:100822. doi: 10.1016/j.pmedr.2019.100822 [published Online First: 2019/03/01]
7. Schuch FB, Vancampfort D, Richards J, et al. Exercise as a treatment for depression: A meta-analysis adjusting for publication bias. *J Psychiatr Res* 2016;77:42-51. doi: 10.1016/j.jpsychires.2016.02.023 [published Online First: 2016/03/16]
8. Office of National Statistics. Births in England and Wales: 2017. 2018.
9. McDonald SD, Han Z, Mulla S, et al. Overweight and obesity in mothers and risk of preterm birth and low birth weight infants: systematic review and meta-analyses. *BMJ* 2010;341:c3428. doi: 10.1136/bmj.c3428 [published Online First: 2010/07/22]
10. Hamilton D, Dee A, Perry IJ. The lifetime costs of overweight and obesity in childhood and adolescence: a systematic review. *Obes Rev* 2018;19(4):452-63. doi: 10.1111/obr.12649 [published Online First: 2017/12/23]
11. Maheswaran H, Petrou S, Rees K, et al. Estimating EQ-5D utility values for major health behavioural risk factors in England. *J Epidemiol Community Health* 2013;67(2):172-80. doi: 10.1136/jech-2012-201019 [published Online First: 2012/07/31]

For peer review only

For peer review only - http://bmjopen.bmj.com/site/about/guidelines.xhtml

CHEERS checklist—Items to include when reporting economic evaluations of health interventions

Section/item	Item No	Recommendation	Reported on page No/ line No
**Title and abstract**			
Title	1	Identify the study as an economic evaluation or use more specific terms such as "cost-effectiveness analysis", and describe the interventions compared.	page 1, line 1
Abstract	2	Provide a structured summary of objectives, perspective, setting, methods (including study design and inputs), results (including base case and uncertainty analyses), and conclusions.	page 2, lines 29 to 54
**Introduction**			
Background and objectives	3	Provide an explicit statement of the broader context for the study.	page 4, line 110 to page 5, line 137
		Present the study question and its relevance for health policy or practice decisions.	page 5, line 139 to 149
**Methods**			
Target population and subgroups	4	Describe characteristics of the base case population and subgroups analysed, including why they were chosen.	page 6, line 172 to 175
Setting and location	5	State relevant aspects of the system(s) in which the decision(s) need(s) to be made.	page 4, line 125 to page 5, line 137 and line 145 to line 149;
Study perspective	6	Describe the perspective of the study and relate this to the costs being evaluated.	page 11, line 348 to 350; page 14, line 438;
Comparators	7	Describe the interventions or strategies being compared and state why they were chosen.	page 5, line 139 to 149; page 6, line 174 to 175
Time horizon	8	State the time horizon(s) over which costs and consequences are being evaluated and say why appropriate.	page 7, line 214 to line 217 and line 222
Discount rate	9	Report the choice of discount rate(s) used for costs and outcomes and say why appropriate.	page 11, line 350
Choice of health outcomes	10	Describe what outcomes were used as the measure(s) of benefit in the evaluation and their relevance for the type of analysis performed.	page 7, line 201 to line 207
Measurement of effectiveness	11a	*Single study-based estimates:* Describe fully the design features of the single effectiveness study and why the single study was a sufficient source of clinical effectiveness data.	not applicable
	11b	*Synthesis-based estimates*: Describe fully the methods used for identification of included studies and synthesis of clinical effectiveness data.	page 8, line 229 to page 10, line 306; supplement appendix A
Measurement and valuation of preference based outcomes	12	If applicable, describe the population and methods used to elicit preferences for outcomes.	not applicable
Estimating resources and costs	13a	*Single study-based economic evaluation:*Describe approaches used to estimate resource use associated with the alternative interventions. Describe primary or secondary research methods for valuing each resource item in terms of its unit cost. Describe any adjustments made to approximate to opportunity costs.	not applicable

For peer review only

Section/item	Item No	Recommendation	Reported on page No/ line No
	13b	*Model-based economic evaluation:* Describe approaches and data sources used to estimate resource use associated with model health states. Describe primary or secondary research methods for valuing each resource item in terms of its unit cost. Describe any adjustments made to approximate to opportunity costs.	page 10, line 309 to page 11, line 339; pages 17-18, table 1; supplement Appendix B
Currency, price date, and conversion	14	Report the dates of the estimated resource quantities and unit costs. Describe methods for adjusting estimated unit costs to the year of reported costs if necessary. Describe methods for converting costs into a common currency base and the exchange rate.	page 11, line 324 to 325; page 11, line 337 to 339;
Choice of model	15	Describe and give reasons for the specific type of decision-analytical model used. Providing a figure to show model structure is strongly recommended.	page 5, line 153 to page 6, line 162; figure 1.
Assumptions	16	Describe all structural or other assumptions underpinning the decision-analytical model.	Page 6, line 159 to 160; Page 6, line 181 to 182; Page 7, line 210 to 211 and line 216 to 220; Page 8, line 242 to 244, 247 to 250, 253 to 254; Page 9, line 261 to 262, line 267 to 273, line 281 to 283, 286 to 288 page 10 line 306; Page 10, line 312 to 319; Page 11, line 327 to line 334; Page 12, line 363 to line 367; Page 13, line 406 to 407; Page 14, line 431 to 433, line 441 to 442.
Analytical methods	17	Describe all analytical methods supporting the evaluation. This could include methods for dealing with skewed, missing, or censored data; extrapolation methods; methods for pooling data; approaches to validate or make adjustments (such as half cycle corrections) to a model; and methods for handling population heterogeneity and uncertainty.	Page 11, line 342 to 345; page 12, line 358 to line 369; Supplement Appendix A, Table 4
**Results**			
Study parameters	18	Report the values, ranges, references, and, if used, probability distributions for all parameters. Report reasons or sources for distributions used to represent uncertainty where appropriate. Providing a table to show the input values is strongly recommended.	Page 7, line 209 to page 12, line 369; Pages 17 to 18, table 1; supplement Appendix A, Table 1 to 4.
Incremental costs and outcomes	19	For each intervention, report mean values for the main categories of estimated costs and outcomes of interest, as well as mean differences between the comparator groups. If applicable, report incremental cost-effectiveness ratios.	page 12, line 381 to 385; pages 19 to 20, table 2
Characterising uncertainty	20a	*Single study-based economic evaluation:*Describe the effects of sampling uncertainty for the estimated incremental cost and incremental effectiveness	not applicable

For peer review only

Section/item	Item No	Recommendation	Reported on page No/ line No
		parameters, together with the impact of methodological assumptions (such as discount rate, study perspective).	
	20b	*Model-based economic evaluation:* Describe the effects on the results of uncertainty for all input parameters, and uncertainty related to the structure of the model and assumptions.	page 12, line 388 to page 13, line 399; figures 2 and 3; pages 19 to 20, table 2; supplement Appendix C figure 1
Characterising heterogeneity	21	If applicable, report differences in costs, outcomes, or cost-effectiveness that can be explained by variations between subgroups of patients with different baseline characteristics or other observed variability in effects that are not reducible by more information.	not applicable
**Discussion**			
Study findings, limitations, generalisability, and current knowledge	22	Summarise key study findings and describe how they support the conclusions reached. Discuss limitations and the generalisability of the findings and how the findings fit with current knowledge.	page 13, line 403 to page 15, line 476
**Other**			
Source of funding	23	Describe how the study was funded and the role of the funder in the identification, design, conduct, and reporting of the analysis. Describe other non-monetary sources of support.	page 16, line 499 to line 505
Conflicts of interest	24	Describe any potential for conflict of interest of study contributors in accordance with journal policy. In the absence of a journal policy, we recommend authors comply with International Committee of Medical Journal Editors recommendations.	page 15, line 507 to line 510

For consistency, the CHEERS statement checklist format is based on the format of the CONSORT statement checklist

For peer review only
